# Geomag Mimetic Dynamically Reconfigurable POSS Framework Enables Antifatigue Solid Electrolyte Interphases for Lithium Metal Anodes

**DOI:** 10.1002/advs.76111

**Published:** 2026-07-21

**Authors:** Tianyi Wang, Wei Gu, Yu Liu, Di He, Zairan Xiao, Zixia Lin, Tao Huang, Jiabao Li, Hui Chong, Yaojie Lei, Xiaobo Zheng, Bing Sun, Guoxiu Wang, Chengyin Wang

**Affiliations:** ^1^ School of Chemistry and Materials Yangzhou University Yangzhou Jiangsu P. R. China; ^2^ Centre For Clean Energy Technology School of Mathematical and Physical Sciences Faculty of Science University of Technology Sydney Sydney New South Wales Australia; ^3^ Test Center of Yangzhou University Yangzhou Jiangsu P. R. China; ^4^ Jiangsu Provincial Key Laboratory of Green & Functional Materials and Environmental Chemistry Yangzhou Jiangsu P. R. China

**Keywords:** antifatigue interface design, dynamic solid electrolyte interphase, lithium metal anodes, polyhedral oligomeric silsesquioxane (POSS), self‐healing interfacial chemistry

## Abstract

Lithium metal anodes are fundamentally limited by mechanical fatigue and structural instability of the solid electrolyte interphase (SEI) during repeated plating and stripping. Here, we introduce a stress‐adaptive molecular interphase featuring autonomously reconfigurable ion‐transport channels inspired by modular “Geomag”‐like architecture. The interphase is constructed from polyhedral oligomeric silsesquioxane (POSS) nanocages bridged by reversible melamine‐mediated hydrogen bonds, forming dynamic secondary junctions that enable stress‐triggered disconnect–reconnect behavior. This reversible connectivity allows the SEI to dissipate local strain while actively opening and closing Li^+^ transport pathways, thereby homogenizing ion flux and stabilizing the interfacial electric field. Antifatigue architecture effectively suppresses dendrite nucleation and prolongs interfacial stability. Symmetric Li || Li and Li || Cu cells verify enhanced reversibility, while Li || LTO full cells sustain ultralong cycling exceeding 2000 h. Pouch‐type lithium metal cells further demonstrate stable electrochemical performance under practical conditions. This work establishes a general molecular design paradigm for autonomously self‐regulating SEIs toward durable lithium metal cells.

## Graphical abstract

A Geomag‐inspired dynamically reconfigurable POSS framework is constructed to form an antifatigue solid electrolyte interphase (SEI) for lithium metal anodes. The rigid POSS nanocages and reversible hydrogen‐bonded junctions enable adaptive stress dissipation and stable Li^+^ transport during repeated plating/stripping, thereby suppressing dendrite growth, mitigating interfacial fatigue accumulation, and significantly enhancing long‐term electrochemical stability.

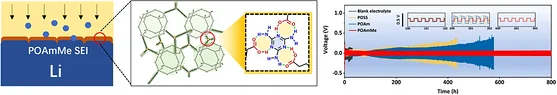

## 1 Introduction

The accelerated demand for electric vehicles and grid‐scale energy storage systems has underscored the limitations of conventional lithium‐ion batteries, particularly those employing graphite anodes [1]. To achieve next‐generation high‐energy‐density storage, lithium (Li) metal anodes (LMAs) have reemerged as a compelling alternative due to their exceptionally high theoretical specific capacity (3,860 mAh g^−^^1^) and the lowest electrochemical potential (−3.04 V vs. SHE) [2]. However, the practical deployment of Li metal anodes remains severely constrained by uncontrolled Li dendrite growth, which not only undermines electrochemical reversibility but also poses significant safety concerns [3]. The root cause of dendrite proliferation lies in the instability of the solid electrolyte interphase (SEI) formed on the Li surface [4]. During repeated plating and stripping, the original SEI, which is typically brittle and mechanically rigid, cannot accommodate the substantial volume changes (∼100%) of Li metal. Its mechanical rupture exposes fresh reactive Li, triggering continuous parasitic side reactions and the formation of isolated “dead Li”. This leads to persistent electrolyte depletion, increasing internal resistance, and irreversible capacity loss. Therefore, engineering a robust, fatigue‐resistant, and self‐healing SEI has become a central strategy to stabilize Li metal interfaces under dynamic cycling conditions [5]. Conventional SEI‐regulation strategies have largely focused on constructing rigid or inorganic‐rich interphases, such as LiF‐, Li_2_O‐, Li_3_N‐rich layers and ceramic/artificial coatings, to mechanically block dendrite penetration and suppress continuous electrolyte decomposition. These strategies are effective in enhancing electronic insulation and improving interfacial chemical stability. In parallel, recent electrolyte‐engineering studies have demonstrated that regulating solvent–ion configurations, nonflammable electrolyte chemistry, ether/ester solvent structures, and trace additive‐mediated solvation environments can effectively modulate Li plating/stripping behavior and SEI formation [6, 7, 8, 9]. However, such conventional rigid/inorganic interphases are generally static and mechanically rigid, which often limits their ability to accommodate repeated Li plating/stripping‐induced volume fluctuation. Once local cracks are generated, fresh Li is exposed to the electrolyte, leading to repeated SEI rupture/reformation, heterogeneous Li^+^ flux, impedance accumulation, and dead‐Li formation. Therefore, beyond simply increasing SEI rigidity, developing a stress‐adaptive interphase that can dissipate local strain, reconstruct its interfacial structure, and restore Li^+^ transport pathways is highly desirable for mitigating the mechanical fatigue of Li metal anodes.

In 2024, Xiong et al. reported a polyether‐urethane‐based SPE featuring abundant ether and carbonyl groups to promote lithium salt dissociation and enhance ionic conductivity. Dynamic disulfide and hydrogen bonds imparted self‐healing ability, enabling effective repair of solid–solid interfacial defects during cycling [10]. In 2025, Gao et al. developed a novel lithium supplementation strategy at the cell level, in which an organic lithium salt is externally added and decomposes during formation to release active Li^+^ while expelling organic ligands as gases [11]. This in situ process enables effective SEI repair without cell disassembly, thereby restoring lithium inventory and interfacial stability. Guided by machine learning, lithium trifluoromethanesulfinate (LiSO_2_CF_3_) was identified as an optimal candidate, exhibiting excellent electrochemical activity, solubility, and product characteristics, ultimately enabling high‐capacity, long‐life anode‐free and commercial battery systems [11]. In our previous studies, we also found that the rational incorporation of natural polymeric materials plays a positive role in tuning the chemical, physical, and mechanical properties of the SEI on metal anodes [12]. Moreover, their inherent self‐assembly behavior imparts self‐healing capability to the interphase, ensuring dynamic interfacial stabilities of metal anodes [13].

**FIGURE 1:**
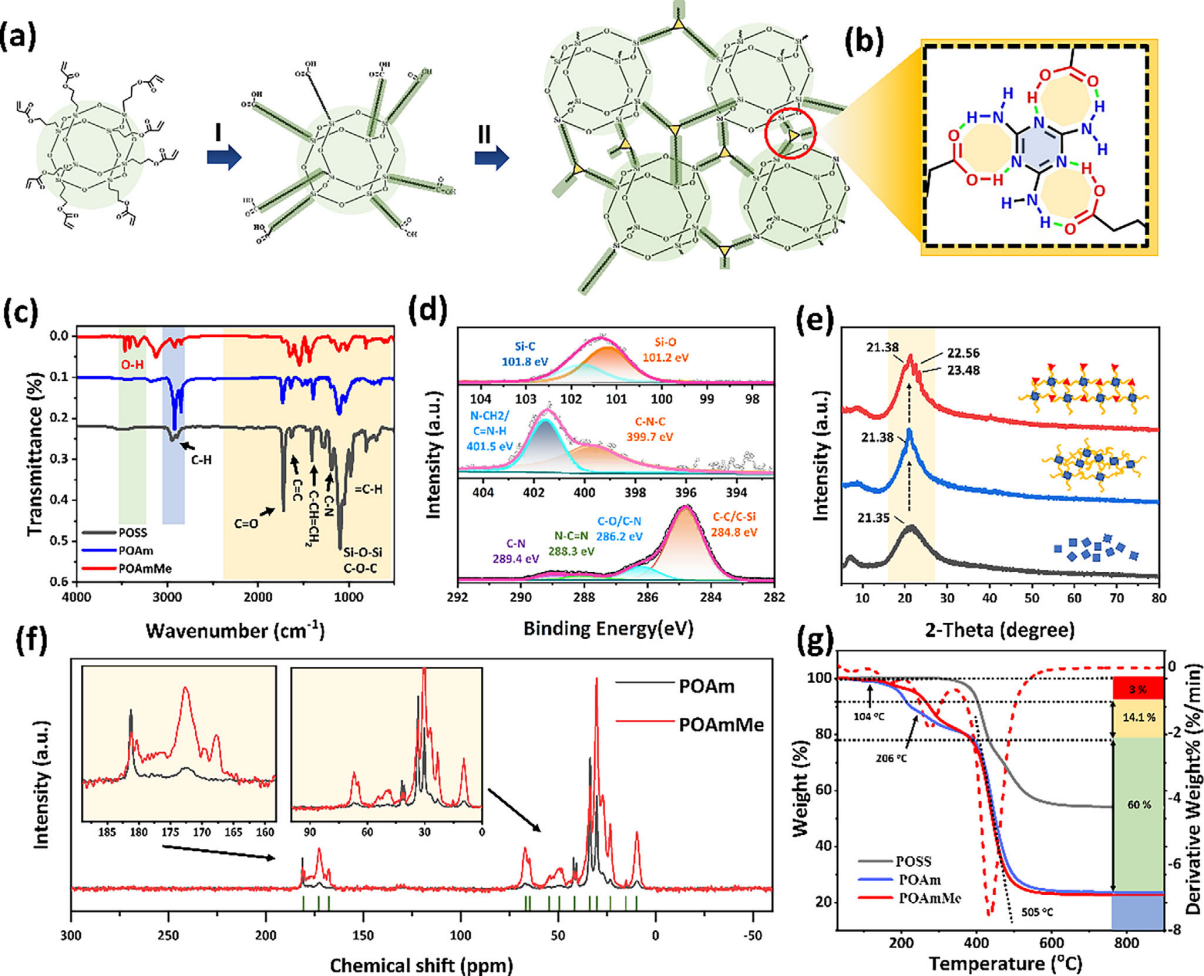
Characterization of POAmMe structure. (a) The assembly and synthesis process of POAmMe 3D skeleton and (b) molecular structure of “Geomag” like secondary conjunction. (c) FT‐IR spectra of POSS, POAm, and POAmMe additives. (d) High resolution XPS spectrum of Si 2*p*, N 1*s*, and C 1*s* of prepared POAmMe. (e) XRD profiles of original POSS, POAm, and POAmMe. (f) Solid‐state ^13^C NMR spectrum of POAm and POAmMe. (g) Comparative TGA and DTG curves of POSS, POAm, and POAmMe measured under N_2_ at a heating rate of 10°C min^−^^1^, showing the distinct thermal‐decomposition behaviors of the three samples.

Polyhedral oligomeric silsesquioxane (POSS) can serve as a series of nano‐building blocks because they have emerged as a class of unique inorganic building blocks for constructing functional materials [14]. These well‐defined nanostructures can be incorporated into polymer matrices to create novel hybrid materials with enhanced properties [15, 16]. POSS molecules feature a cage‐like, three‐dimensional architecture with the general formula (RSiO_1_._5_)_n_ (n > 6) [17]. Among them, octasilsesquioxane (R_8_Si_8_O_12_, T8) is the most extensively studied branch, consisting of a rigid cubic silica core (∼0.53 nm per side) and eight corner organic groups. This distinctive structure imparts POSS with unique characteristics for the design of POSS‐based hybrid polymers. Moreover, the low molecular weight and discrete geometry of the POSS core facilitate uniform dispersion within polymer matrices, improving compatibility with the original SEI layer and reducing crystallinity, thereby enhancing ionic conductivity [18]. In this work, inspired by the popular educational toy “Geomag”, we design an intelligent, anti‐fatigue, self‐healing SEI interface based on POSS. Geomag is a modular magnetic construction system composed of rigid rods and detachable magnetic joints, allowing repeated assembly, disassembly, and structural reconfiguration under external force. Inspired by this reconfigurable architecture, rigid POSS nanocages are used as primary nodes, flexible Amino linkers serve as molecular rods, and melamine‐mediated hydrogen‐bonded junctions function as reversible secondary nodes. Distinct from conventional rigid inorganic SEI layers that mainly rely on static mechanical blocking, the POAmMe‐derived SEI is designed as a dynamically reconfigurable molecular interphase. The rigid POSS nanocages provide mechanical reinforcement, while the reversible melamine‐mediated hydrogen‐bonded junctions act as dynamic secondary nodes for stress dissipation and structural recovery. Such a rigid–dynamic coupled architecture allows the SEI to maintain interfacial integrity under repeated Li plating/stripping while adaptively regulating local Li^+^ transport, thereby addressing the mechanical fatigue issue that is difficult to overcome by purely rigid SEI designs. As illustrated in Figure 1a, the three‐dimensional cross‐linked structure consists of POSS nanocage as the primary node and melamine as the secondary node, interconnected by 11‐aminoundecanoic acid (denoted as Amino) linkers. The terminal carboxyl group of the Amino acid forms stable tridentate hydrogen bonds with melamine. Upon volume expanding during lithium deposition, these secondary “magnetic‐like” connections can reversibly dissociate, similar to the detachable joints in “Geomag”, allowing the SEI to absorb internal stress and prevent cracking. Rapid local volumetric expansion can also lead to temporary closure of POSS‐based ion channels, thereby halting lithium‐ion flux in those regions and mitigating dendrite growth. Once the stress is relieved (e.g., during discharge), the secondary nodes spontaneously reconnect, restoring the structural integrity of the SEI. Furthermore, under uneven stress conditions, localized changes in crystallinity modulate ionic conductivity and electric field distribution, providing a dynamic mechanism to suppress dendrite nucleation and propagation.

## 2 Results and Discussion

### 2.1 Characterization of the POAmMe Anti‐Fatigue 3D Skeleton

The POSS‐Amino‐Melamine (POAmMe) polymer additive was synthesized via a straightforward two‐step process. First, the POSS‐Amino (POAm) intermediate was prepared via aza‐Michael addition between acrylate‐functionalized POSS and 11‐aminoundecanoic acid (Amino) in anhydrous DMSO at 65°C. The monofunctional primary amine of Amino reacts with the acrylate termini of POSS, while the terminal carboxyl group is retained for subsequent supramolecular assembly. The monofunctional nature of Amino, together with the absence of observable gelation or insoluble fractions during purification, suggests that extensive permanent POSS–POSS covalent cross‐linking was effectively suppressed. POAm was then assembled with melamine through directional hydrogen bonding between terminal carboxyl groups and the triazine core, yielding the dynamic POAmMe supramolecular framework (Figure 1b). The ring formation was driven by directional hydrogen bonding between the terminal carboxyl groups of the branched chains and the triazine core of melamine. On average, each melamine molecule binds to three carboxyl termini, acting as a “Secondary node” that enables the construction of a dynamic cross‐linked network. Scanning electronic microscopy (SEM, Figure S1) and transmission electronic microscopy (TEM, Figure S2) images confirm that synthesized POAmMe has high stability and surface ratio. The evolution of functional groups during the construction of POAmMe was investigated by Fourier‐transform infrared spectroscopy (FT‐IR). As shown in Figure 1c, acrylo‐POSS exhibits aliphatic C─H stretching vibrations at 2952 and 2888 cm^−^^1^, together with characteristic acrylate‐related C═O and C═C stretching vibrations at around 1720 and 1636 cm^−^^1^, respectively. The absorption at 1409 cm^−^^1^ is associated with vinyl‐related C─H bending, while the strong band at approximately 1100 cm^−^^1^ is assigned to the Si─O─Si stretching vibration of the POSS cage. After aza‐Michael addition with Amino, the acrylate C═C signal was markedly weakened, accompanied by enhanced aliphatic C─H vibrations from the introduced long alkyl chains and the appearance/strengthening of C─N‐related vibrations. After melamine incorporation, the broadened N─H/O─H band and C─N/C═N‐related vibrations became more evident, supporting the formation of hydrogen‐bond‐mediated supramolecular interactions between POAm and melamine. These spectral changes collectively support the successful construction of the POAmMe supramolecular framework.The chemical composition of POAm and POAmMe additives were analyzed by X‐ray photoelectron spectroscopy (XPS, Figure S3). The XPS spectrum of the Si 2*p* peak in Figure 1d reveals two peaks at 101.8 and 101.2 eV, which correspond to Si─C and Si─O bonds on the POSS cage [19], respectively. The peak area ratio of 1:3 is consistent with the molecular structure of the POSS cage. The peaks observed at 399.7 and 401.5 eV correspond to C─N─C and N─CH_2_/C═NH, respectively. The C 1*s* spectra also show multiple peaks, corresponding to C─C/C─Si, C─O/C─N, N─C═N, and C─N, confirming the successful connection of the carbon skeleton.

The self‐assembly behavior of the POAmMe 3D framework and the successful construction of secondary linking nodes were investigated by X‐ray diffraction (XRD). As shown in Figure 1e, acrylo‐POSS exhibits characteristic diffraction peaks at 7.2° (d = 12.2 Å) and 21.5° (d = 4.1 Å), consistent with the previously reported POSS cage packing curve [20]. Upon grafting with Amino, the crystallinity of the intermediate POAm notably increased, as evidenced by the emergence of new peaks at 22.5° (d = 3.9 Å) and 23.3° (d = 3.8 Å), corresponding to ordered alkyl chain stacking (Figure S4). The introduction of long alkyl chains facilitates the spatial alignment and self‐aggregation of POSS units through van der Waals interactions. Notably, further coordination with melamine results in an additional increase in crystallinity, suggesting the formation of a more ordered three‐dimensional framework. This enhancement is attributed to hydrogen bonding and dipolar interactions between the carboxyl terminals of POAm and the Amino groups in melamine, which promote directional intermolecular alignment and framework assembly. These findings confirm that melamine effectively drives supramolecular organization, thereby establishing a robust ion‐conductive scaffold capable of providing widened and interconnected channels for Li^+^ transport. High‐resolution mass spectrometry confirmed the successful formation of POAm (Figure S5). The molecular structure of POAm was further examined by ^1^H NMR spectroscopy using acrylo‐POSS as the reference (Figures S6 and S7). After aza‐Michael addition, the acrylate vinyl proton signals at δ ≈ 5.8–6.5 ppm were largely diminished, accompanied by the appearance of pronounced aliphatic proton signals at δ = 1.57, 1.51, 1.43, 1.31, 1.25, 1.08, and 0.90–0.81 ppm, which are associated with the grafted 11‐aminoundecanoic acid chains and POSS‐linked organic substituents. These spectral changes indicate effective Amino grafting onto the POSS framework and a high substitution degree of POAm. The subsequent supramolecular assembly between POAm and melamine was analyzed by solid‐state ^1^ ^3^C NMR (Figure 1f). The reduced/shifted resonances at around 180 and 169 ppm after melamine incorporation suggest interactions between the terminal carboxyl groups of POAm and the melamine units, supporting the formation of hydrogen‐bond‐mediated secondary junctions within the POAmMe framework. As shown in Figure 1f, after the combination of melamine, the peaks originally belonging to the Amino terminal free carboxyl group and Melamine at 180 ppm and 169 ppm both decreased, proving the successful occurrence of proton transfer. Thermogravimetric analysis (TGA) was employed to evaluate the thermal stability of POSS, POAm, and POAmMe (Figures S8–S10). As shown in Figure 1g, the TGA/DTG curves of these three samples exhibit distinct thermal‐decomposition behaviors associated with their different molecular and supramolecular structures. Compared with POSS and POAm, POAmMe shows a more complex multistep weight‐loss process, reflecting the hierarchical framework formed by POSS nanocages, Amino side chains, and melamine‐mediated supramolecular junctions. For POAmMe, a minor weight loss of ∼3.3% below 120°C is attributed to the evaporation of physically adsorbed water or residual solvents. A second weight‐loss step (∼14.1%) centered at ∼206°C corresponds to the thermal decomposition of flexible Amino undecanoic acid side chains or partially unreacted terminal groups. A dominant weight loss of ∼60% occurs at ∼505°C, reflecting the collapse of the cross‐linked organic framework comprising melamine‐associated carboxyl‐terminated chains. The POAmMe framework retains ∼22.6% of its original weight at 800°C, which can be attributed to the formation of thermally stable silica‐like residues derived from the POSS core. These results further support the successful stepwise construction of the POAmMe supramolecular framework. The tensile stress–strain curves further reveal that POAmMe exhibits enhanced mechanical robustness compared with POSS and POAm, with higher tensile strength and larger fracture strain, suggesting that the melamine‐mediated supramolecular junctions improve the toughness and deformation tolerance of the framework (Figure S11). Furthermore, the cut‐healing experiment and ionic‐conductivity recovery analysis demonstrate the self‐healing capability of POAmMe in ether electrolyte at room temperature (Figures S12 and S13). The original POAmMe exhibited an ionic conductivity of 0.953 mS cm^−^^1^, which slightly decreased to 0.925 mS cm^−^^1^ after cutting due to partial disruption of the ion‐transport pathway. After healing, the ionic conductivity recovered to 0.948 mS cm^−^^1^, corresponding to an ionic‐conductivity recovery efficiency of 99.48%. These results indicate that POAmMe can effectively restore its ion‐transport capability after mechanical damage, providing quantitative support for its application in self‐healing SEI films.

### 2.2 Plating‐Stripping Performance of Li Metal Anodes

**FIGURE 2:**
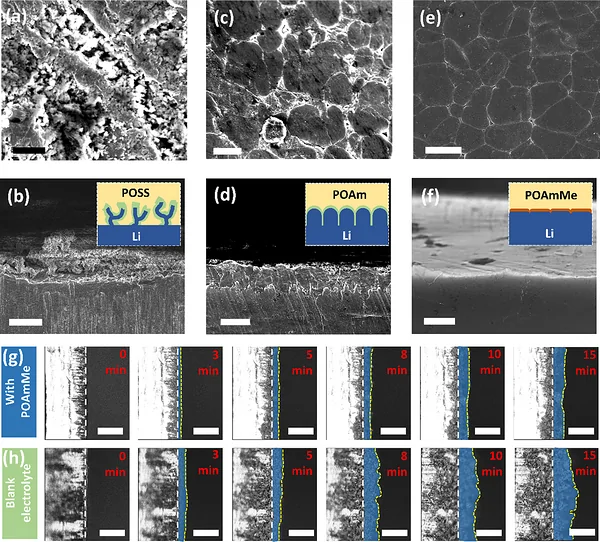
Postmortem analyses on retrieved Li metal anodes. Surface and cross‐section morphologies of Li metal foil with POSS (a,b), POAm (c,d) and POAmMe (e,f) additives being deposited‐stripped for 30 cycles at 3 mA cm^−2^ for the capacity of 1 mAh cm^−2^. Scale bars (a,c,e), 2 µm; scale bars (b,d,f), 10 µm. Operando characterization of Li‐plating in a transparent cell with (g) and without (h) POAmMe additive from 0 to 15 min. Scale bars (g,h), 300 µm.

To investigate the effect of POSS‐based additives on lithium nucleation behavior, we conducted Li plating tests in Li || Cu half cells (1 mAh cm^−^^2^ at 1 mA cm^−^^2^) and examined the morphology of deposited lithium nuclei on Cu foil using SEM. As shown in Figure S14, Li nuclei formed in the bare ether electrolyte exhibit smaller diameters and irregular clustering, suggesting high nucleation overpotential and non‐uniform ion flux. Upon introducing the POSS additive, the average Li bud size markedly increases and the distribution becomes narrower, indicating enhanced nucleation uniformity. Notably, the POAmMe‐modified system exhibits the largest average nucleus diameter (centered around 3–4 µm) with minimal population in the submicron regime. The progressive increase in nucleus size from blank electrolyte to POSS, POAm, and POAmMe systems reflects a reduced tendency toward dendritic growth, which is typically associated with fine, uneven nucleation. These observations confirm that POSS‐based molecular additives promote low‐barrier, spatially homogeneous lithium nucleation, laying a foundation for stable lithium deposition in metal cells [21]. Figure S15 compares digital photos of Li metal anodes retrieved from Li || Li symmetric cells after 50 cycles at 3 mA cm^−^^2^ for 1 mAh cm^−^^2^ in different electrolytes. Compared with the severely corroded surface in the blank and POSS‐containing electrolytes, the POAmMe‐based system demonstrates the smoothest and most intact Li surface, indicating superior interfacial protection. The morphological evolution of the Li metal anode was examined by SEM after 30 plating/stripping cycles at a current density of 3 mA cm^−^^2^ with a capacity of 1 mAh cm^−^^2^. In the control group with POSS additive, the Li surface exhibits severe structural degradation, characterized by abundant mossy‐like dendritic protrusions and extensive surface damage (Figure 2a and Figure S16). Cross‐sectional imaging further reveals the presence of large voids and a porous structure in the deposited Li layer, indicative of unstable Li growth and accumulation of inactive “dead Li” (Figure 2b). In contrast, the introduction of POAm as an electrolyte additive improves surface uniformity, with the deposited Li forming denser and more nodular structures (Figure 2c and Figure S17). The corresponding cross‐section shows increased areal density of the plated Li layer, although inter‐nodular gaps remain apparent (Figure 2d). Remarkably, the POAmMe‐modified electrolyte yields the most uniform and compact Li deposition. As demonstrated in Figure 2e and Figure S18, a highly ordered and densely packed arrangement of dome‐like Li deposits with a smooth surface morphology can be clearly observed. The cross‐sectional image confirms the formation of a homogeneous and void‐free layer of freshly deposited metallic Li (Figure 2f).

To further investigate the real‐time plating behavior, transparent Li || Li symmetric cells were assembled and in situ monitored during electrochemical depositing processes. With the POAmMe additive, the growth front of the Li layer remains flat over time, and no filamentous Li structures are observed up to 15 min of plating (Figure 2g). In contrast, the cell using a blank electrolyte exhibits improved morphological instability, in which mossy and whisker‐like dendrites begin to appear after 8 min, and by 15 min, the deposited layer becomes nearly twice as thick and structurally heterogeneous (Figure 2h). This dendrite suppression effect is attributed to the uniformly distributed ─COO^−^ functional groups exposed upon the cleavage of secondary dots in the POAmMe structure, which enhance Li^+^ transference and prolong Sand's time, thereby mitigating local ion depletion and dendritic growth.

### 2.3 Structure of SEI Layer

**FIGURE 3:**
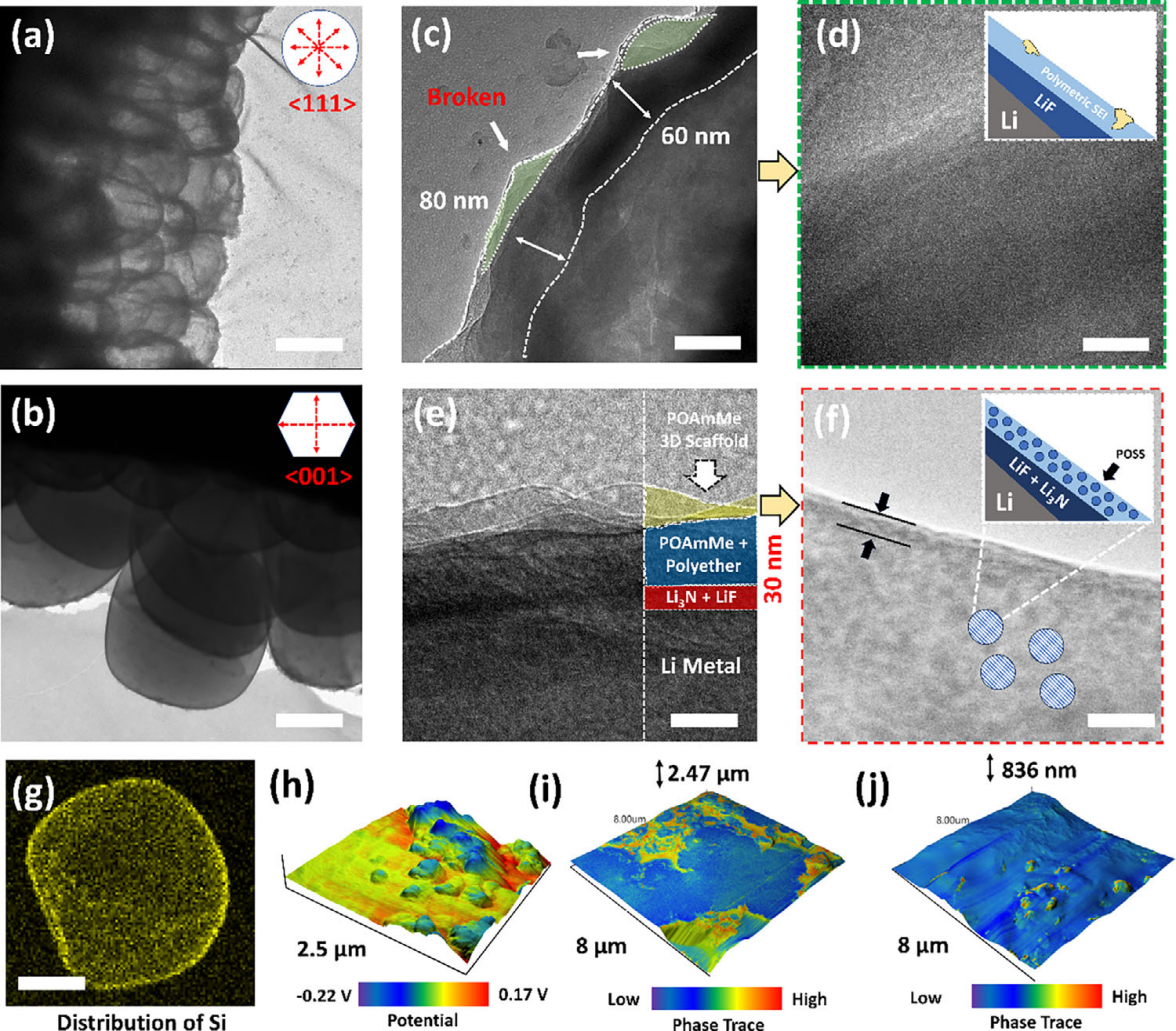
Cryo‐TEM characterization of Li deposits and the influence of SEI nanostructure on interfacial viscoelasticity and potential distribution. Morphologies of Li nuclei deposited on Cu mesh without (a) and with (b) POAmMe additive. Scale bars (a,b), 1 µm. (c,d) Cross‐section structure of the SEI layer formed in blank electrolyte and enlarged Cryo‐TEM image. Scale bar (c), 100 nm; Scale bar (d), 20 nm. (e) Cross‐sectional diagram of POAmMe hybrid SEI layer formed with an additive consisting of three layers labeled by I, II, and III. (f) Magnified image of the upper layer in which blue circles are indexed to POSS nano cages. Scale bars (d,f), 5 nm. (g) EDS mapping of Si signal on one Li nuclei covered by POAmMe SEI layer. Scale bar (g), 1 µm. (h) KPFM images indicating EV distribution of Li metal foil. Phase distribution corresponding to viscoelasticity of SEI layer formatted on Li metal anode (i) with and (j) without POAmMe additive.

To elucidate the composition and structural characteristics of the SEI layer, we performed cross‐sectional cryogenic transmission electron microscopy (Cryo‐TEM) analysis. Li metal was electrodeposited onto a copper TEM grid with a capacity of 0.5 mAh cm^−^^2^ at a current density of 1 mA cm^−^^2^, and imaging was conducted at −185 °C with low‐dose exposure mode to preserve the native morphology of the SEI [22]. As shown in Figure 3a, Li deposited in the blank ether electrolyte (1 M LiTFSI in DOL/DME) exhibits a characteristic ellipsoidal morphology, with an average size of approximately 2 µm × 1 µm. This circular cross‐section suggests that the Li nuclei preferentially grow along the <211> crystallographic orientation, a direction previously associated with filamentous or dendritic growth [23]. In contrast, Li deposited in the presence of 0.5 wt.% POAmMe additive displays a markedly larger and more symmetric morphology (∼ 4 µm × 4 µm; Figure 3b), indicative of enhanced structural uniformity. Prior studies have shown that when the influence of the SEI on Li^+^ flux is minimized, lithium preferentially deposits along the <001> direction, forming faceted rhombic dodecahedral particles, which is consistent with the thermodynamically favored Wulff construction of body‐centered cubic (bcc) crystals [4]. Furthermore, larger nucleation domains are known to mitigate the evolution of high‐curvature dendritic protrusions during subsequent growth. The compositional and structural differences in the SEI layers are further corroborated, where distinct interfacial features are directly resolved at the atomic scale. As demonstrated in Figure 3c, the SEI film formed in blank ether electrolyte has a thickness of about 60–80 nm (marked in red), consisting of the external oxide layer (mainly Li_2_O and LiOH) and a complex internal layer, which consists of polymer layer (mainly ROLi (R = alkyl), formed by decomposition of DOL and DME), and fluoride layer dark area, mainly LiF generated by LiTFSI decomposition and α‐Li_3_N, LiN_x_O_y_ derived from reduction of LiNO_3_). The newly generated SEI film due to damage can be clearly observed externally (marked in green), which is mainly caused by the rupture of the SEI layer with low Young's modulus during the expansion process of Li metal. The structure of the SEI layer with POAmMe participation has undergone significant changes (Figure 3d and Figure S19). First, the average thickness of the SEI film with POAmMe participation is only 40 nm, and a dark passivation layer with a thickness of about 8 nm can be clearly seen at the lowest layer of the SEI film, proving the existence of Li_3_N and LiF. On top of it is a polyether layer with a thickness of about 20 nm and the participation of POAmMe. The clear interface between the two layers indicates that the SEI film has high integrity and has not suffered any damage during multiple cycles. Most importantly, on the top layer of the SEI film, a covering layer composed of polymer can be observed. When we zoom in and compare the top layers of the two SEI films, we can observe that, compared to the homogeneous layer formed in the blank ether electrolyte (Figure 3e), high‐density dendrites with a black stripe size of about 1 nm can be clearly observed in the SEI film with POAmMe participation. Figure 3f and Figure S20 demonstrate that the average distance between the stripes is around 1.7 nm, which matches the length of Amino in the POAmMe structure (1.5–1.6 nm). Based on previous research, it is determined that such regular and uniformly distributed patterns are composed of POAmMe and fused into the 3D molecular skeleton of the SEI film [24]. The EDS mapping of one single lithium nucleus covered by the POAmMe‐derived SEI reveals a uniform distribution of C, N, O, and Si signals on the surface, confirming the homogeneous incorporation of POSS cages within the SEI layer (Figure 3g and Figure S21).

To elucidate the interfacial electric field distribution and the mechanical characteristics of the SEI layer, Kelvin probe force microscopy (KPFM) and amplitude‐modulated atomic force microscopy (AM‐AFM) were employed. As shown in Figure 3h and Figure S22, the surface potential mapping of freshly deposited lithium metal reveals evident variations, particularly concentrated at regions with higher curvature, such as dendrite tips and edges. These sites exhibit stronger negative potential even under the same bias, indicating localized charge accumulation induced by rough surface morphology. Such inhomogeneous electric fields can lead to uneven Li^+^ flux and facilitate dendritic growth. To assess the mechanical stability of the SEI layer, we further performed nanomechanical phase mapping. As shown in Figure 3i, the SEI formed in the blank electrolyte exhibits a highly irregular topography, with vertical fluctuation up to 2.47 µm, reflecting severe surface instability. The phase contrast suggests increased viscoelastic heterogeneity near dendritic regions, due to mechanical failure and rupture of the SEI's inorganic framework. In contrast, with the introduction of POAmMe additive (Figure 3j), the surface becomes significantly smoother (vertical amplitude reduced to 836 nm), and the viscoelastic distribution is more uniform. The SEI in this case displays lower viscosity and enhanced stiffness, indicating improved mechanical robustness. This uniform and mechanically resilient SEI effectively suppress dendritic protrusion and maintain interfacial integrity during cycling.

### 2.4 Chemical Composition of SEI Layer

**FIGURE 4:**
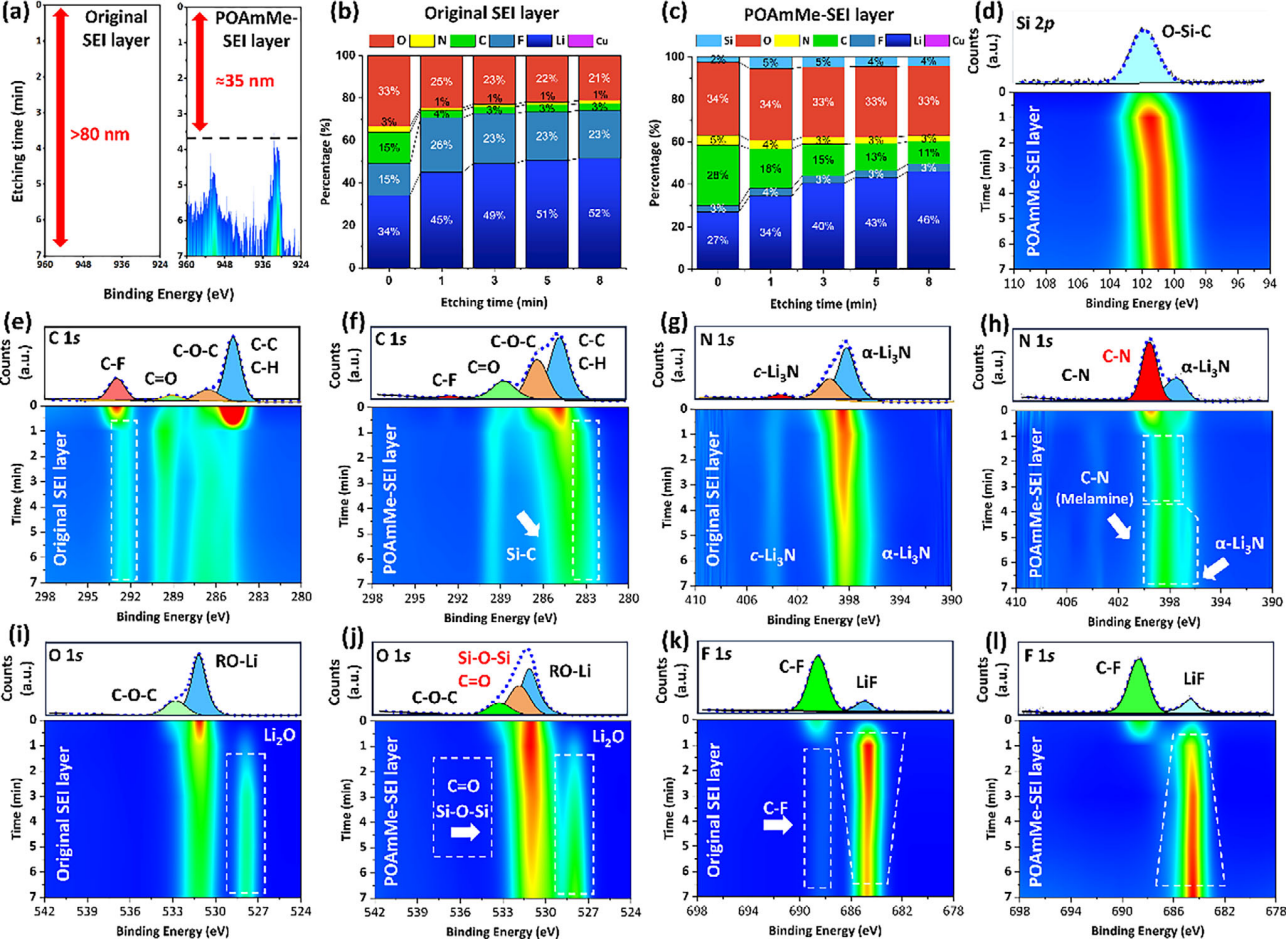
Chemical composition analysis of the SEI layer. Depth‐profiling XPS analysis of SEI layers formed on Cu foil with and without POAmMe additive. (a) Cu 2*p* signal comparison. (b,c) Elemental composition at various etching depths. High‐resolution XPS spectra of (d) Si 2*p*, (e,f) C 1*s*, (g,h) N 1*s*, (i,j) O 1*s*, and (k,l) F 1*s* for native and POAmMe‐modified SEI layers.

To investigate how POAmMe regulates the SEI composition, depth‐profiling XPS was conducted on Li deposits cycled for five cycles at 1 mA cm^−^^2^ and subsequently delithiated. Argon ion etching was used to analyze the SEI layer‐by‐layer (Figures S23–29). As shown in Figure 4a, no Cu signal was detected in the native SEI until the seventh minute of etching, corresponding to an estimated thickness of > 84 nm (assuming 0.2 nm s^−^^1^ etching rate). In contrast, Cu signals emerged after only 3 min of etching in the POAmMe‐modified SEI, indicating a thinner layer (∼ 40 nm), consistent with Cryo‐TEM results. Figure 4b,c reveals significant differences in chemical composition between two SEI layers. The native SEI contains high levels of Li (> 50%) and F (> 20%) throughout depth, reflecting continuous TFSI^−^ reduction and LiF accumulation. In comparison, the POAmMe‐containing SEI shows a marked increase in C (28%–11%) and N (5%–3%) content, alongside the appearance of Si from the POSS nanocages, indicating uniform incorporation of the additive. The F content is notably lower (< 5%), suggesting reduced electrolyte decomposition and SEI reformation cycles. Figure 4d shows the Si 2*p* signal at 101.8 eV (Si–C), persisting throughout the SEI, confirming the structural stability of the POSS cages. High‐resolution C 1*s* spectra (Figure 4e,f) further demonstrate diminished C–F peak intensity (∼293.1 eV) in the POAmMe‐integrated SEI, while the C–Si peak (284.4 eV) remains prominent, indicating effective fusion with the SEI matrix. Additionally, N 1*s* spectra (Figure 4g,h) exhibit a higher ratio of pyridinic N and ─NH─ (399.5 eV) in the modified SEI, attributed to melamine‐based secondary nodes and Amino acid linkages. The suppression of Li_3_N signals suggests improved electrochemical stability and solvation resistance imparted by the POAmMe architecture.

Figure 4i,j compares the differences in O signals between two SEI layers, and it can be seen that the presence of Si─O─S and C═O bonds greatly enhances the peak at 531.4–531.8 eV. Moreover, in the middle and interior depths, the Li_2_O (528.3 eV) content of the SEI film hybrid with POAmMe is slightly higher than that of the native SEI film, mainly due to the decomposition of high oxygen functional groups in POAmMe. Previous studies have shown that a moderate increase in Li_2_O can foster the Li ion exchange capacity, thereby enhancing the ion conductivity of the SEI layer [25]. In addition, the anti‐solvation effect of POAmMe is also reflected in the signal of F 1*s*. Figure 4k shows that the signal of C─F bond (688.7 eV) in the middle and lower parts of the native SEI layer is slightly higher than that of SEI fused with POAmMe (Figure 4l). Another distinct difference is that the signal intensity of LiF (688.7 eV) in the native SEI film gradually decreases from top to bottom (compared to C─F bonds), but the LiF in the SEI film fused with POAmMe gradually increases. This is because the native SEI film creates more opportunities for fresh Li to encounter TFSI^−^ during the rupture process, resulting in a higher proportion in the middle and upper parts. POAmMe can effectively enhance the toughness and self‐healing properties of SEI film, allowing most of the LiF to originate from the early stages of SEI film growth and be effectively blocked in the later stages.

To further elucidate the chemical robustness of POAmMe during prolonged electrochemical cycling, synchrotron‐based Si K‐edge X‐ray absorption near‐edge structure (XANES) spectroscopy was conducted before and after repeated Li plating/stripping processes (Figure S30). Notably, the Si K‐edge absorption onset of the cycled electrode exhibits a negligible shift compared to that of the pristine sample, indicating that the oxidation state of silicon remains essentially unchanged throughout cycling. Moreover, the nearly identical white‐line intensity and spectral profile suggest that the local coordination environment of Si atoms is well preserved. The absence of newly emerged features in the post‐edge region further confirms that no detectable chemical reconstruction or degradation of the Si─O─Si framework occurs during electrochemical operation. These results unambiguously demonstrate the exceptional chemical stability of the POSS‐based core in POAmMe under highly reductive lithium metal environments. Such structural integrity ensures that POAmMe functions as a durable and non‐sacrificial interfacial regulator, providing a stable molecular scaffold for dynamic ion transport modulation and long‐term stabilization of the lithium metal anode.

### 2.5 Theoretical Analysis and Finite Element Simulation

**FIGURE 5:**
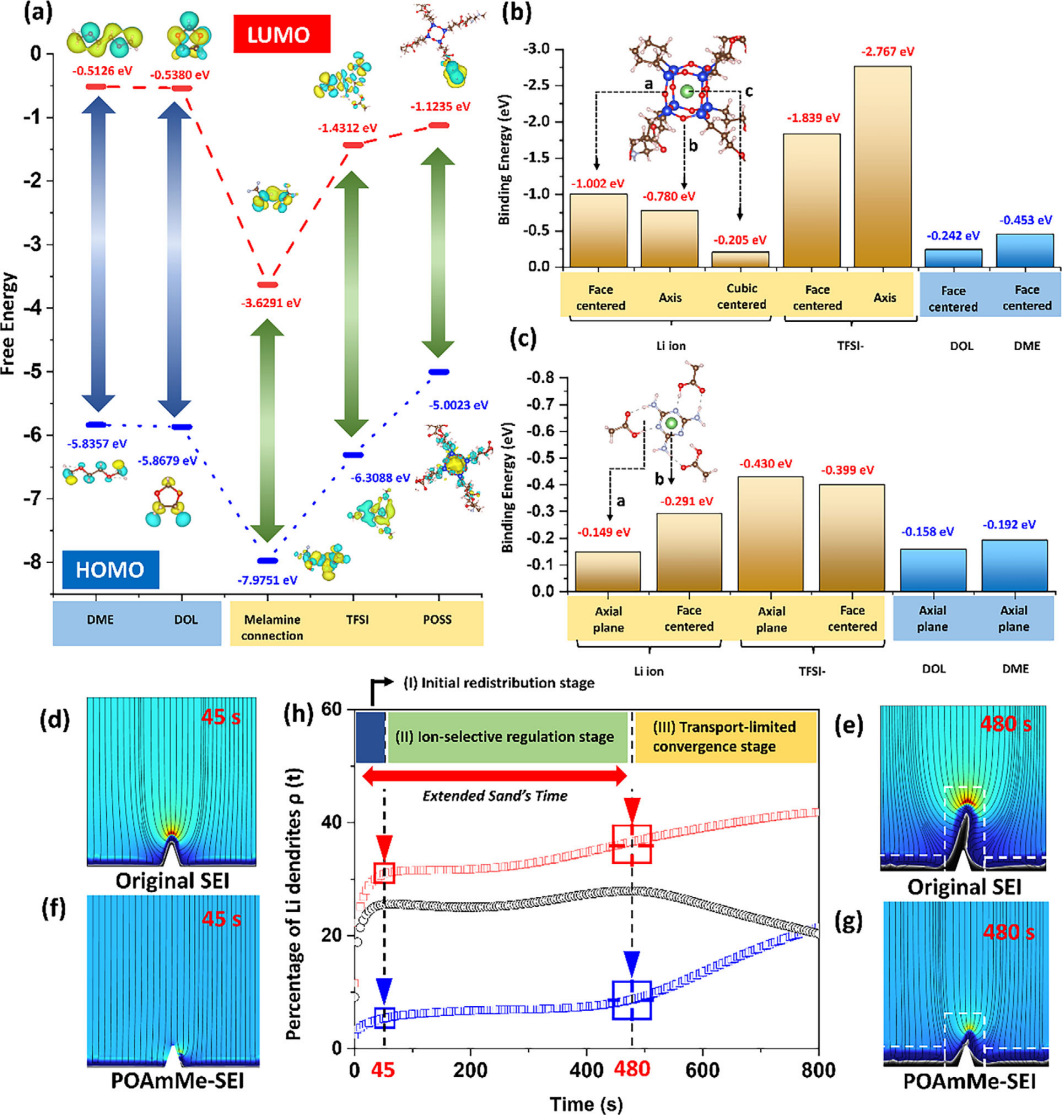
Theoretical analysis of electronic interactions and spatiotemporal dendrite suppression mechanism by POAmMe hybrid SEI. (a) HOMO and LUMO energy levels of electrolyte components (DME, DOL, TFSI^−^) and SEI constituents (POSS, melamine trimer), illustrating the electron donation/acceptance capability. (b,c) Binding energies of TFSI^−^ and Li^+^ with POSS and melamine trimer at different adsorption sites, revealing preferential ionic affinity at specific coordination modes. COMSOL simulation of electric field redistribution around growing Li dendrites at 45 and 480 s, comparing native SEI (d,e) and POAmMe‐modified SEI (f,g) conditions. (h) Temporal evolution of the dendritic Li area ratio during galvanostatic deposition, indicating three distinct growth regimes: (I) initial redistribution phase, (II) ion‐selective regulation phase, and (III) transport‐limited convergence phase. The first two stages define an extended Sand's time window, in which POAmMe guides Li^+^ flux away from dendrite tips, thereby suppressing vertical propagation.

To elucidate the interfacial stability and ion‐transport regulation mechanisms of the POAmMe polymer on lithium metal anodes, density functional theory (DFT) calculations were conducted on its two core structural units: the POSS nanocage and the hydrogen‐bonded melamine trimer (Figures S31–S39). As listed in Table S1, frontier molecular orbital (FMO) analysis revealed that the HOMO and LUMO energy levels of POSS are −5.00 and −1.12 eV, respectively, while those of the melamine trimer are −6.31 and −1.43 eV. These values are significantly lower than the Fermi level of metallic lithium (ca. −3.04 eV vs. Li/Li^+^), indicating the strong reductive stability of POAmMe (Figure 5a). This suggests that the polymer backbone is unlikely to undergo electron‐induced decomposition at the lithium interface, enabling it to function as an effective electronic barrier layer. To further assess the selective affinity of POAmMe toward electrolyte components, adsorption energy calculations were performed for Li^+^, TFSI^−^, and typical solvent molecules (DOL and DME) at different binding sites. As demonstrated in Figure 5b and Table S2, the TFSI^−^ anion exhibited strong binding on the Z‐axis site of the POSS cage with an adsorption energy of −2.767 eV, while its interaction with DOL (−0.242 eV) and DME (−0.453 eV) was much lower. Li^+^ showed moderate adsorption at the face‐centered and body‐centered sites of POSS (−0.780 and −1.002 eV), forming transient yet stable interactions that facilitate Li ion transport. Similar trends were observed for the melamine trimer (Figure 5c and Table S3), which displayed mild adsorption toward Li^+^ (−0.291 eV) and TFSI^−^ (−0.430 eV) but negligible affinity for solvent molecules. This distinct adsorption selectivity can be attributed to three key factors: (i) Li^+^ and TFSI^−^ are capable of engaging in electrostatic or Lewis acid–base interactions with the electron‐rich oxygen and nitrogen atoms in the polymer framework, whereas neutral solvent molecules lack such affinity; (ii) the semi‐enclosed geometry of the POSS cage imposes spatial hindrance that disfavors the penetration of bulky solvent species; and (iii) the densely hydrogen‐bonded melamine plane forms a supramolecular scaffold that further excludes solvent infiltration.

To elucidate the spatiotemporal influence of the POAmMe‐based SEI on lithium deposition behavior, we performed two‐dimensional finite element simulations using COMSOL Multiphysics (v6.2), incorporating ionic transport, interfacial kinetics, and dendrite geometry evolution (Figure S40 and Table S4). A growing lithium protrusion was modeled under galvanostatic conditions with either a native SEI (0% coverage) (Figure 5d,e) or an interfacial layer with 80% POAmMe coverage (Figure 5f,g). The dynamic evolution of the electric field and current density distributions reveals that the presence of POAmMe markedly redistributes the local field lines, effectively diverting Li^+^ flux from the high‐curvature dendrite tip toward its base. To quantitatively evaluate the extent of dendritic growth, a dendritic area ratio *ρ*(t) was defined based on the spatial distribution of deposited lithium obtained from two‐dimensional finite‐element simulations. In this framework, the total lithium deposition area, A_Li_(*t*) was calculated by integrating the Li‐occupied region over the entire computational domain Ω, as identified by a level‐set variable *ɸ*(r,t) exceeding a prescribed threshold. To further isolate dendritic lithium growth, a geometric height criterion along the growth direction was introduced. The dendritic area, A_d_(*t*), was then defined by subtracting the non‐dendritic basal deposition region from the total Li‐occupied area. Accordingly, the dendritic area ratio was expressed as

(1)

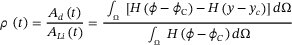

in which *H* (•) denotes the Heaviside step function. This dimensionless metric enables a quantitative and geometry‐independent description of dendritic growth evolution under different interfacial conditions.

As shown in Figure 5h, the time‐resolved evolution of this metric displays three distinct stages. In the initial redistribution stage (0–100 s), the ionic flux rapidly accumulates at the dendrite tip due to electric field concentration, and the regulatory role of POAmMe is not established yet. Once entering the ion‐selective regulation stage (100–480 s), the POAmMe layer dynamically guides Li^+^ toward the basal region, suppressing vertical dendrite propagation. At last, the system enters a transport‐limited convergence stage (after ∼ 480 s), where ionic depletion and interfacial saturation diminish the regulatory effect, and the dendritic area ratio between the two systems begins to converge.

Importantly, the first two stages together define an extended Sand's time regime, during which the interfacial ion concentration remains sufficiently high and the POAmMe‐modified SEI maintains effective flux regulation. This temporal window extends beyond the classical Sand's time—which traditionally marks the onset of ionic depletion—and serves as a functional lifetime for the suppression of dendritic growth. These findings provide direct evidence that molecularly engineered SEI layers such as POAmMe can spatiotemporally modulate electrochemical deposition processes, bridging nanoscale interface design with macroscopic morphological stability.

**FIGURE 6:**
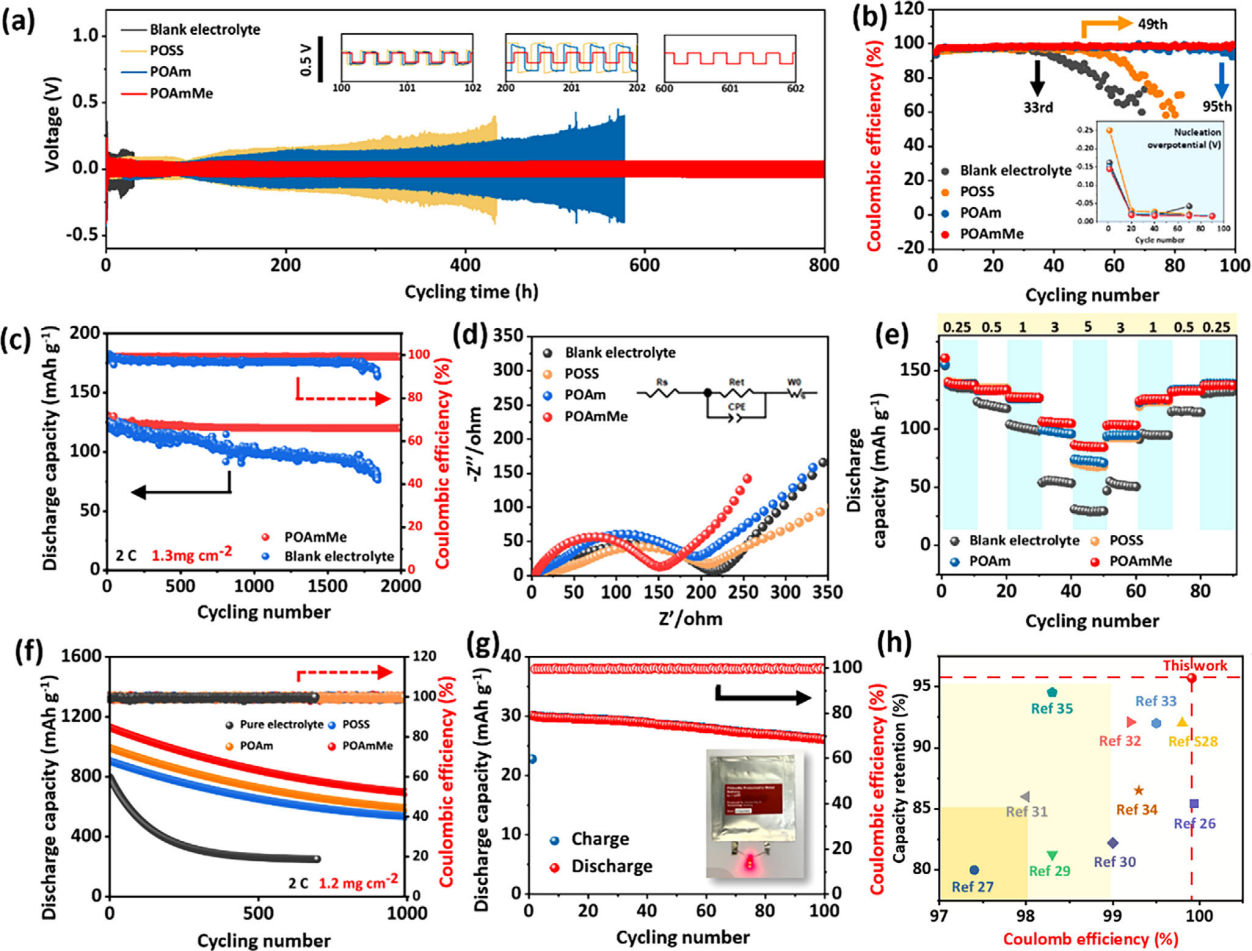
(a) Galvanostatic cycling of symmetric Li || Li cells using blank electrolyte and that containing POSS, POAm and POAmMe additives. The current density is fixed at 5 mA cm^−2^ with a plating/stripping capacity of 1 mAh cm^−2^. (b) Comparison of cycling performances of Li || Cu half cells with or without various additives. The amount of Li deposited in each cycle is 1 mAh cm^−2^ and the current density is 1 mA cm^−2^. The inset is the corresponding nucleation potential at the first 100 cycles of the Li plating/stripping processes. (c) Long term cycling stability of the Li || LTO full cells with or without various additives at a current density of 2C (1C = 175 mA g^−1^). (d) Comparison of EIS electrochemical impedance patterns of Li || LTO full cells using blank electrolyte and that with various additives after 500 cycles. (e) Rate capability from 0.25C to 5C of the Li || LTO cells with or without various additives in the electrolyte. (f) Comparison of galvanostatic current (2C) cycling performance between high load Li‐S cells using blank electrolyte and that with various additives. (g) Cycling performance of Li || LFP pouch full cell using POAmMe added electrolyte. (h) Comparison of electrochemical performance of the POAmMe additive with previously reported lithium metal anode stabilization strategies.

To evaluate the practical protective effect of the POSS‐based additives on lithium metal anodes, Li || Li symmetric cells were first assembled using electrolytes containing POSS, POAm, or POAmMe additives, and cycled at a high current density of 5 mA cm^−^^2^ with a fixed areal capacity of 1 mAh cm^−^^2^ (Figure 6a). The cell with the blank electrolyte suffered from a rapid voltage fluctuation and short‐circuited after only 32 cycles, indicative of uncontrolled dendritic lithium growth. Although the incorporation of POSS and POAm additives partially alleviated polarization, their overpotentials gradually increased with prolonged cycling and ultimately led to internal short circuits after 434 and 578 h, respectively. In sharp contrast, the POAmMe‐containing cell exhibited a relatively higher initial polarization (∼ 0.59 V), which rapidly decreased within the first 3 h and subsequently stabilized at an ultralow value of ∼ 0.12 V over an extended cycling duration exceeding 800 h. To further clarify the origin of the high initial polarization and its rapid decrease during early cycling, time‐resolved in situ EIS measurements were conducted on Li||Li symmetric cells with blank and POAmMe‐containing electrolytes under the same plating/stripping conditions (Figure S41). Compared with the blank cell, the POAmMe‐containing cell exhibits lower and more stable interfacial impedance during the initial cycling process. The impedance evolution indicates that the initially formed POAmMe‐rich interfacial layer undergoes electrochemically induced adsorption, reconstruction, and activation on the Li surface during repeated Li plating/stripping. This process progressively improves interfacial ion transport and matches well with the rapid decrease in polarization from ∼0.59 to ∼0.12 V within the first 3 h. Therefore, the high initial polarization is mainly attributed to the initial POAmMe adsorption/reconstruction on the Li surface and the formation of a transiently resistive POAmMe‐rich interphase, rather than solely to bulk disorder‐to‐order self‐assembly of the hydrogen‐bonded network. To further validate the dynamic structural evolution of the POAmMe framework under Li plating/stripping‐induced strain, in situ Raman spectroscopy was performed on Li||Li symmetric cells using the POAmMe‐containing electrolyte (Figure S42). During Li plating and stripping, the Raman bands associated with C═N, C═O, and N─H related vibrations showed reversible intensity and/or peak‐shape variations. During Li plating, the growth of deposited Li introduces local mechanical strain into the POAmMe‐derived interphase, which may induce partial dissociation or rearrangement of the melamine‐mediated hydrogen‐bonded junctions. During Li stripping, the removal of deposited Li releases the local strain, allowing these dynamic junctions to reassociate and partially recover. These reversible spectral variations support the proposed stress‐responsive dissociation/reassociation behavior of the POAmMe framework during repeated Li plating/stripping. Notably, when the current density was varied while maintaining an identical areal capacity (Figure S43), the POAmMe‐based symmetric cells consistently delivered lower polarization voltages across the entire tested current range compared with all counterparts. More importantly, when the current density was abruptly reduced from 5 to 0.2 mA cm^−^^2^, the polarization voltage of the POAmMe cell immediately recovered to its initial low level, providing compelling evidence for the rapid self‐repair capability of the POAmMe‐derived SEI. This behavior suggests that the interphase can dynamically accommodate stress accumulation under harsh plating conditions and spontaneously restore its ionic transport pathways once the stress is released.

The lithium reversibility was further quantified using Li || Cu half‐cells (Figure 6b). The cell with the blank electrolyte exhibited a quick decay in Coulombic efficiency after only 33 cycles, primarily due to continuous dead‐Li formation and irreversible lithium loss. In comparison, the POSS‐ and POAm‐based electrolytes delayed the onset of efficiency decay to the 49^th^ and 95^th^ cycles, respectively. Remarkably, the POAmMe‐containing cell maintained a high and stable average Coulombic efficiency of 98.4% for over 100 cycles. Analysis of the nucleation overpotential (inset of Figure 6b and Figure S44) reveals that the POSS additive induces the highest initial nucleation overpotential (−0.25 V), which can be attributed to its disordered interfacial arrangement that increases interfacial resistance. Although the initial nucleation overpotential of the POAmMe‐based cell (∼ −0.14 V) is comparable to that of the POAm and blank systems, its polarization continuously decreases with cycling and reaches an ultralow value of −0.015 V after 90 cycles, whereas the blank electrolyte exhibits a monotonic increase in polarization beyond 70 cycles. This evolution indicates the gradual formation of a stabilized, low‐resistance, and mechanically adaptive interphase enabled by the POAmMe framework.

To further assess the interfacial stabilization effect under practical conditions, full cells employing Li_4_Ti_5_O_12_ (LTO) cathodes were assembled. Cyclic voltammetry measurements (Figure S45) show that the POAmMe‐based full cells display the smallest separation between oxidation and reduction peaks at various scan rates, reflecting reduced polarization and enhanced lithium‐ion transport kinetics. Consistently, during galvanostatic cycling at a high rate of 2C (Figure 6c), the cell with the blank electrolyte exhibits rapid capacity decay after 550 cycles and eventually short‐circuits at the 1841^st^ cycle. In contrast, the POAmMe‐based full cell delivers highly stable cycling performance, maintaining a capacity of 120 mAh g^−^^1^ even after 2000 cycles. Electrochemical impedance spectroscopy (Figure 6d and Figure S46) further confirms that the POAmMe‐derived interphase effectively suppresses the growth of interfacial resistance upon long‐term cycling, corroborating its anti‐fatigue and self‐healing characteristics under repeated lithium plating/stripping. Rate performance measurements shown in Figure 6e and Figure S47 demonstrate that the incorporation of the POAmMe additive consistently enhances the cycling stability of Li || LTO full cells across a wide range of current densities. Notably, the performance improvement becomes more pronounced at higher current densities, where the POAmMe‐based cells maintain lower polarization and more stable capacity retention. These results further indicate that the POAmMe‐derived interphase can effectively stabilize the lithium metal anode under fast lithium plating/stripping conditions, thereby enabling improved rate capability and cycling robustness in full cell configurations. In fact, as shown in Figure S48, the protective effect of POAmMe becomes more pronounced during long‐term cycling tests at current densities of 3C and 5C.

To evaluate the protective capability of the POAmMe additive in high‐energy‐density cell systems, lithium–sulfur (Li–S) cells were employed as a more demanding test platform (Figure S49). As shown in Figure 6f, when the sulfur loading reaches 1.2 mg cm^−^^2^ and the cell is cycled at a rate of 2C, the Li–S cell incorporating the POAmMe additive delivers a stable discharge capacity of 695 mAh g^−^^1^ after 1000 cycles. This value is markedly higher than those of the cells using POSS (590 mAh g^−^^1^) and POAm (535 mAh g^−^^1^) additives. In contrast, the cell with the blank electrolyte exhibits the onset of micro‐short‐circuit behavior after 698 cycles, indicating severe interfacial instability of the lithium metal anode. The interfacial protection ability of POAmMe becomes even more pronounced under higher current densities. As shown in Figure S50, at a high rate of 5C, the POAmMe‐based Li–S battery retains a capacity of 441 mAh g^−^^1^ after 1000 cycles, representing a 42.7% improvement compared with the counterpart employing only the POSS additive. These results indicate that the self‐assembled POAmMe interphase not only suppresses lithium dendrite growth but also effectively mitigates the detrimental impact of polysulfide species on the lithium metal surface, thereby enhancing interfacial stability in chemically complex environments.

To demonstrate the universality and practical applicability of the POAmMe additive, it was subsequently introduced into ester‐based electrolytes and evaluated in LiFePO_4_ (LFP)||Li pouch cells. As shown in Figure 6g, when 0.5 wt.% POAmMe is added to the ester electrolyte and applied in an LFP full cell with a cathode loading of 180 mg, the cell cycled at 1C (178 mA g^−^^1^) maintains a capacity retention of 87% after 100 cycles, with the capacity decreasing from 30 to 26.1 mAh. To further validate the reproducibility of this result, two additional independently assembled LFP||Li pouch cells were tested under the same conditions. As shown in Figure S51, the repeated pouch cells exhibit consistent cycling behavior and stable Coulombic efficiency, confirming the reliable effectiveness of POAmMe in practical pouch‐cell configurations. These results demonstrate that the protective effect of POAmMe on lithium metal anodes is not limited to ether‐based electrolytes and remains effective in ester‐based systems and large‐capacity cells. Benefiting from the high energy density, the assembled LFP||Li pouch cell can power a smartphone device (Figure S52), highlighting the practical feasibility of the POAmMe additive in realistic applications. Finally, as summarized in Figure 6h, a comparison with previously reported representative strategies shows that the POAmMe additive delivers state‐of‐the‐art performance in both Coulombic efficiency and capacity retention, underscoring its effectiveness in stabilizing lithium metal anodes across diverse energy storage chemistries [26, 27, 28, 29, 30, 31, 32, 33, 34, 35].

## 3 Conclusion

In summary, we report a “Geomag”‐inspired, POSS‐based molecular additive that enables the construction of an antifatigue and self‐healing solid electrolyte interphase (SEI) for lithium metal anodes. Distinct from conventional SEI strategies relying on static rigidity or inorganic reinforcement, the POAmMe framework introduces a dynamically reconfigurable interfacial architecture that can adapt to mechanical stress during repeated lithium plating and stripping. The hierarchically assembled structure, composed of star‐shaped POSS primary nodes and reversibly connected melamine‐based secondary junctions, allows the SEI to temporarily decouple under localized stress and subsequently restore its integrity through spontaneous reconnection. This dynamic “disconnect–reconnect” behavior effectively regulates local Li^+^ flux and electric‐field distribution, thereby suppressing dendrite nucleation and mitigating interfacial fatigue accumulation. As a result, POAmMe‐protected lithium metal anodes exhibit reduced interfacial thickness, suppressed impedance growth, and markedly enhanced electrochemical durability. The optimized interphase enables stable cycling for over 800 h in symmetric cells at 5 mA cm^−^^2^, maintains a high average Coulombic efficiency of 98.4% in Li || Cu cells, and supports ultralong cycling in Li || Li_4_Ti_5_O_12_ full cells, delivering a stable capacity of 120 mAh g^−^^1^ after 2000 cycles at 2C. Beyond the specific chemistry demonstrated here, this work establishes a general molecular‐level design paradigm for constructing antifatigue SEI layers, providing new insights into stabilizing lithium metal anodes under high‐rate and long‐term operating conditions.

## Author Contributions

**Zairan Xiao**: data curation. **Yu Liu**: data curation, methodology. **Zixia Lin**: methodology. **Tianyi Wang**: writing – review and editing, writing – original draft, methodology, validation, resources, data curation, conceptualization. **Di He**: software. **Yaojie Lei**: investigation, supervision. **Hui Chong**: investigation, supervision. **Wei Gu**: data curation, writing – review and editing, methodology, software, validation. **Chengyin Wang**: investigation, supervision, project administration, resources, funding acquisition. **Jiabao Li**: investigation, supervision. **Xiaobo Zheng**: investigation, supervision. **Bing Sun**: investigation, supervision, project administration, resources. **Tao Huang**: investigation, supervision. **Guoxiu Wang**: investigation, supervision, project administration, resources.

## Funding

C. W. gratefully acknowledges the financial support by the National Natural Science Foundation of China (Grant No. 22472145), Postgraduate Research & Practice Innovation Program of Jiangsu Province (Yangzhou University) (KYCX24_3729).

## Conflicts of Interest

The authors declare no conflicts of interest.

## Supporting information




**Supporting File**: advs76111‐sup‐0001‐SuppMat.docx.

## Data Availability

Research data are not shared.

## References

[1] M. Zheng , Y. You , and J. Lu , “Understanding Materials Failure Mechanisms for the Optimization of Lithium‐Ion Battery Recycling,” Nature Reviews Materials 10 (2025): 355–368, 10.1038/s41578-025-00783-5.

[2] J. Jang , C. Wang , G. Kang , et al., “Miniature Li^+^ Solvation by Symmetric Molecular Design for Practical and Safe Li‐Metal Batteries,” Nature Energy 10 (2025): 502–512, 10.1038/s41560-025-01733-9.

[3] K. Yan , J. Wang , S. Zhao , et al., “Temperature‐Dependent Nucleation and Growth of Dendrite‐Free Lithium Metal Anodes,” Angewandte Chemie International Edition 58 (2019): 11364–11368, 10.1002/anie.201905251. 31148342

[4] X. Yuan , B. Liu , M. Mecklenburg , and Y. Li , “Ultrafast Deposition of Faceted Lithium Polyhedra by Outpacing SEI Formation,” Nature 620 (2023): 86–91, 10.1038/s41586-023-06235-w. 37532813

[5] D. Lin , Y. Liu , and Y. Cui , “Reviving the Lithium Metal Anode for High‐Energy Batteries,” Nature Nanotechnology 12 (2017): 194–206, 10.1038/nnano.2017.16. 28265117

[6] H. Liang , H. Xie , H. Yu , et al., “High‐Voltage and Wide‐Temperature Lithium Metal Batteries with High‐Safety Enabled by Non‐Flammable Electrolytes,” Materials Science and Engineering: R: Reports 169 (2026): 101177, 10.1016/j.mser.2026.101177.

[7] Q. Li , G. Liu , Y. Chen , et al., “Electrolyte Solvent‐Ion Configuration Deciphering Lithium Plating/Stripping Chemistry for High‐Performance Lithium Metal Battery,” Advanced Functional Materials 35 (2025): 2420327, 10.1002/adfm.202420327.

[8] S. Li , H. Xie , P. Kumar , et al., “Ether‐Oxygen Groups Modified Carboxylic Ester Enabling High‐Voltage Lithium Metal Batteries,” Angewandte Chemie International Edition 64 (2025): 202504490. 10.1002/anie.202504490 40407398

[9] Y. Chen , Z. Ma , Y. Wang , et al., “Trace Ethylene Carbonate‐Mediated Low‐Concentration Ether‐Based Electrolytes for High‐Voltage Lithium Metal Batteries,” Energy & Environmental Science 17 (2024): 5613–5626, 10.1039/D4EE01831A.

[10] C. Chen , J. Zhang , B. Hu , Q. Liang , and X. Xiong , “Dynamic Gel as Artificial Interphase Layer for Ultrahigh‐Rate and Large‐Capacity Lithium Metal Anode,” Nature Communications 14 (2023): 4018, 10.1038/s41467-023-39636-6. PMC10328938 37419911

[11] S. Chen , G. Wu , H. Jiang , et al., “External Li Supply Reshapes Li Deficiency and Lifetime Limit of Batteries,” Nature 638 (2025): 676–683, 10.1038/s41586-024-08465-y. 39939772

[12] T. Wang , Y. Li , J. Zhang , et al., “Immunizing Lithium Metal Anodes against Dendrite Growth Using Protein Molecules to Achieve High Energy Batteries,” Nature Communications 11 (2020): 5429, 10.1038/s41467-020-19246-2. PMC7591880 33110084

[13] J. Lu , T. Wang , J. Yang , et al., “Multifunctional Self‐Assembled Bio‐Interfacial Layers for High‐Performance Zinc Metal Anodes,” Angewandte Chemie International Edition 63 (2024): 202409838. 10.1002/anie.202409838 39058295

[14] M. Huang , C.‐H. Hsu , J. Wang , et al., “Selective Assemblies of Giant Tetrahedra via Precisely Controlled Positional Interactions,” Science 348 (2015): 424–428, 10.1126/science.aaa2421. 25908818

[15] M. Wang , Y. Song , S. Zhang , et al., “Programmable Two‐dimensional Nanocrystals Assembled from POSS‐containing Peptoids as Efficient Artificial Light‐harvesting Systems,” Science Advances 7 (2021): abg1448, 10.1126/sciadv.abg1448. PMC8121420 33990330

[16] J. Jin , Y. Zhu , Z. Zhang , and W. Zhang , “Enhancing the Efficacy of Photodynamic Therapy through a Porphyrin/POSS Alternating Copolymer,” Angewandte Chemie International Edition 57 (2018): 16354–16358, 10.1002/anie.201808811. 30318668

[17] K. Tanaka , F. Ishiguro , and Y. Chujo , “POSS Ionic Liquid,” Journal of the American Chemical Society 132 (2010): 17649–17651, 10.1021/ja105631j. 21105729

[18] Y.‐T. Weng , H.‐W. Liu , A. Pei , et al., “An Ultrathin Ionomer Interphase for High Efficiency Lithium Anode in Carbonate Based Electrolyte,” Nature Communications 10 (2019): 5824, 10.1038/s41467-019-13783-1. PMC6925282 31862992

[19] H. Zhao , W. She , D. Shi , W. Wu , Q.‐C. Zhang , and R. K. Y. Li , “Polyurethane/POSS Nanocomposites for Superior Hydrophobicity and High Ductility,” Composites Part B: Engineering 177 (2019): 107441, 10.1016/j.compositesb.2019.107441.

[20] A. J. Waddon and E. B. Coughlin , “Crystal Structure of Polyhedral Oligomeric Silsequioxane (POSS) Nano‐materials: a Study by X‐ray Diffraction and Electron Microscopy,” Chemistry of Materials 15 (2003): 4555–4561, 10.1021/cm034308b.

[21] Y. Nan , S. Li , Y. Shi , S. Yang , and B. Li , “Gradient‐Distributed Nucleation Seeds on Conductive Host for a Dendrite‐Free and High‐Rate Lithium Metal Anode,” Small 15 (2019): 1903520, 10.1002/smll.201903520. 31529764

[22] Y. Li , Y. Li , A. Pei , et al., “Atomic Structure of Sensitive Battery Materials and Interfaces Revealed by Cryo–electron Microscopy,” Science 358 (2017): 506–510, 10.1126/science.aam6014. 29074771

[23] Z. Sun , Y. Wang , S. Shen , et al., “Directing (110) Oriented Lithium Deposition through High‐Flux Solid Electrolyte Interphase for Dendrite‐Free Lithium Metal Batteries,” Angewandte Chemie International Edition 62 (2023): 202309622, 10.1002/anie.202309622. 37606605

[24] X. Wang , P. Gao , Y. Yang , H. Guo , and D. Wu , “Dynamic and Programmable Morphology and Size Evolution via a Living Hierarchical Self‐Assembly Strategy,” Nature Communications 9 (2018): 2772, 10.1038/s41467-018-05142-3. PMC6050331 30018381

[25] S. Zhang , Y. Li , L. Bannenberg , M. Liu , S. Ganapathy , and M. Wagemaker , “The Lasting Impact of Formation Cycling on the Li‐ion Kinetics between SEI and the Li‐Metal Anode and Its Correlation with Efficiency,” Science Advances 10 (2024): adj8889, 10.1126/sciadv.adj8889. PMC10793961 38232156

[26] S. Fang , F. Wu , S. Zhao , et al., “Adaptive Multi‐Site Gradient Adsorption of Siloxane‐Based Protective Layers Enable High Performance Lithium‐Metal Batteries,” Advanced Energy Materials 13 (2023): 2302577, 10.1002/aenm.202302577.

[27] J. Wu , T. Zhou , B. Zhong , Q. Wang , W. Liu , and H. Zhou , “Designing Anion‐Derived Solid Electrolyte Interphase in a Siloxane‐Based Electrolyte for Lithium‐Metal Batteries,” ACS Applied Materials & Interfaces 14 (2022): 27873–27881, 10.1021/acsami.2c05098. 35671243

[28] Y. Wang , Y. Ni , S. Xu , et al., “Fully Methylated Siloxane‐Based Electrolyte for Practical Lithium Metal Batteries,” Journal of the American Chemical Society 147 (2025): 10772–10783, 10.1021/jacs.5c02140. 40085124

[29] T. Yim , K. Kim , M. Q. Hassig , et al., “Lithium‐Nitrate‐Containing Gel Polymer Electrolyte for Carbonate‐Based Anode‐Free Lithium Metal Batteries,” ACS Applied Materials & Interfaces 17 (2025): 37851–37862, 10.1021/acsami.5c03252. 40530945

[30] Y. Wang , M. Zhou , J. Yang , et al., “Polyhedral Oligomeric Silsesquioxane Modified Polyolefin Separator for Advanced Lithium–Metal Batteries,” ACS Applied Energy Materials 7 (2024): 6400–6407, 10.1021/acsaem.4c01105.

[31] X. Wei , J. Zhou , X. Wang , et al., “POSS Based Poly (Zwitterionic Liquids) Electrolytes with 3D Crosslinked Networks for Lithium Metal Batteries,” Chemical Engineering Journal 498 (2024): 155614, 10.1016/j.cej.2024.155614.

[32] J. Ma , X. Ma , H. Zhang , et al., “In‐Situ Generation of Poly(Ionic Liquid) Flexible Quasi‐Solid Electrolyte Supported by Polyhedral Oligomeric Silsesquioxane/Polyvinylidene Fluoride Electrospun Membrane for Lithium Metal Battery,” Journal of Membrane Science 659 (2022): 120811, 10.1016/j.memsci.2022.120811.

[33] C.‐C. Chang , M.‐H. Shen , Y.‐S. Hsu , H. Teng , and J.‐S. Jan , “In Situ Formed Composite Polymer Electrolytes Based on Anion‐Trapping Boron Moiety and Polyhedral Oligomeric Silsesquioxane for High Performance Lithium Metal Batteries,” Small Science 4 (2024): 2400183, 10.1002/smsc.202400183. 40212261 PMC11934992

[34] P. Liu , J. Zhang , L. Zhong , et al., “Interphase Building of Organic–Inorganic Hybrid Polymer Solid Electrolyte with Uniform Intermolecular Li^+^ Path for Stable Lithium Metal Batteries,” Small 17 (2021): 2102454, 10.1002/smll.202102454. 34514698

[35] C. Gao , X. Hu , Y. Huang , and X. Ma , “In‐Situ Solidification POSS‐Crosslinked Polymer Electrolytes in Multiscale Nanocellulose Membranes for High‐Performance All‐Solid‐State Lithium Batteries,” Composites Part B: Engineering 305 (2025): 112736, 10.1016/j.compositesb.2025.112736.

