# Supplementary material for: Geomag Mimetic Dynamically Reconfigurable POSS Framework Enables Antifatigue Solid Electrolyte Interphases for Lithium Metal Anodes
Source: Adv Sci (Weinh). 2026 Jul 21:e76111. Online ahead of print. doi: 10.1002/advs.76111 (PMC13387874; doi:10.1002/advs.76111)
Supplement: Supplementary file 1 — Supporting File: advs76111‐sup‐0001‐SuppMat.docx. [file ADVS-9999-e76111-s001.docx]

Supporting Information

**Geomag mimetic dynamically reconfigurable POSS framework enables antifatigue solid electrolyte interphases for lithium metal anodes**

*Tianyi Wang ^1, 2^* **, Wei Gu ^1^, Yu Liu ^1^, Di He ^1^, Zairan Xiao ^1^, Zixia Lin ^4^, Tao Huang ^2^, Jiabao Li ^1^, Hui Chong ^1^, Yaojie Lei ^2^,* *Xiaobo Zheng ^2^, Bing Sun ^2^* **, Guoxiu Wang ^2^*, *Chengyin Wang* *^1, 3^* *

^1^ School of Chemistry and Materials, Yangzhou University, 180 Si-Wang-Ting Road, Yangzhou, 225002, Jiangsu Province, P. R. China.

^2^ Centre for Clean Energy Technology, School of Mathematical and Physical Sciences, Faculty of Science, University of Technology Sydney, NSW 2007, Australia.

^3^ Jiangsu Provincial Key Laboratory of Green & Functional Materials and Environmental Chemistry, Yangzhou, 225002, Jiangsu Province, P. R. China.

^4^ Test Center of Yangzhou University, 48 Wen-Hui-Dong Road, 225002, Jiangsu Province, P. R. China

E-mail: Tianyi Wang ([Tianyi.wang@uts.edu.au](mailto:Tianyi.wang@uts.edu.au)); Bing Sun ([Bing.sun@uts.edu.au](mailto:Bing.sun@uts.edu.au)); Chengyin Wang ([wangcy@yzu.edu.cn](mailto:wangcy@yzu.edu.cn))

1. **Experimental Section**

**1.1 Materials**

Polypropylene (PP) separators (Celgard 2400, thickness ≈ 25 μm) were purchased from Celgard (USA). CR2032 coin cell cases were obtained from Kelude Experimental Equipment Technology Co., Ltd. (China). Lithium titanate (Li₄Ti₅O₁₂, battery grade) was supplied by BTR New Energy Materials Inc. (China). Poly(vinylidene fluoride) (PVDF, 99.9%), copper foil (99.9%), conductive carbon black (Super P, battery grade), and carboxymethyl cellulose (CMC) were purchased from Shenzhen Kejing Zhida Technology Co., Ltd. (China). Lithium metal foil was obtained from Kelude Experimental Equipment Technology Co., Ltd. (China).

1,3-Dioxolane (DOL, 99.9%) and lithium bis(trifluoromethanesulfonyl)imide (LiTFSI, 99.9%) were purchased from Dodo Reagent (China). A commercial electrolyte consisting of 1 M LiTFSI dissolved in a DOL/DME mixture (v/v = 1:1) with 1 wt% LiNO₃ was also obtained from Dodo Reagent (China). Acrylate-functionalized polyhedral oligomeric silsesquioxane (POSS, 90%) was purchased from Shanghai Macklin Biochemical Co., Ltd. (China).

*1.1.1 Synthesis of POSS-Amino (POAm)*

Acrylate-functionalized polyhedral oligomeric silsesquioxane (POSS, 300 mg) and 11-aminoundecanoic acid (Amino, 731 mg) were dissolved in anhydrous dimethyl sulfoxide (DMSO) in a 10 mL round-bottom flask. The reaction mixture was heated to 65 °C under magnetic stirring and maintained at this temperature for 7 days to allow aza-Michael addition between the acrylate groups of POSS and the primary amino group of Amino.

After completion of the reaction, the resulting solution was transferred into a pretreated dialysis membrane (molecular weight cutoff, MWCO = 2500 Da; length = 34 cm) using a disposable pipette, and the membrane was tightly sealed with clamps. The dialysis bag was subsequently immersed in deionized water to remove excess unreacted Amino. During dialysis, the deionized water was replaced every 4 h for a total of eight cycles, followed by an additional dialysis period of 12 h.

After dialysis, the retained product was collected into centrifuge tubes and dried in a vacuum oven until a constant weight was achieved. The obtained solid was gently ground to yield POAm as a white powder (756 mg, isolated yield ≈ 85%).

*1.1.2 Synthesis of POSS-Amino-Melamine (POAmMe)*

POAm (500 mg, ca. 3.0 mmol) and melamine (57 mg, 8.0 mmol) were dispersed in tetrahydrofuran (THF) and stirred at room temperature for 24 h to promote the formation of a hydrogen-bond-mediated supramolecular network between the amino groups of POAm and melamine molecules.

After stirring, the resulting mixture was dried in a vacuum oven at 60 ℃ to completely remove the solvent. The obtained solid was subsequently redispersed and dissolved in deionized water preheated to 50 ℃, transferred into centrifuge tubes, and centrifuged. After centrifugation, the supernatant was discarded, and the washing–centrifugation process was repeated eight times to remove unassembled POAm and free melamine.

Finally, the collected white precipitate was dried under vacuum to a constant weight, yielding POAmMe as a white powder.

**1.2 Cell assembling and test**

*1.2.1 Reparation of LTO Cathodes*

Lithium titanate (Li₄Ti₅O₁₂, LTO) cathodes were prepared using a mass ratio of 8:1:1 for the active material, conductive agent, and binder. Specifically, LTO, conductive carbon black (Super P), and carboxymethyl cellulose (CMC) were thoroughly mixed and ground. An appropriate amount of deionized water was then added to form a homogeneous slurry, which was subsequently cast onto copper foil current collectors using a doctor blade. The coated electrodes were dried in a vacuum oven at 100 ℃ for 12 h to remove residual solvent. After drying, the electrodes were punched into circular disks with a diameter of 12 mm for coin cell assembly.

*1.2.2 Cell Assembly*

All coin cells were assembled in an argon-filled glovebox with water and oxygen levels maintained below 0.1 ppm. For each cell, 20 μL of electrolyte was added. Lithium metal foils with a diameter of 15 mm were used as the counter or reference electrodes. The diameters of the LTO cathodes and copper foils were 12 mm, while the separators had a diameter of 19 mm. Three types of cells were assembled as described below.

*1.2.3 Li || Li Symmetric Cells*

The positive casing was first placed on the assembly platform, followed by placing a lithium metal foil inside the cathode casing while ensuring no contact with the casing wall. A sufficient amount of electrolyte was added onto the lithium surface. Subsequently, a separator was placed on top of the lithium foil and wetted with electrolyte. Another lithium foil was then placed on the separator. Spacers, springs, and the anode casing were assembled sequentially, and the cell was finally sealed using a crimping machine.

*1.2.4 Li || Cu Half Cells*

The cathode casing was first placed on the assembly platform, and a copper foil was inserted without contacting the casing wall, followed by the addition of electrolyte onto the copper surface. A separator was then placed on the copper foil and wetted with electrolyte, after which a lithium metal foil was placed on top of the separator. The spacer, spring, and anode casing were assembled sequentially, and the cell was sealed by crimping.

*1.2.5 Li || LTO Full Cells*

The cathode casing was first placed on the assembly platform, and the LTO cathode was inserted while maintaining separation from the casing wall. Electrolyte was added onto the cathode surface, followed by placing a separator and adding electrolyte to ensure full wetting. A lithium metal foil was then placed on the separator. Finally, the spacer, spring, and anode casing were assembled sequentially, and the cell was sealed using a crimping machine.

*1.2.6 Preparation of Electrolytes*

A predetermined amount of POAmMe additive powder (purity ≈ 80%) was added to a commercial electrolyte consisting of 1 M LiTFSI dissolved in a DOL/DME mixture (v/v = 1:1) with 1 wt% LiNO₃, resulting in a final additive concentration of ~ 1 wt%. All procedures were carried out in an argon-filled glovebox.

The obtained electrolyte was sealed and subjected to ultrasonic dispersion for 1 h (15 min per cycle, four cycles in total) to ensure homogeneous dispersion of the additive. Subsequently, the electrolyte was magnetically stirred for 12 h inside the glovebox and then allowed to stand for an additional 12 h until a uniform and clear solution was obtained. The resulting electrolyte was used directly for subsequent electrochemical measurements.

All electrolyte preparation processes were performed under a high-purity argon atmosphere, with both moisture and oxygen levels maintained below 0.1 ppm.

**1.3 Characterization**

Scanning electron microscopy (SEM) images were obtained by the Zeiss-Supra 55 microscope at an acceleration voltage of 5-10 kV. Transmission electron microscopy (TEM) and Energy Dispersive Spectrometer (EDS) elemental mapping scans were recorded using Tecnai G2 F30 S-TWIN at an acceleration voltage of 300 kV, which was equipped with a Gatan 626 cryo-holder. The Powder X-ray diffraction (PXRD) patterns were performed by Bruker AXS D8 advance with Cu Kα radiation of 40 kV (λ=1.5418 Å). Fourier transform infrared (FT-IR) spectra were obtained with 670-IR + 610-IR from Varian, USA. Top-mounted diffuse reflectance attachment (diamond attachment). The spectral range is 4000-400 cm^-1^. Raman spectra were obtained via INVIA REFLEX (Renishaw), in the range of 150 - 4000 cm^-1^. Inductively coupled plasma optical emission spectroscopy (ICP-OES) was obtained using an Optima 7300 DV from PerkinElmer, USA. The wavelength range was 163 – 782 nm, and the precision was RSD ≤ 1 %. XPS analysis was carried out using a Thermo Scientific ESCALAB 250Xi X-ray photoelectron spectrometer with Al Kα radiation of 1486.6 eV as the excitation source, the survey thickness is 2-3 nm. The reference for calibration is the peak of C 1*s* at 284.8 eV. The element analyzer adopts the Vario EL cube made by Elementar Company in Germany, and its accuracy ranges are C, H, N, S < 0.1 % and O < 0.2 %.

**1.4 Electrochemical Measurements**

*1.4.1 Cyclic Voltammetry*

Cyclic voltammetry (CV) measurements were conducted to investigate the redox behavior and reversibility of the electrochemical reactions. CV tests were performed on Li || LTO full cells within a voltage window of 1.0–2.5 V (vs. Li/Li⁺) at scan rates ranging from 0.1 to 1.0 mV s⁻¹.

*1.4.2 Electrochemical Impedance Spectroscopy (EIS)*

EIS measurements were carried out to analyze the interfacial resistance and electrochemical kinetics of the cells, particularly the properties of the solid electrolyte interphase (SEI). The EIS measurements were performed over a frequency range from 10⁻² to 10⁵ Hz. The amplitude of the alternating current perturbation was set to 5 mV for Li || LTO full cells and 10 mV for Li || Li symmetric cells.

*1.4.3 Lithium-Ion Transference Number Measurements*

The lithium-ion transference number was evaluated using the chronoamperometry (CA) method in Li || Li symmetric cells. A constant potential of 10 mV was applied, and the current response was recorded over a duration of 3000 s to assess lithium-ion transport behavior at the electrode/electrolyte interface.

*1.4.4 Galvanostatic Charge–Discharge Tests*

Galvanostatic charge–discharge tests were conducted using a NEWARE battery testing system at a constant temperature of 25 °C. Li || Li symmetric cells, Li || Cu half cells, and Li || LTO full cells were systematically investigated. Li || Li symmetric cells were cycled under constant current conditions to monitor polarization voltage evolution and cycling stability. Li || Cu half cells were subjected to lithium plating/stripping tests under constant current, during which the charge and discharge capacities were recorded to evaluate the deposition and stripping behavior of lithium metal on copper substrates. Li || LTO full cells were tested within a voltage range of 1.0–2.5 V to evaluate capacity retention and cycling stability, thereby demonstrating the practical applicability of lithium metal anodes.

**1.5 Calculation method**

Colinear spin-polarized density functional theory (DFT) calculations ^[1, 2]^ were carried out in the Vienna ab initio simulation package (VASP) based on the plane-wave basis sets with the projector augmented-wave method.^[3, 4]^ The exchange-correlation potential was treated by using a generalized gradient approximation (GGA) with the Perdew-Burke-Ernzerhof (PBE) parametrization.^[5]^ The energy cutoff was set to be 500 eV. The Brillouin-zone integration was sampled with a Γ-centered Monkhorst-Pack mesh of 1 × 1 × 1 of POSS and POSS@*X* (*X* = Li, DME, DOL, TFSI) structures by VASPKIT.^[6, 7]^ The structures were fully relaxed until the maximum force on each atom was less than 0.02 eV/Å, and the energy convergent standard was 10^-5^ eV. The van der Waals correction of Grimme’s DFT-D3 model was also adopted.^[8]^

**1.6 Simulation methods**

The Nernst–Planck model, comprising current conservation and species conservation, provides a quantitative description of ionic transport and chemical reactions in solution. Level set method is used to describe dendrite distribution. The Nernst-Planck model solves the current density $\boldsymbol{i}$, concentration $c$, and electric potential $\phi$. Inside the computational domain:

$\boldsymbol{i}=FD\cdot\boldsymbol{\nabla}c-\frac{Dc}{RT}\boldsymbol{\nabla}\phi$ (2)

$\boldsymbol{\nabla}\cdot\boldsymbol{i}=0$ (3)

Deposition gives a convection velocity to level set equation, which solves the proportion $\phi$ of lithium metal:

$\frac{\partial\phi}{\partial t}+\boldsymbol{V}_{n}\boldsymbol{\nabla}\phi={\{V_{nx},V_{ny}\}}_{max}\boldsymbol{\nabla}\cdot(\epsilon\boldsymbol{\nabla}\phi-\phi(1-\phi)\boldsymbol{n}_{\nabla\phi})$ (4)

$\boldsymbol{V}_{n}=\left( V_{nx},V_{ny} \right)=\frac{MF}{2\rho}i \boldsymbol{n}^{T}\boldsymbol{n}$ (5)

Where $M$, $\rho$ are respectively molar mass and mass density of deposition metal, $\epsilon$ takes 1/4 of the maximum mesh size, $n_{\nabla\phi}$ is the unit vector in $\nabla\phi$ direction.

The computational domain is 1um × 1um. The electrode surfaces has a constant averaged current density $i_{0}=100A/m^{2}$, and divided into two part: covered electrode surface and custom electrode surface. We use no-flux boundary condition to simulate covered electrode surface, which satisfies

$\boldsymbol{n}\cdot\boldsymbol{i}=0, \boldsymbol{n}\cdot\left( \epsilon\boldsymbol{\nabla}\phi-\phi\left( 1-\phi\right)n_{\nabla\phi} \right)=0$ (6)

On custom electrode surface,

$i_{0}\int_{\mathrm{electrode}} ⅆS=\int_{\mathrm{electrode}} \boldsymbol{i}\cdot\boldsymbol{n}ⅆS, \phi=1$ (7)

Where $\boldsymbol{i}$ satisfies Bulter-Volmer model.

The mechanical behavior described above was analyzed via finite element simulations implemented in COMSOL Multiphysics V6.2. Validated unstructured meshes were adopted.

1. **List of Supporting Figures and Tables**


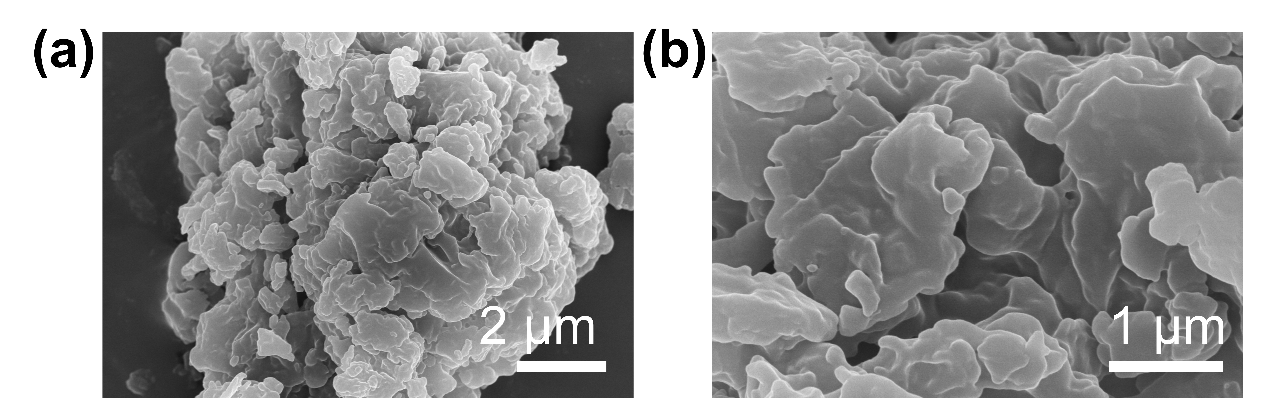


**Figure S1**. SEM images of POAmMe polymetric clots.


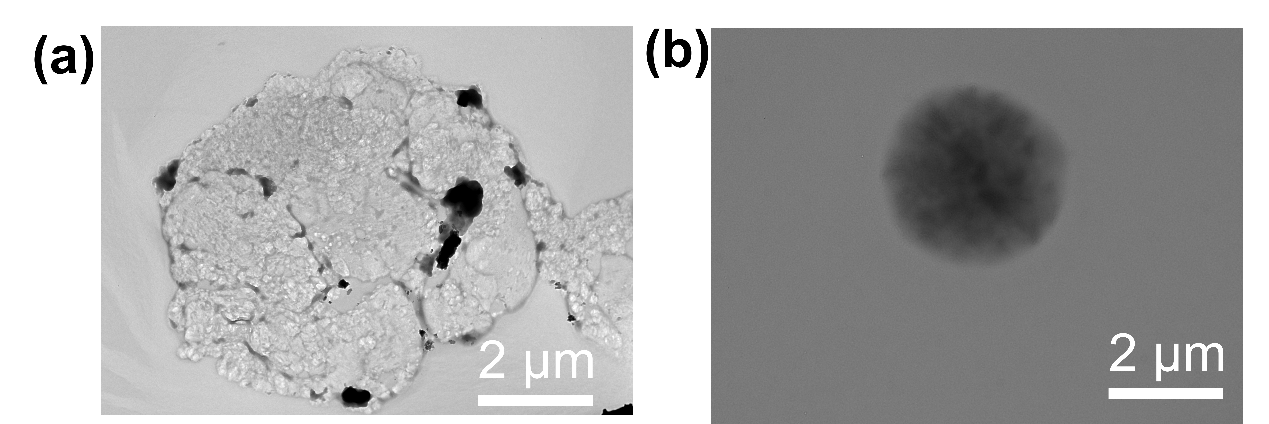


**Figure S2**. TEM images of POAmMe polymetric clots.


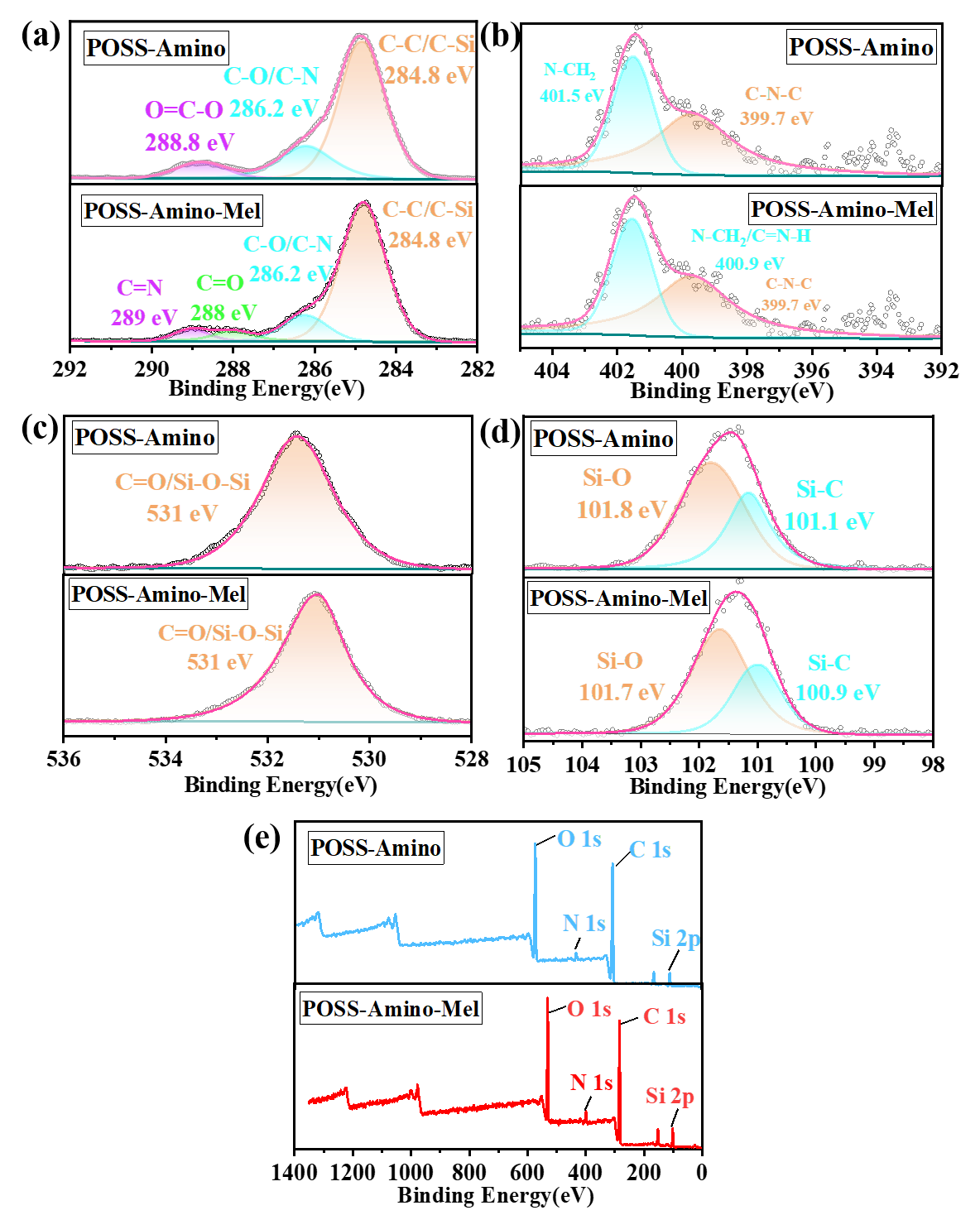


**Figure S3**. XPS spectra of POAm and POAmMe: (a) C 1*s*; (b) N 1*s*; (c) O 1*s*; (d) Si 2*p*; and (e) survey spectrum.


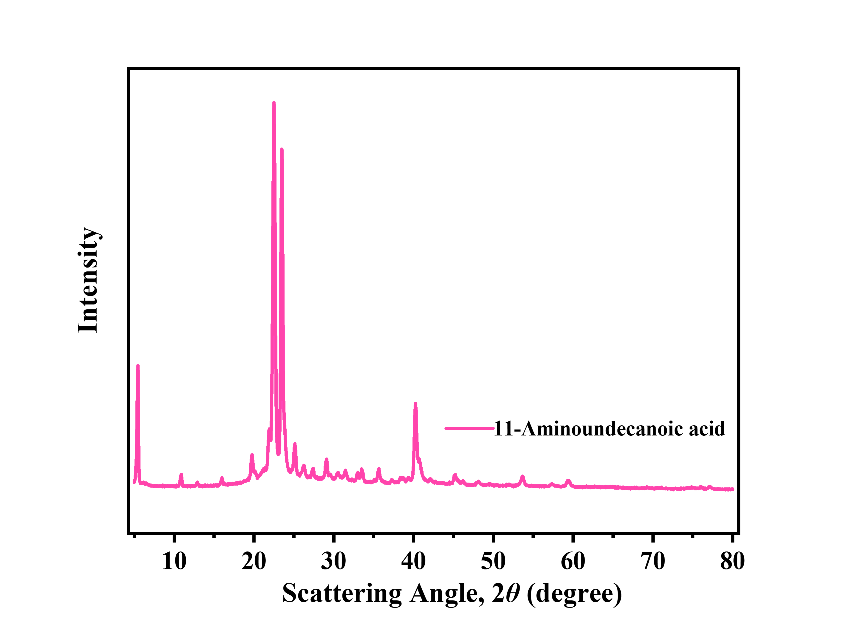


**Figure S4**. The XRD pattern of the Amino.

####
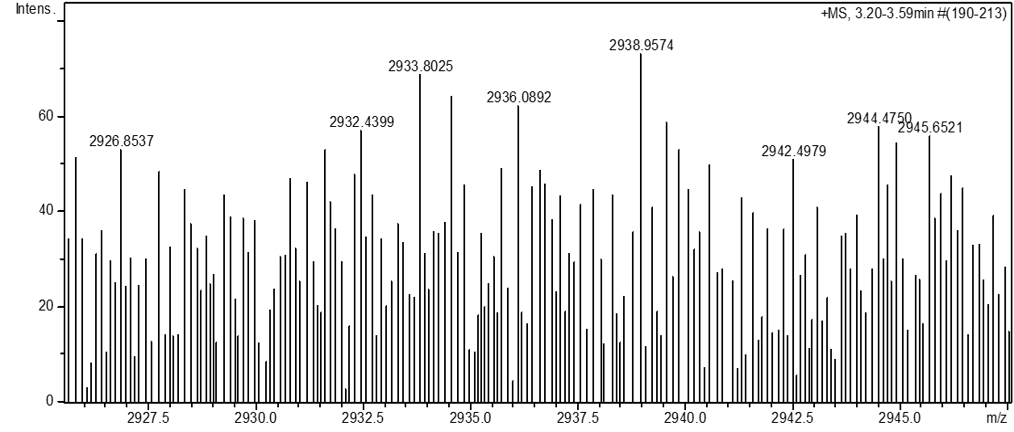


**Figure S5**. Ultra-High-Resolution Mass Spectrum of POAm.


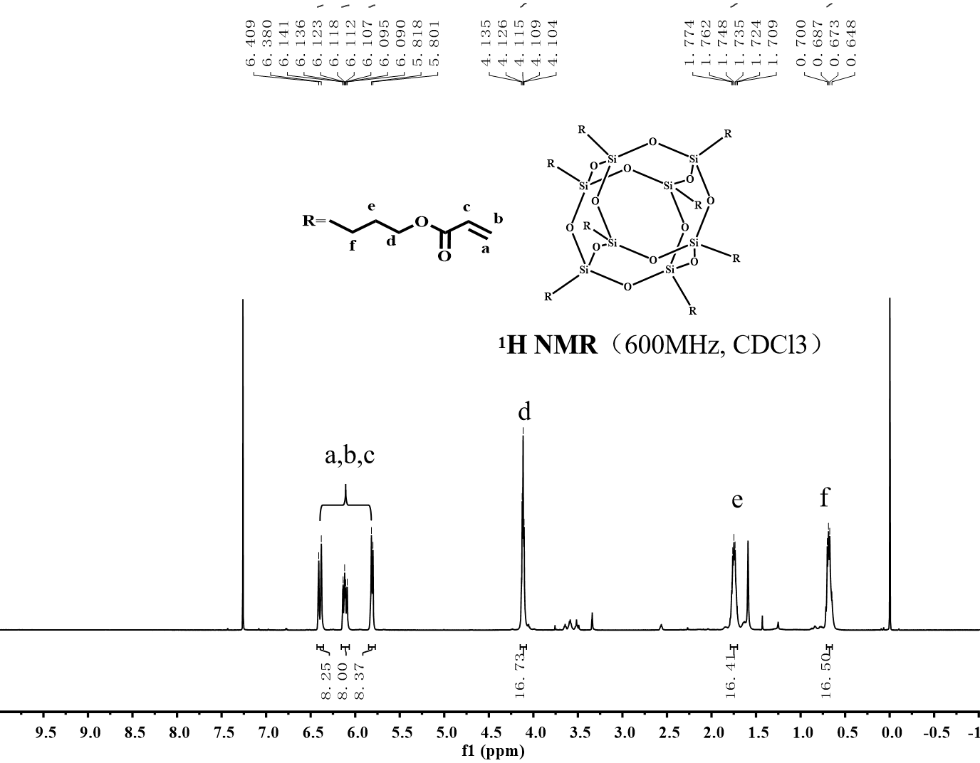


**Figure S6**. ^1^H NMR Spectrum and peak assignment of Acrylo POSS molecular cages. The characteristic acrylate vinyl proton signals at δ ≈ 5.8–6.5 ppm are assigned to the terminal C=C–H protons of the acrylate groups, confirming the reactive acrylate termini on the POSS cage. The aliphatic signals originate from the organic substituents connected to the POSS core, and the sharp signal at δ = 7.26 ppm is assigned to residual CHCl₃. The vinyl proton region was used as the reference to evaluate acrylate consumption after aza-Michael addition.


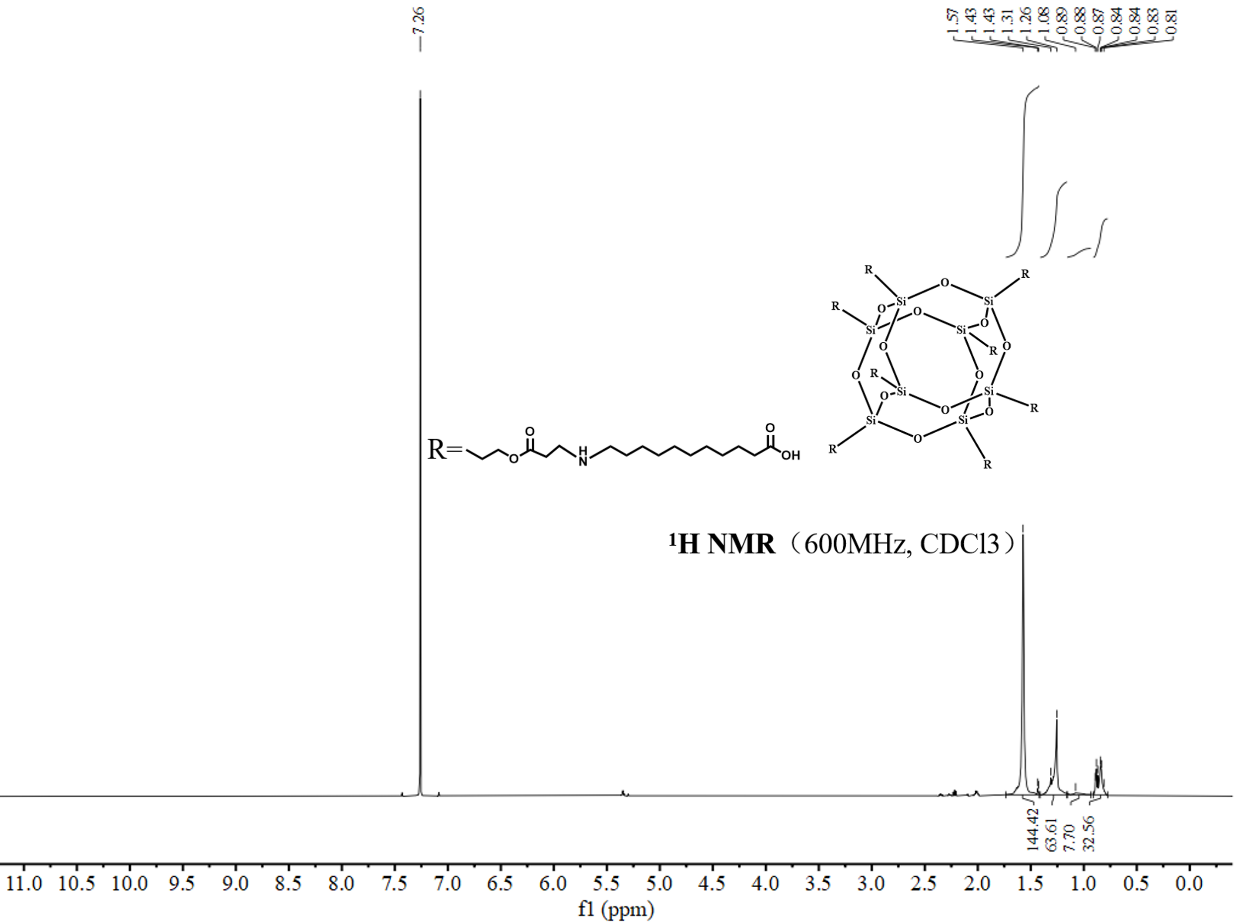


**Figure S7**. ^1^H NMR Spectrum of POAm. The sharp signal at δ = 7.26 ppm is assigned to residual CHCl₃. The characteristic acrylate vinyl proton signals expected at δ ≈ 5.8–6.5 ppm are negligible after the aza-Michael addition, indicating effective consumption of terminal acrylate groups. The aliphatic proton signals at δ = 1.57, 1.51, 1.43, 1.31, 1.25, 1.08, and 0.90–0.81 ppm are assigned to the methylene protons of the grafted Amino chains and POSS-connected organic substituents. Based on the negligible residual vinyl proton signals, the substitution degree of POAm is estimated to be close to complete, corresponding to a high average number of Amino-derived substituents per POSS cage. The average substitution degree was estimated to be close to eight Amino-derived substituents per POSS cage.


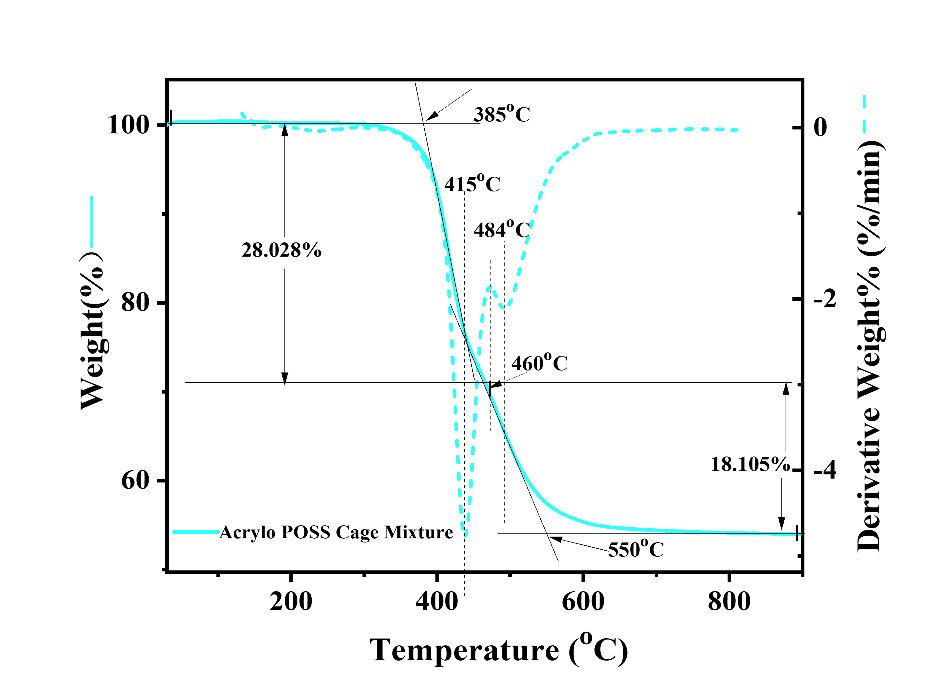


**Figure S8**. TGA/DTG curves of POSS at a heating rate of 10 °C min^-1^ under a nitrogen atmosphere.


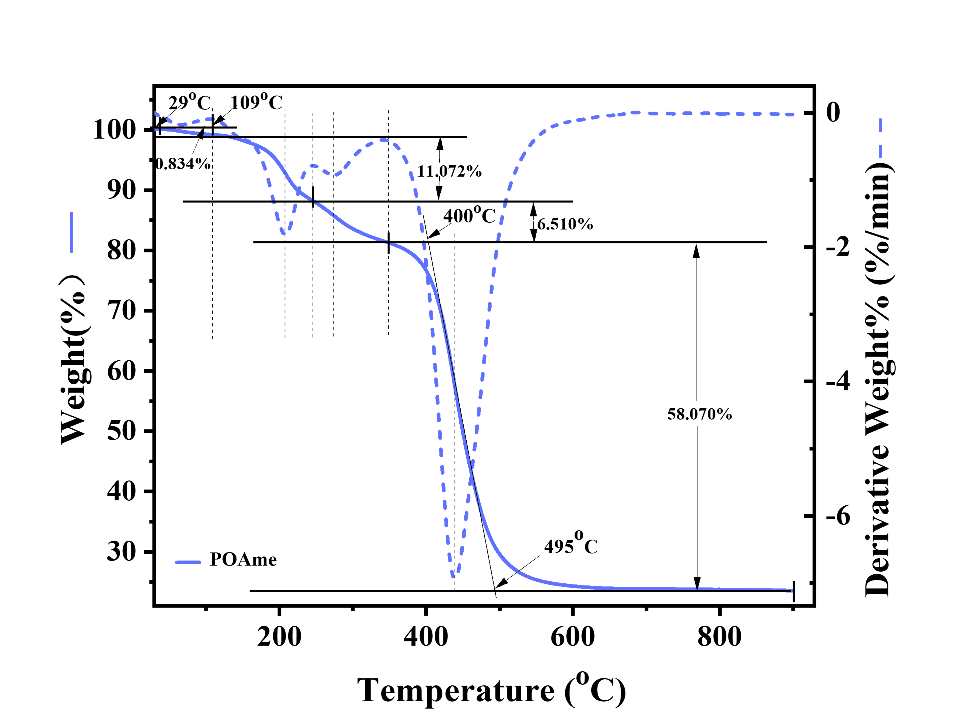


**Figure S9**. TGA/DTG curves of POAm at a heating rate of 10 °C min^-1^ under a nitrogen atmosphere.


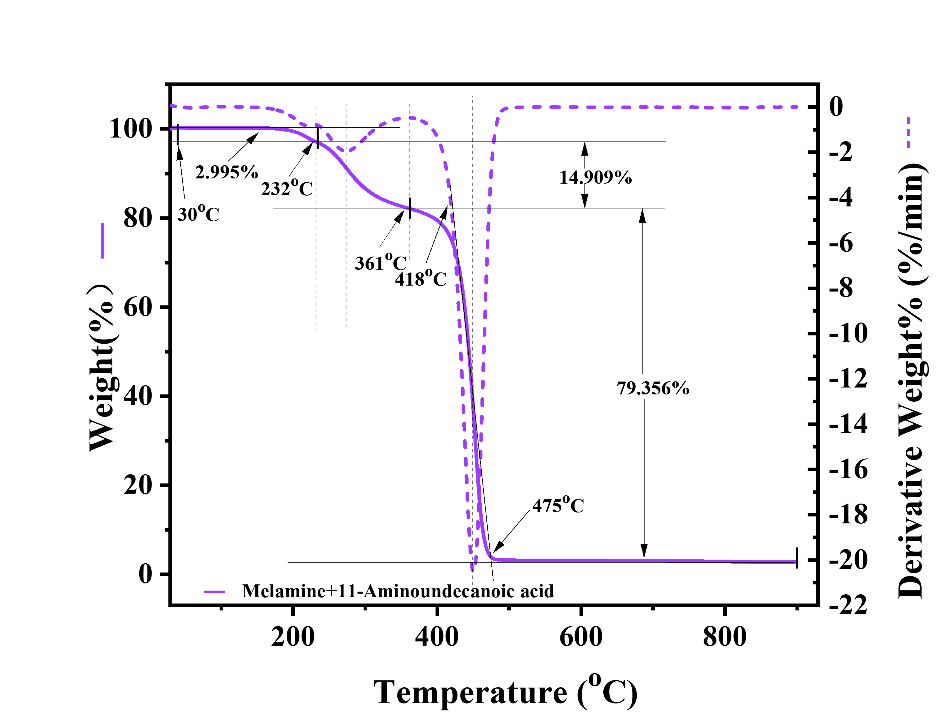


**Figure S10**. TGA/DTG curves of Amino-Melamine at a heating rate of 10 °C min^-1^ under a nitrogen atmosphere.


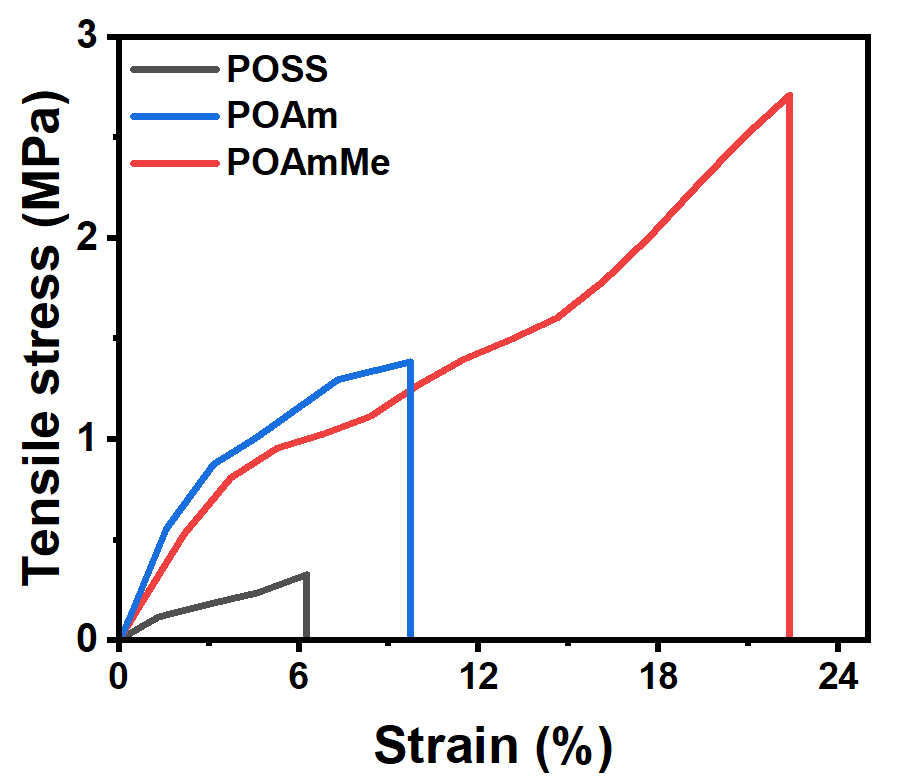


**Figure S11.** Stress–strain curves of POSS, POAm, and POAmMe, showing the enhanced mechanical robustness and stretchability of POAmMe.


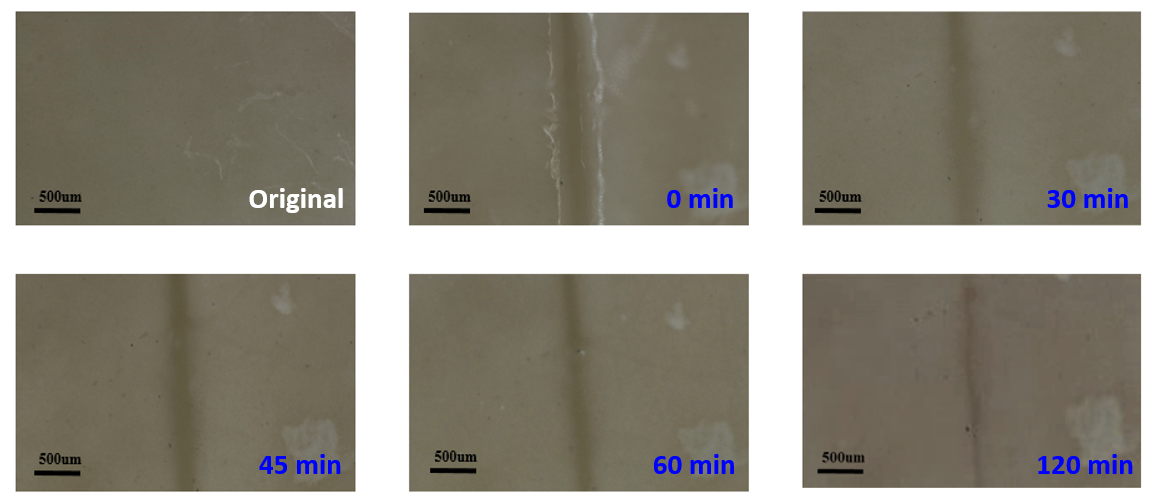


**Figure S12**. Time-dependent photographs of wound healing at the incision site treated with POAmMe, which was immersed in electrolyte, during the healing experiment at room temperature (25 ^o^C).


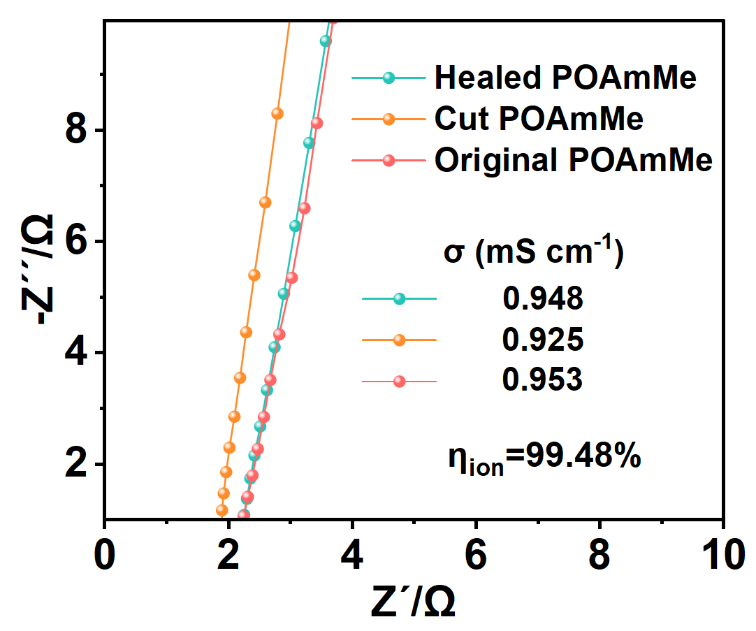


**Figure S13.** Ionic-conductivity recovery of POAmMe after cutting and healing. Nyquist plots of original, cut, and healed POAmMe samples measured using stainless-steel blocking electrodes in ether electrolyte at room temperature.


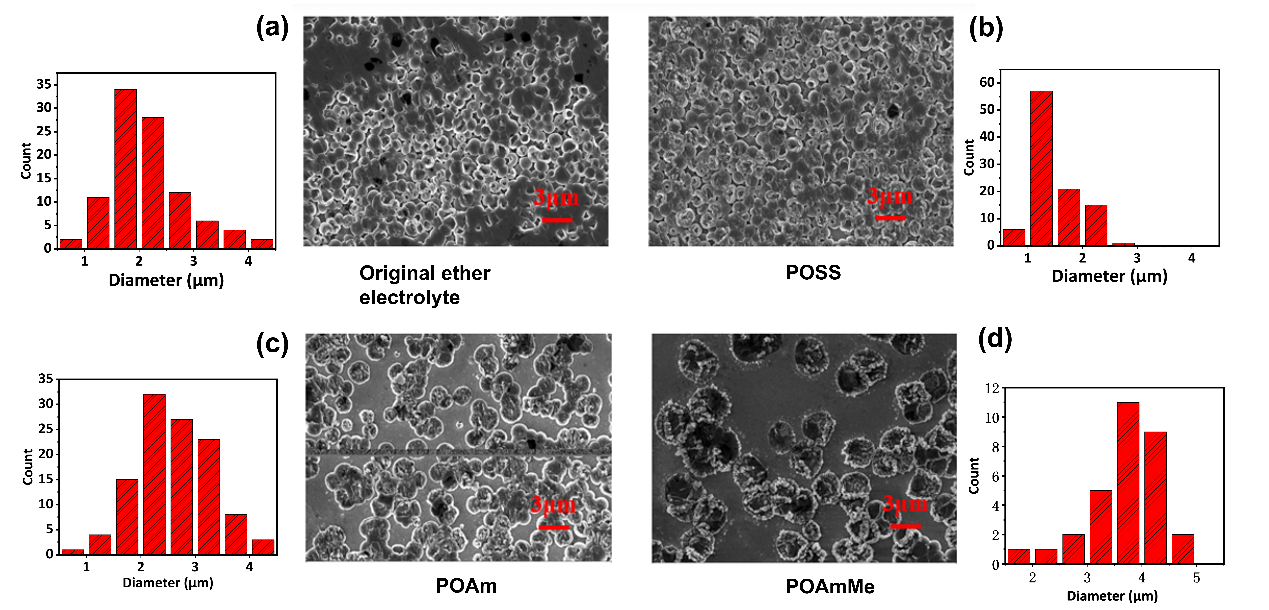


**Figure S14**. SEM images demonstrating distribution of Li buds after Cu foil deposited for 1 mAh cm^-1^ at 1mA cm^-1^ using (a) blank ether electrolyte, and that with (b) POSS, (c) POAm and (d) POAmMe additives.


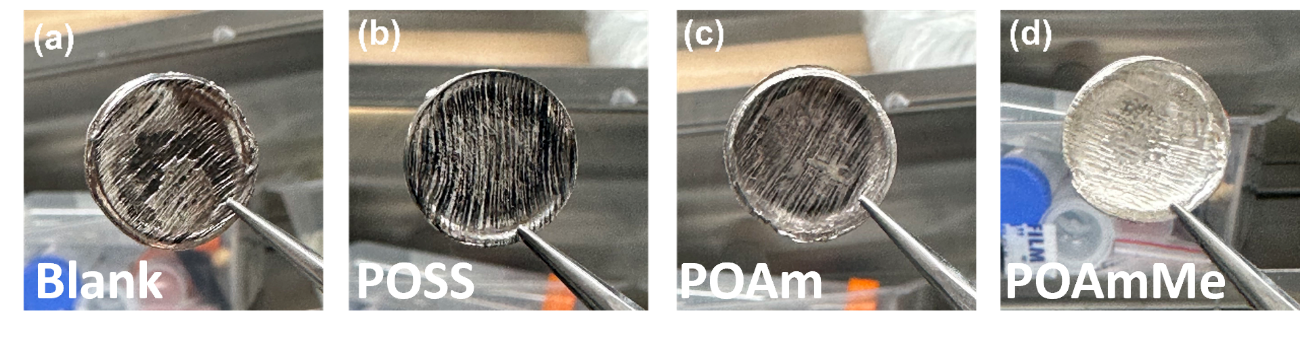


**Figure S15**. Digital photos of retrieved Li metal anodes after cycled for 50 cycles at 3 mA cm^-2^ for 1 mAh cm^-2^ using pristine ether electrolyte and that with POSS, POAm and POAmMe.


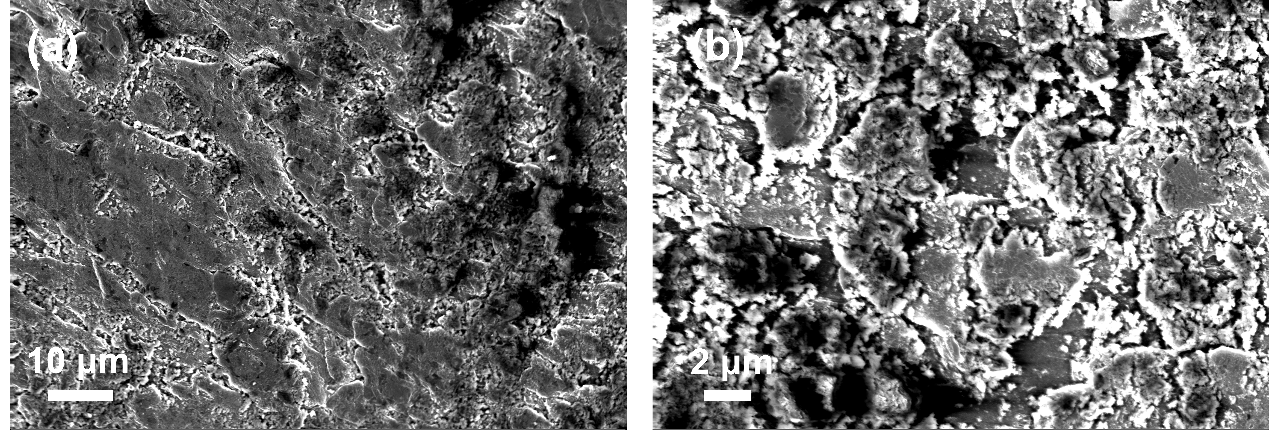


**Figure S16**. SEM images of Li metal anode deposited with POSS additive.


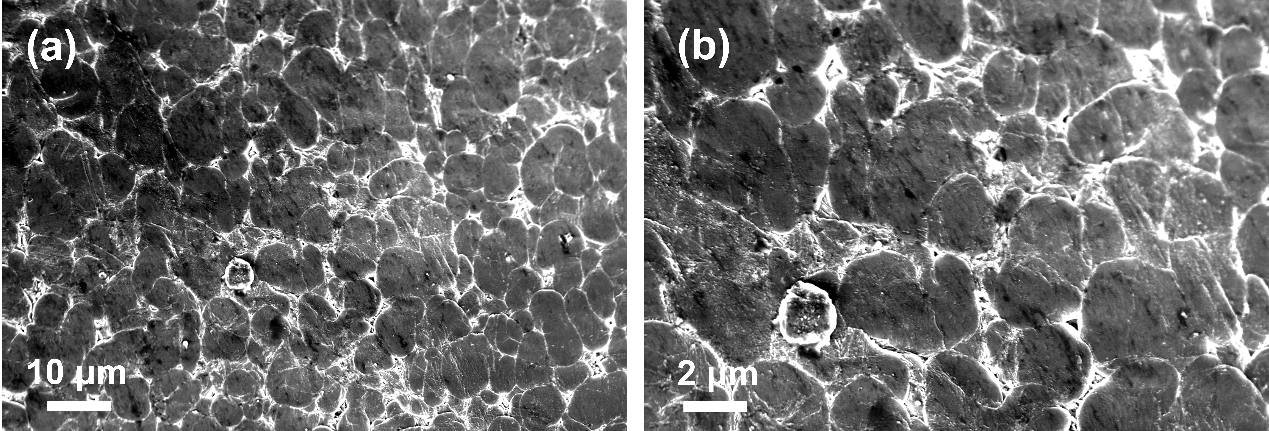


**Figure S17**. SEM images of Li metal anode deposited with the POAm additive.


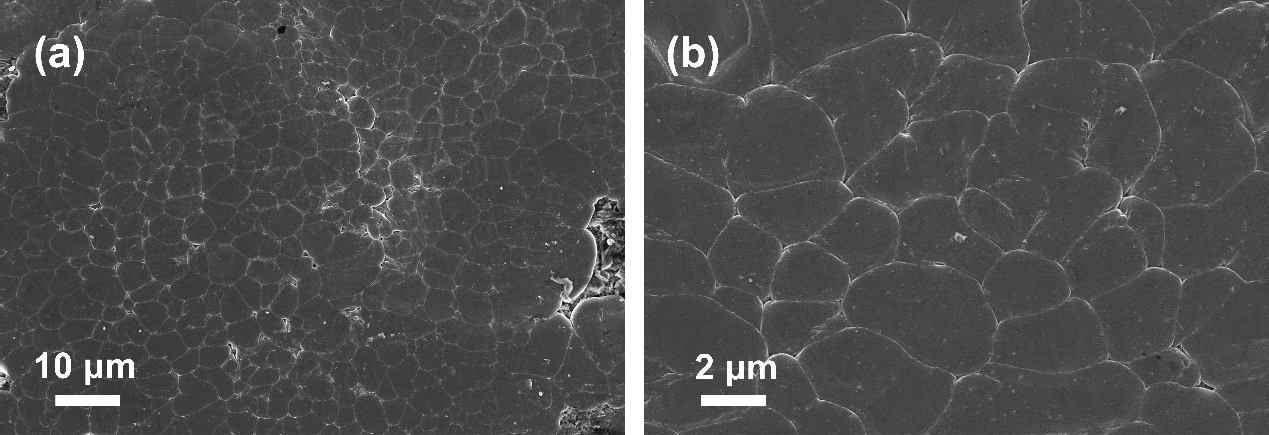


**Figure S18**. SEM images of Li metal anode covered by the POAmMe SEI layer.


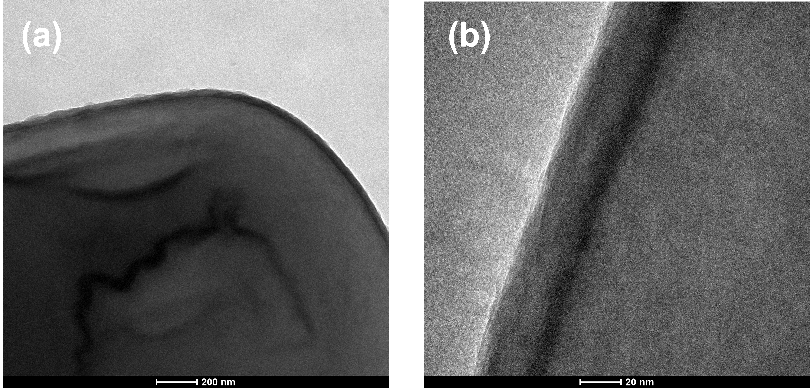


**Figure S19**. Cryogenic HR-TEM image demonstrating POAmMe hybrid SEI layer covered on one metallic Li nuclei.


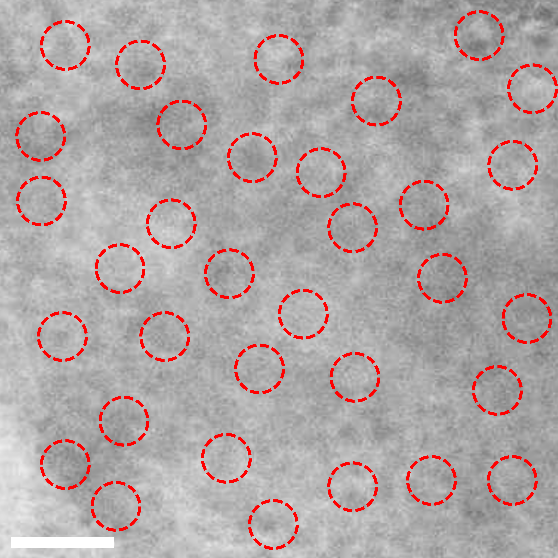


**Figure S20**. Cryogenic High-resolution TEM image of the SEI film incorporating the POAmMe 3D molecular framework. The red circles highlight the POSS-derived nanoscale domains (dark area). The scale bar represents 5 nm.


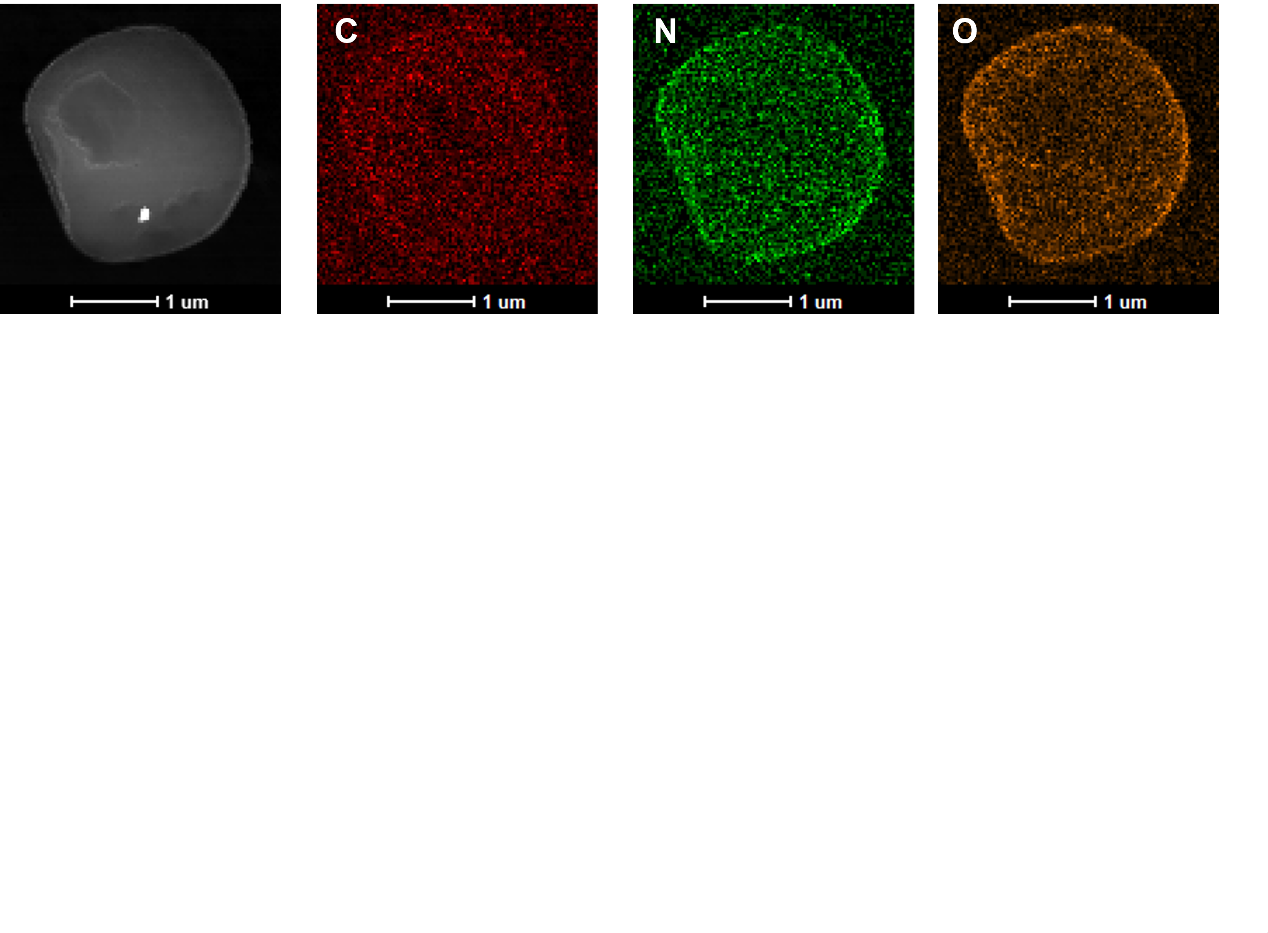


**Figure S21**. The EDS mapping illustrates the surface elemental distribution of a metallic Li nuclei encapsulated by the POAmMe-derived SEI layer.


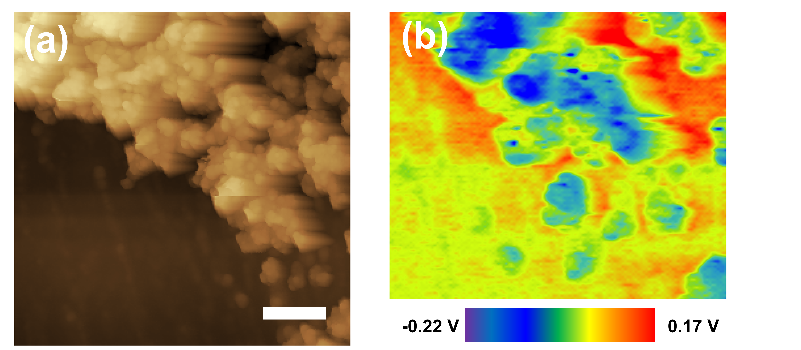


**Figure S22**. (a) Morphology and (b) surface potential distribution of lithium metal characterized by Kelvin probe force microscopy (KPFM). Scale bar (a), 500 nm.


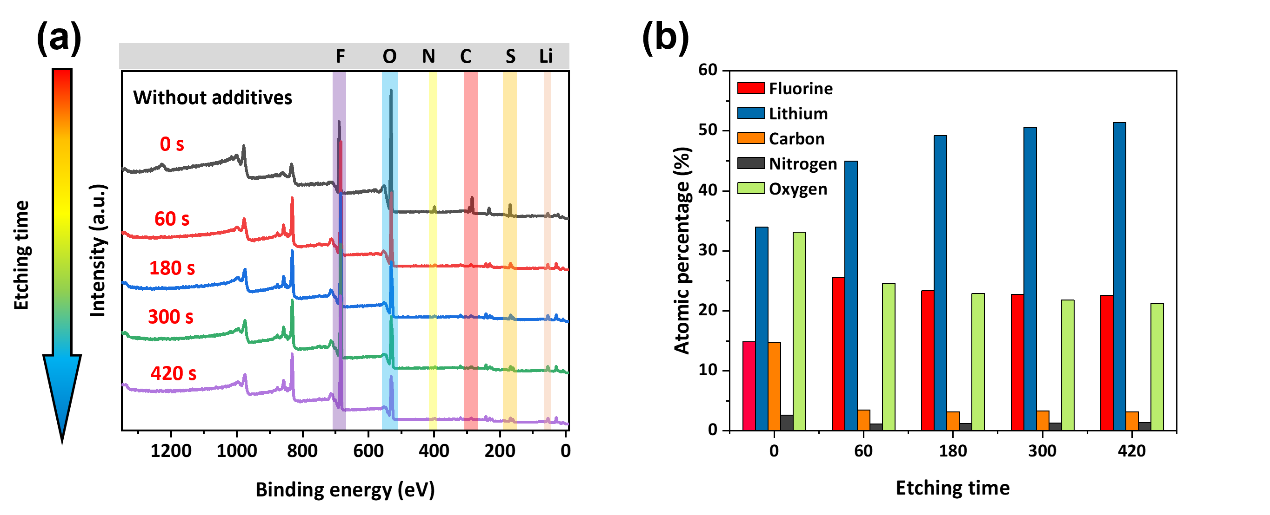


**Figure S23**. (a) XPS depth profiling of elemental full spectra of the original SEI layer at different etching times. (b) Comparison of XPS element ratio changes at different etching times.


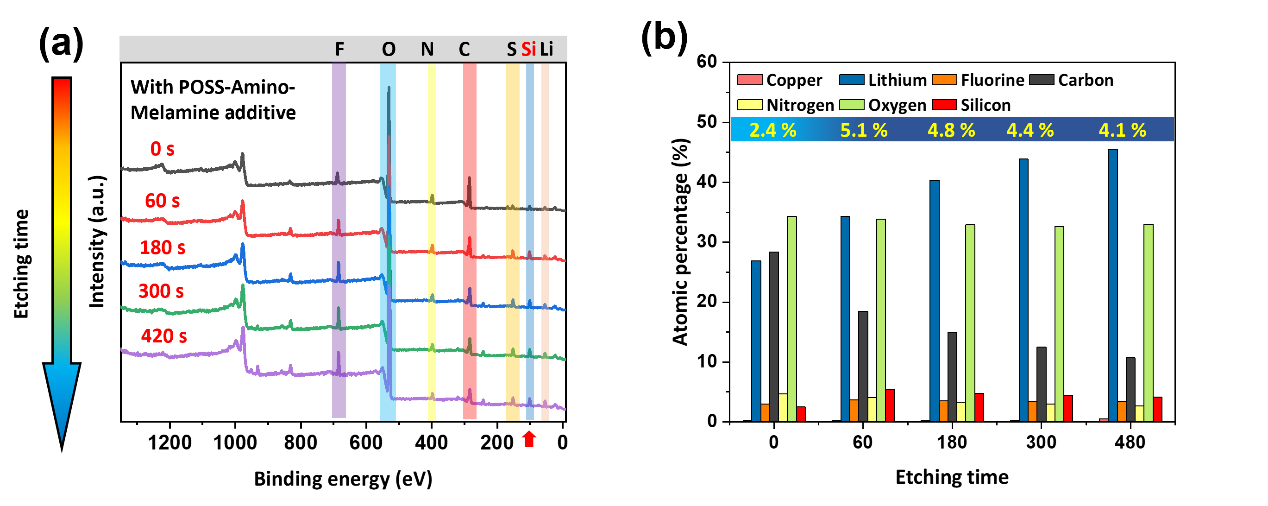


**Figure S24**. (a) XPS depth profiling of elemental full spectra of the POAmMe hybrid SEI layer at different etching times. (b) Comparison of XPS element ratio changes at different etching times.


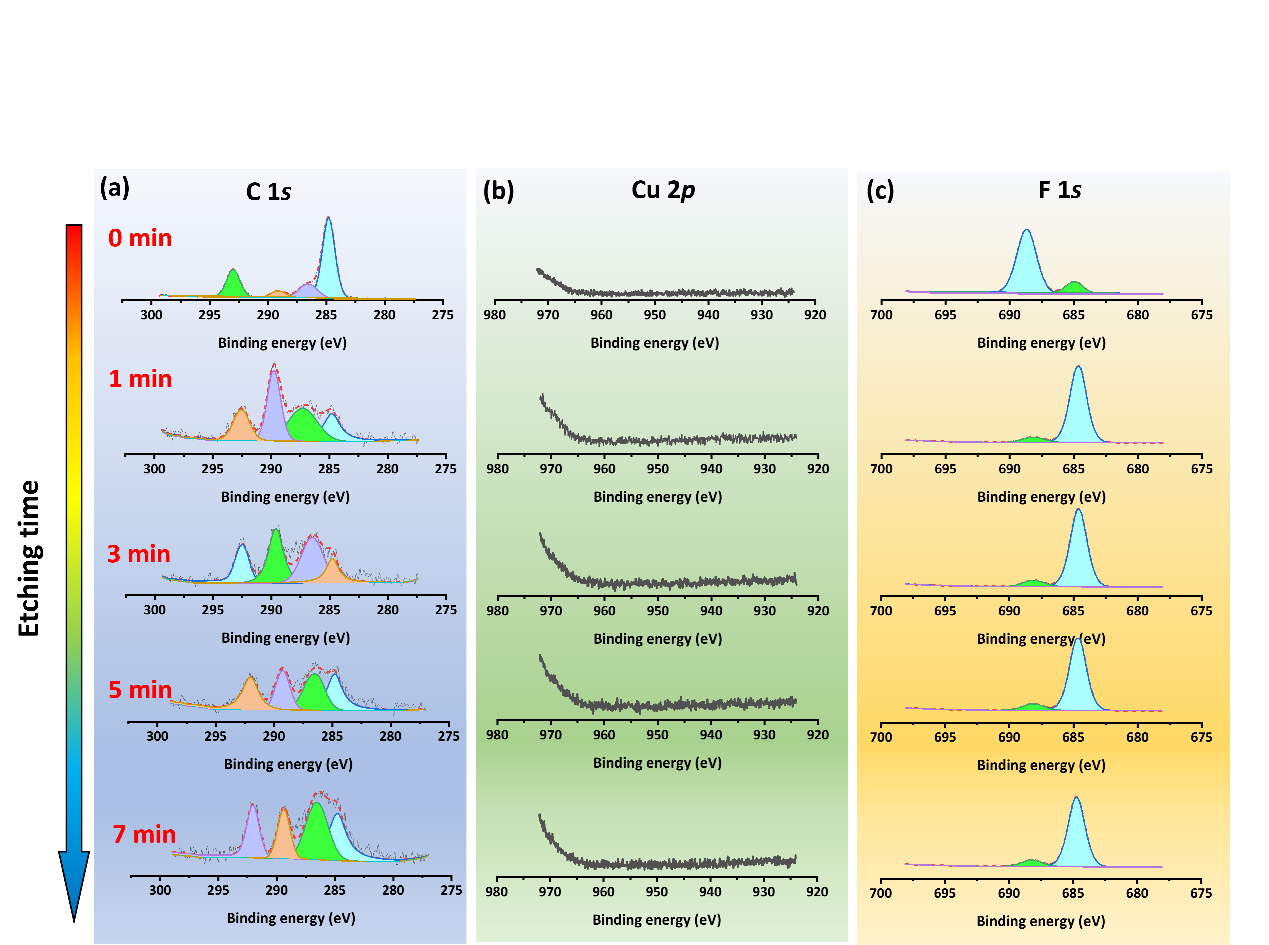


**Figure S25.** XPS Depth profiling of precise spectra of (a) C 1*s*, (b) Cu 2*p* and (c) F 1*s* of original SEI layer.


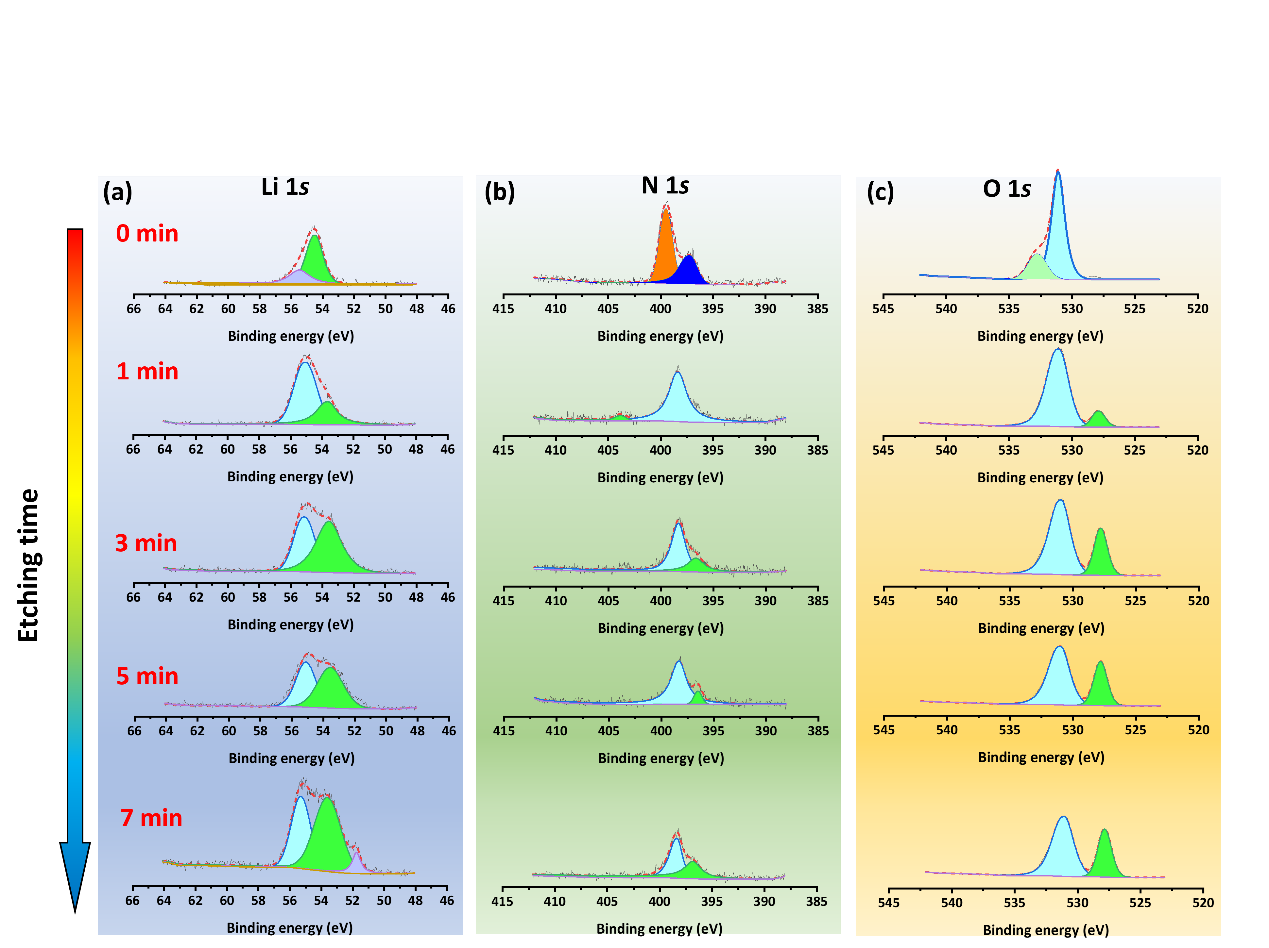


**Figure S26**. XPS Depth profiling of precise spectra of (a) Li 1*s*, (b) N 1*s* and (c) O 1*s* of original SEI layer.


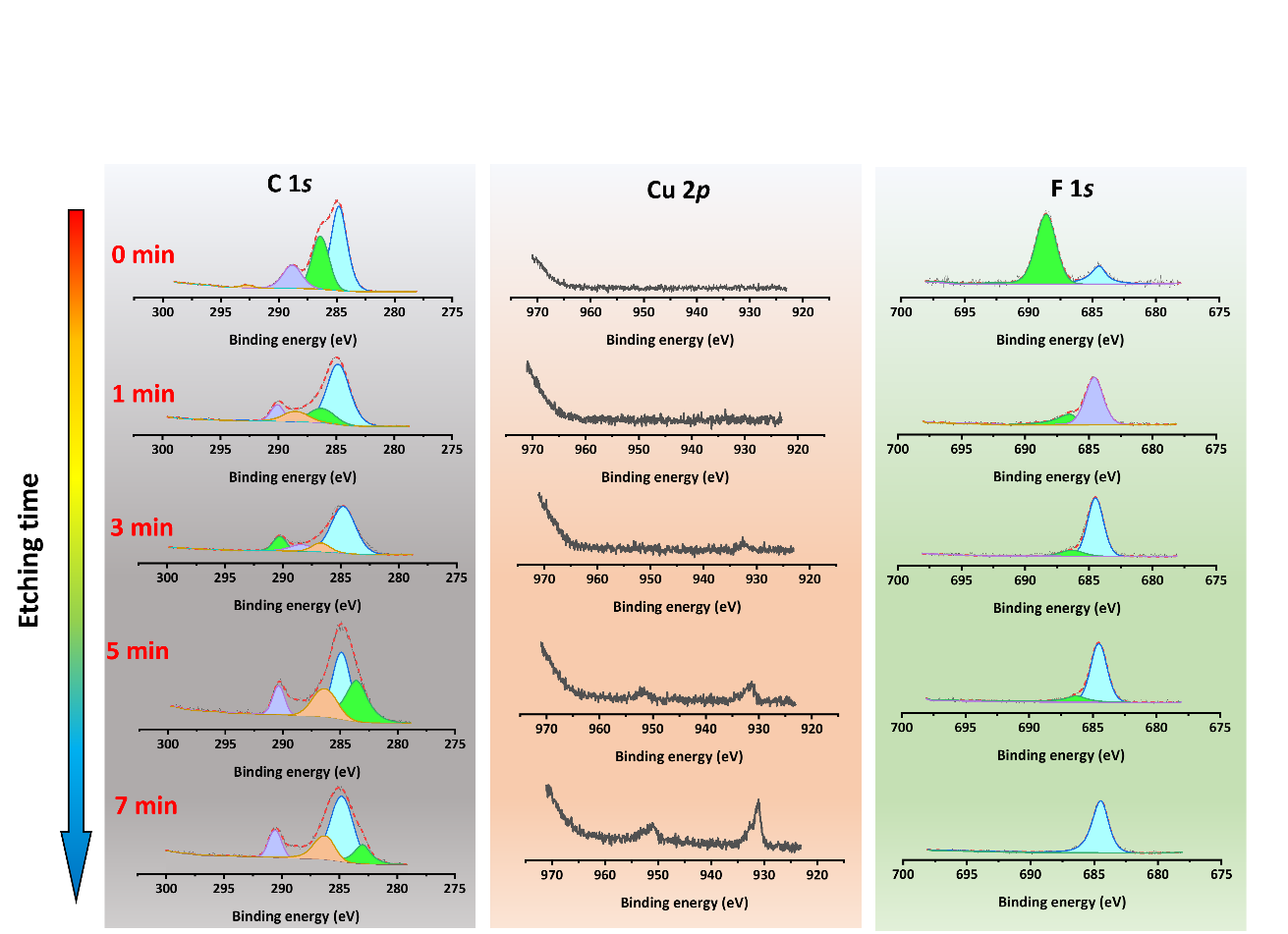


**Figure S27**. XPS Depth profiling of precise spectra of (a) C 1*s*, (b) Cu 2*p* and (c) F 1*s* of POAmMe hybrid SEI layer.


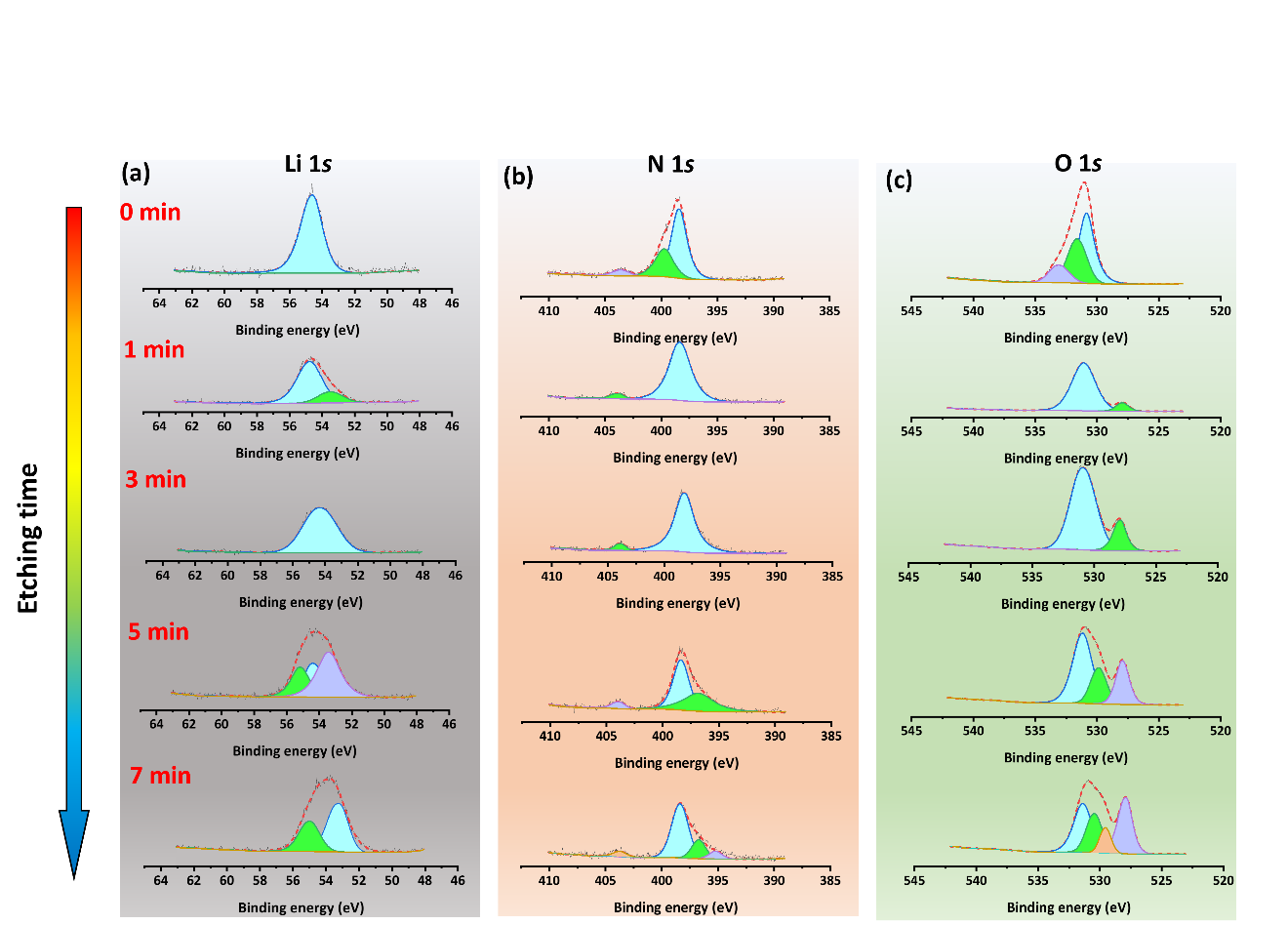


**Figure S28**. XPS Depth profiling of precise spectra of (a) Li 1*s*, (b) N 1*s* and (c) O 1*s* of POAmMe hybrid SEI layer.


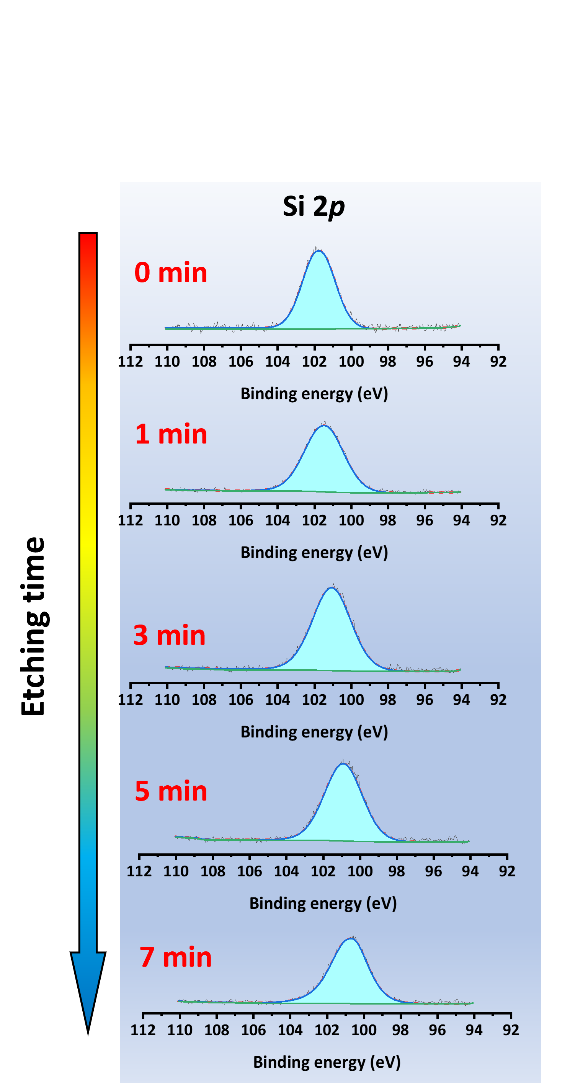


**Figure S29**. XPS Depth profiling of precise spectra of Si 2*p* of POAmMe hybrid SEI layer.


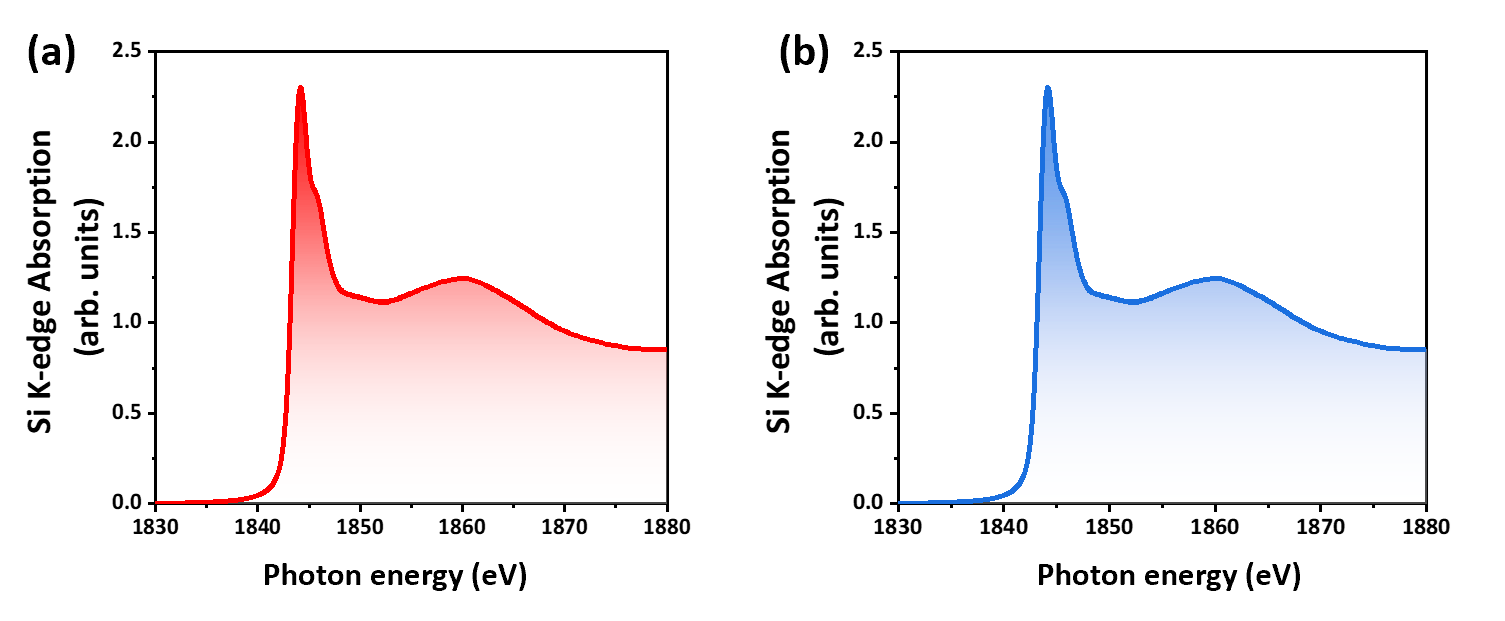


**Figure S30.** Si K-edge XANES spectra of POAmMe obtained before cycling (red) and after electrochemical cycling (blue).

**HOMO**
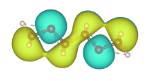

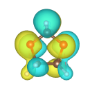

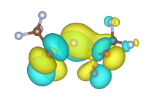

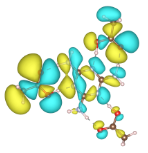


**LUMO**
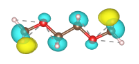

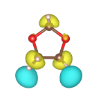

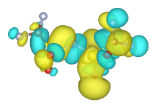

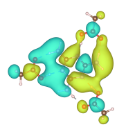


**DEM DOL TFSI Melamine trimer**

**Figure S31**. Frontier molecular orbitals (HOMO and LUMO) of DME, DOL, TFSI, and melamine trimer, showing the spatial distribution of electron density.

**Structures:**


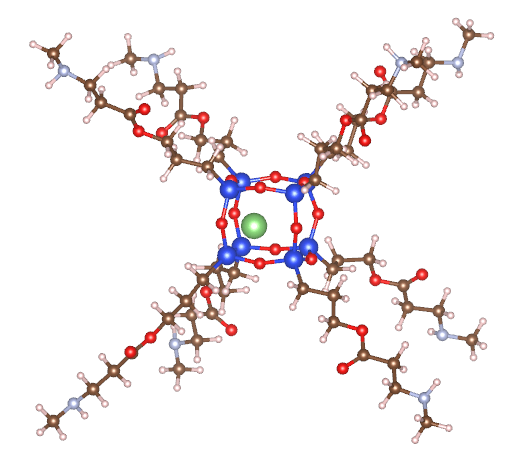

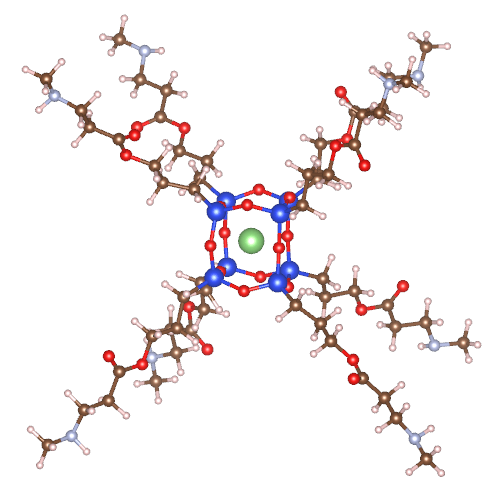

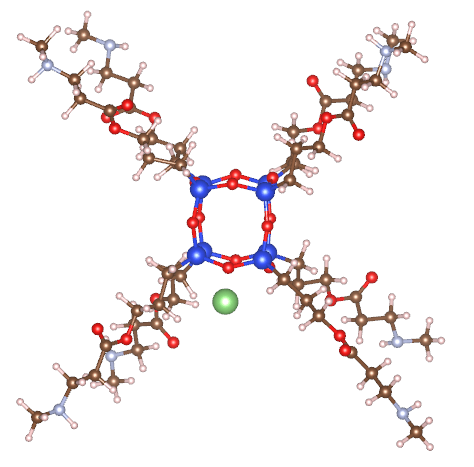


**Figure S32**. Adsorption configurations of POSS@Li at various sites (M: face-centered, T: body-centered, Z: axis-centered).


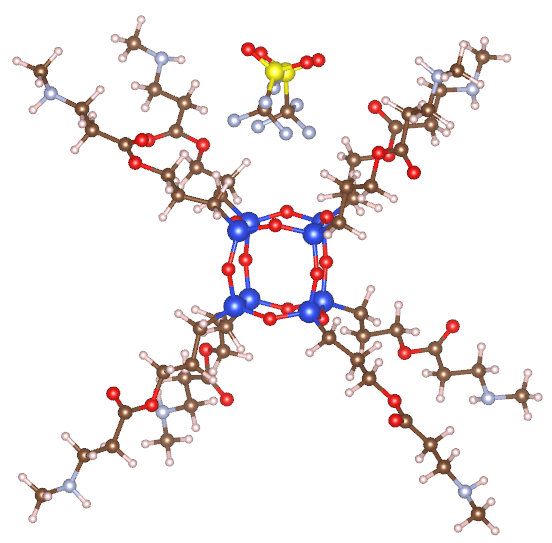

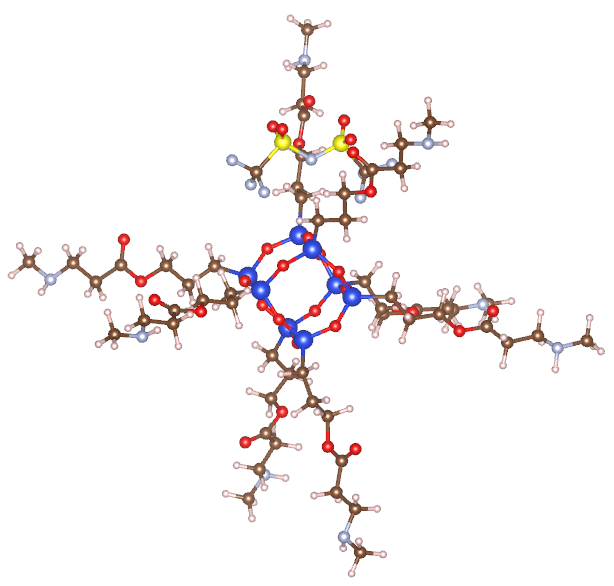


**Figure S33**. Optimized adsorption configurations of POSS@TFSI at representative sites, including face-centered (M), body-centered (T), and axis-centered (Z) positions.


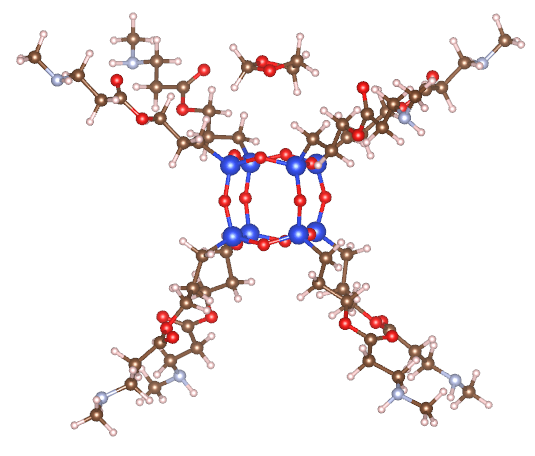


**Figure S34** Adsorption configurations of POSS@TFSI at various sites (M: face-centered, T: body-centered, Z: axis-centered).


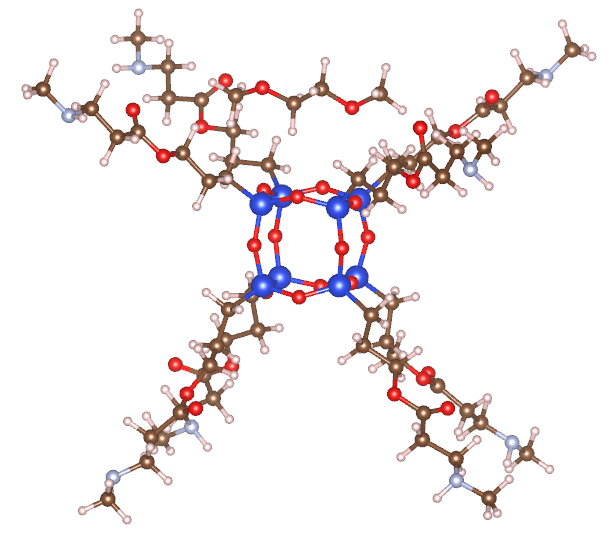


**Figure S35**. Absorption configurations of POSS@DME at various sites (M: face-centered, T: body-centered, Z: axis-centered).


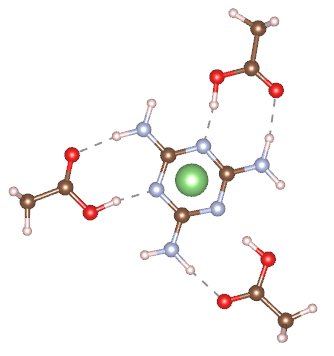

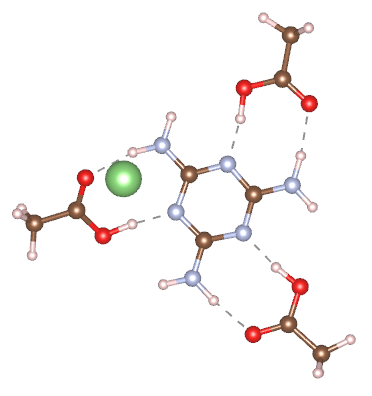


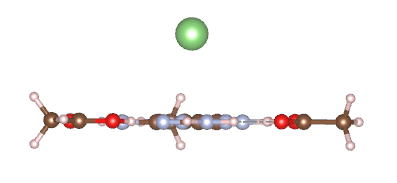

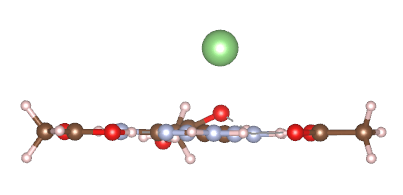


**Figure S36**. Optimized adsorption configurations of melamine trimer@Li at representative sites, including the axial plane (F) and the eight-membered ring plane (E).


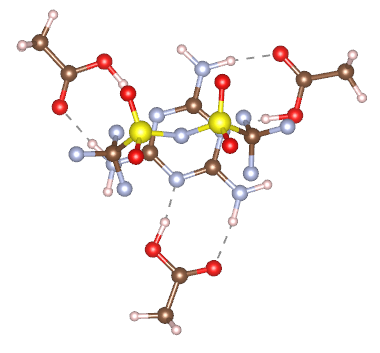

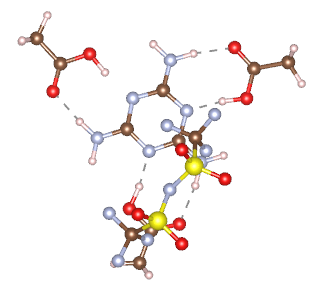


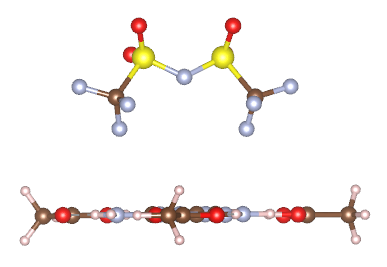

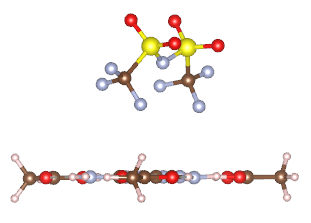


**Figure S37**. Optimized adsorption configurations of melamine trimer@TFSI at representative sites, including the axial plane (F) and the eight-membered ring plane (E).


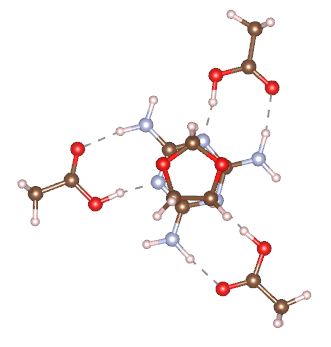

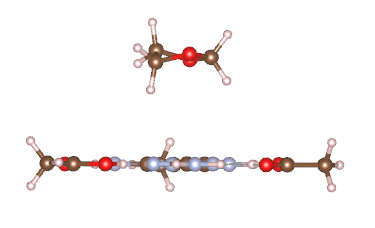


**Figure S38**. Optimized adsorption configurations of melamine trimer@DOL at representative sites, including the axial plane (F) and the eight-membered ring plane (E).


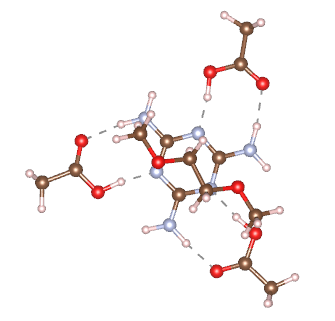

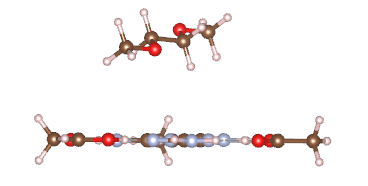


**Figure S39**. Adsorption configurations of melamine trimer@DME at various sites (F: axial plane, E: eight-membered ring plane).

**Table S1**. Calculated HOMO and LUMO energy levels of DME, DOL, TFSI, and melamine trimer molecules.

| **Systems** | **HOMO** | **LUMO** |
| --- | --- | --- |
| DEM | -5.8357 | -0.5126 |
| DOL | -5.8676 | -0.5380 |
| TFSI | -7.9751 | -3.6291 |
| Melamine trimer | -6.3088 | -1.4312 |
| POSS | -5.0023 | -1.1235 |

**Table S2**. Calculated adsorption energies of Li, TFSI, DOL, and DME at different adsorption sites on the POSS molecule.

| **systems** | **Position** | **E_ad_ (eV)** |
| --- | --- | --- |
| POSS@Li | M | -1.002 |
|  | T | -0.780 |
|  | Z | -0.205 |
| POSS@TFSI | M | -1.839 |
|  | Z | -2.767 |
| POSS@DOL | M | -0.242 |
| POSS@DME | M | -0.453 |

**Table S3**. Calculated adsorption energies of Li, TFSI, DOL, and DME at different adsorption sites on the melamine trimer molecule.

| **Systems** | **Position** | **E_ad_ (eV)** |
| --- | --- | --- |
| Mela@Li | F | -0.149 |
|  | E | -0.291 |
| Mela@TFSI | F | -0.430 |
|  | E | -0.399 |
| Mela @DOL | F | -0.158 |
| Mela @DME | F | -0.192 |


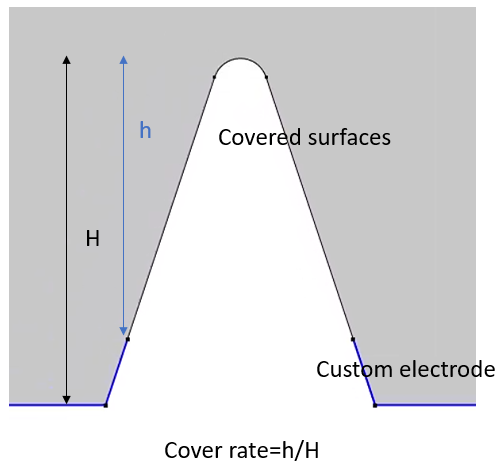


**Figure S40**. Schematic illustration of cross-sectional Li dendrite nucleation simulated by COMSOL.

| t=300s | Cover rate = 0 | Cover rate = 80 % |
| --- | --- | --- |
| Current density  (a.u.) | 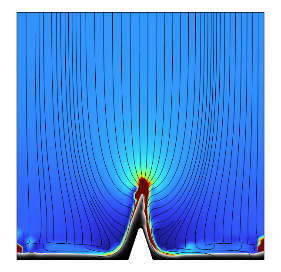 | 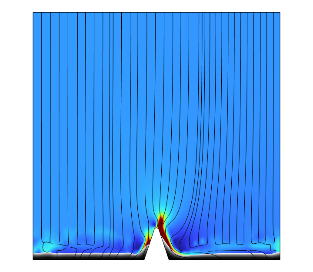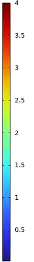 |
| Electric field  (V m^-1^) | 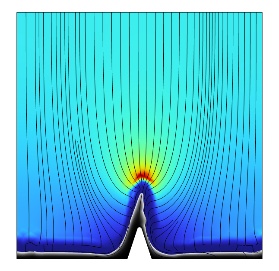 | 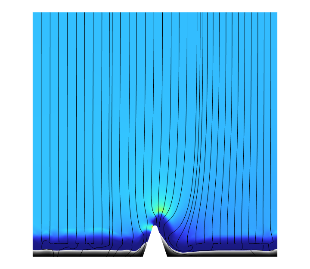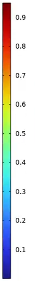 |
| t=600s | Cover rate = 0 | Cover rate = 80 % |
| Current density  (a.u.) | 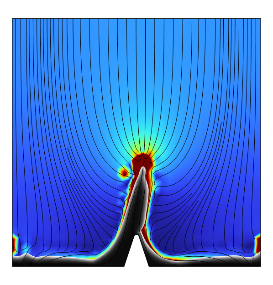 | 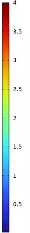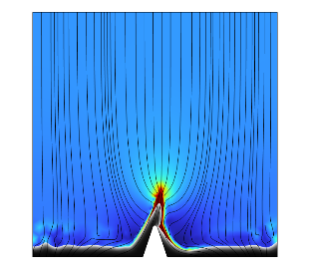 |
| Electric field  (V m^-1^) | 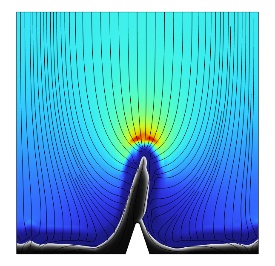 | 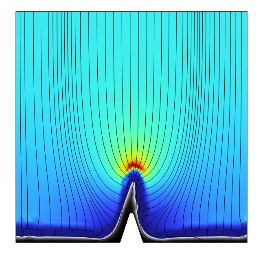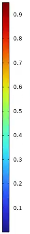 |

**Table S4**. COMSOL simulation of electric field redistribution and current densities around growing Li dendrites at 300 s and 600 s comparing native SEI and POAmMe-modified SEI conditions. (Cover rate = 0 and 0.8 is compared.)


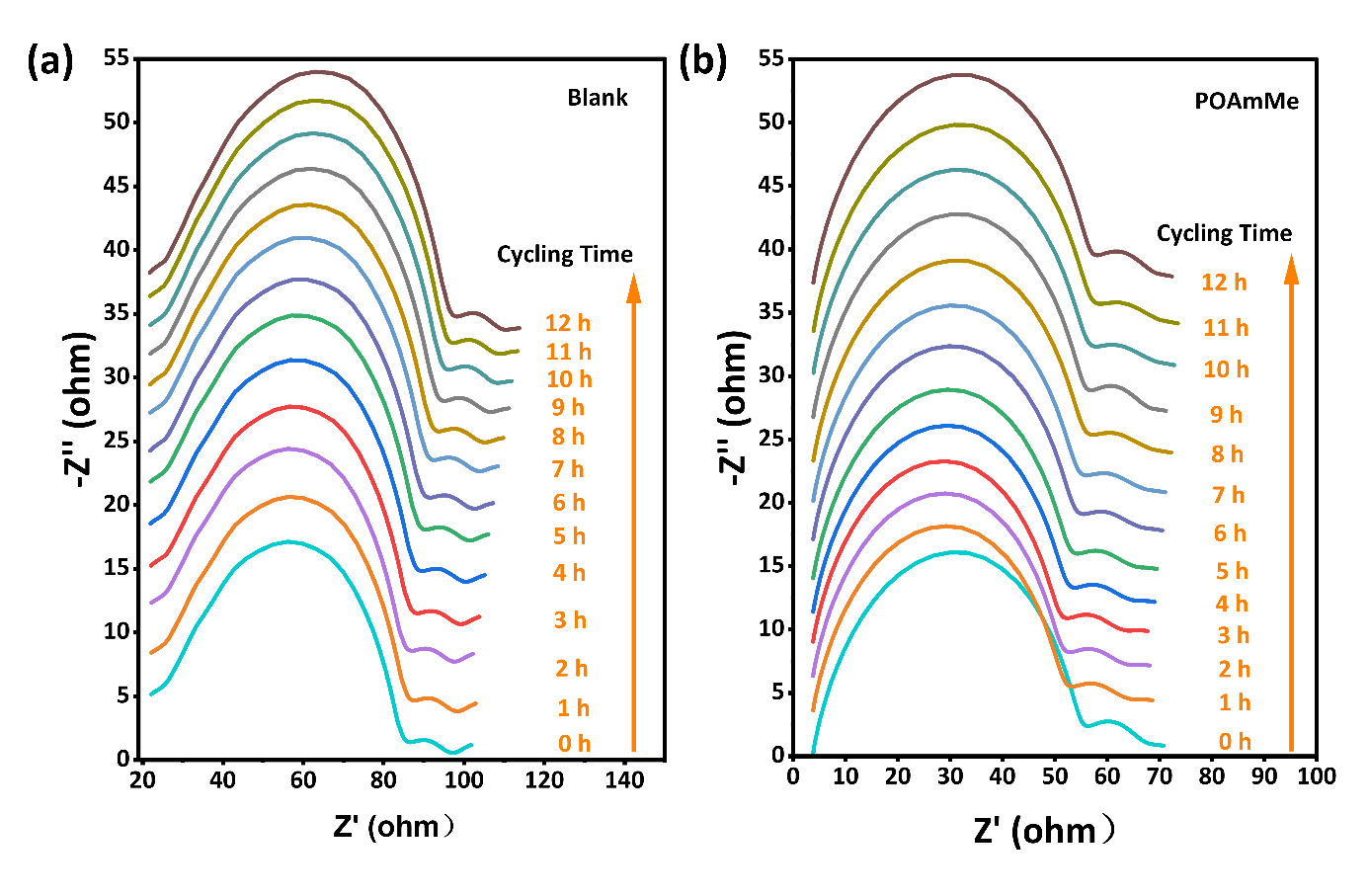


**Figure S41.** Time-resolved in situ EIS evolution of Li||Li symmetric cells during early Li plating/stripping. Nyquist plots of Li||Li symmetric cells using (a) blank electrolyte and (b) POAmMe-containing electrolyte collected from 0 to 12 h during cycling. The measurements were performed under the same plating/stripping conditions as those used for the symmetric-cell cycling test. The POAmMe-containing cell exhibits lower and more stable interfacial impedance than the blank cell, indicating the formation and reconstruction of a stable POAmMe-derived interphase on the Li surface during early cycling.


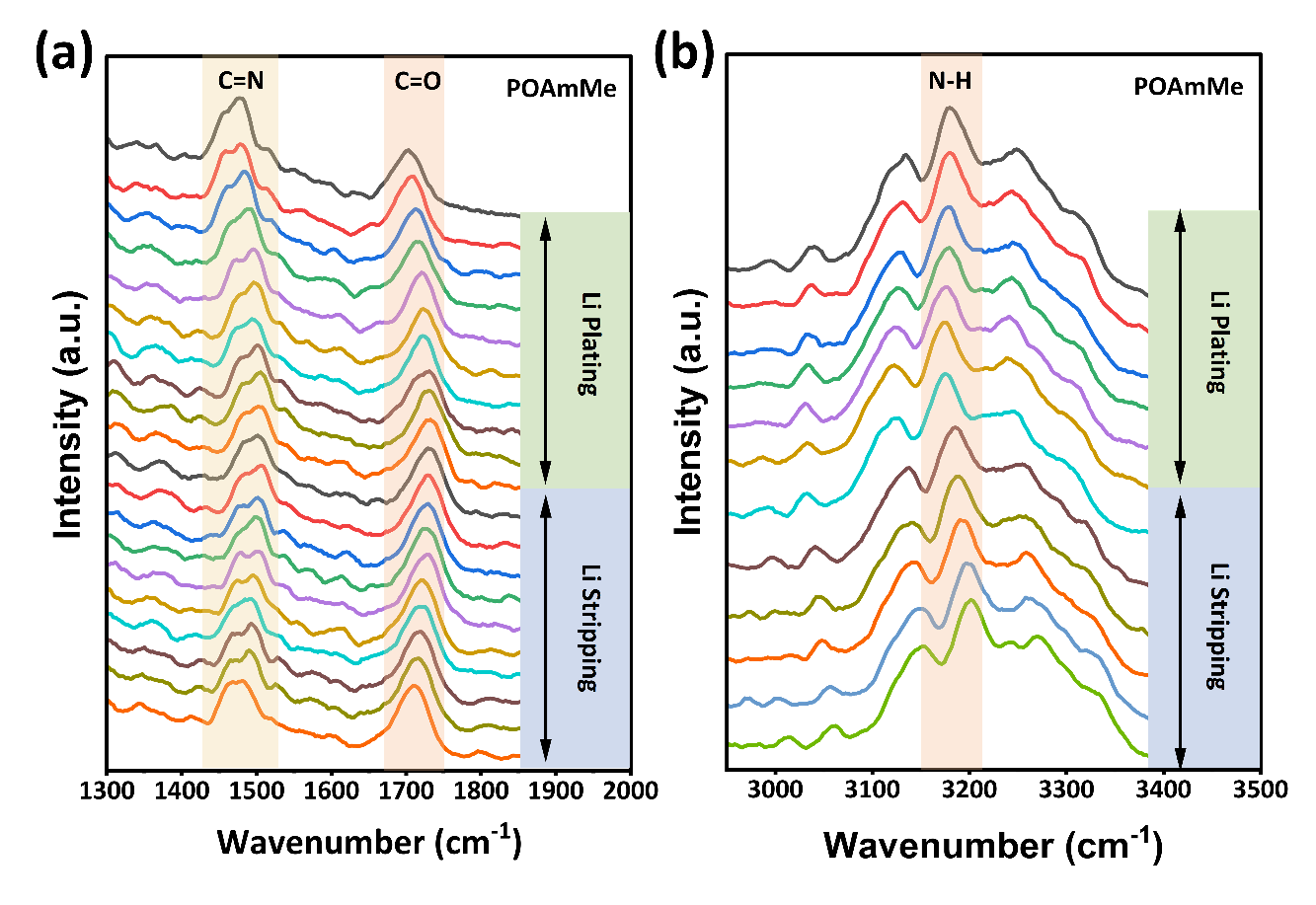


**Figure S42.** In situ Raman spectra of the POAmMe-containing Li||Li symmetric cell during Li plating and stripping. Enlarged Raman spectra in the regions of (a) 1300–2000 cm⁻¹ and (b) 2950–3500 cm⁻¹ collected from the monitored Li electrode during Li plating/stripping. The shaded regions highlight the C=N, C=O, and N–H related Raman bands associated with the POAmMe supramolecular framework. The reversible spectral variations during Li plating and stripping indicate stress-responsive rearrangement and reassociation of melamine-mediated hydrogen-bonded junctions in the POAmMe-derived interphase.


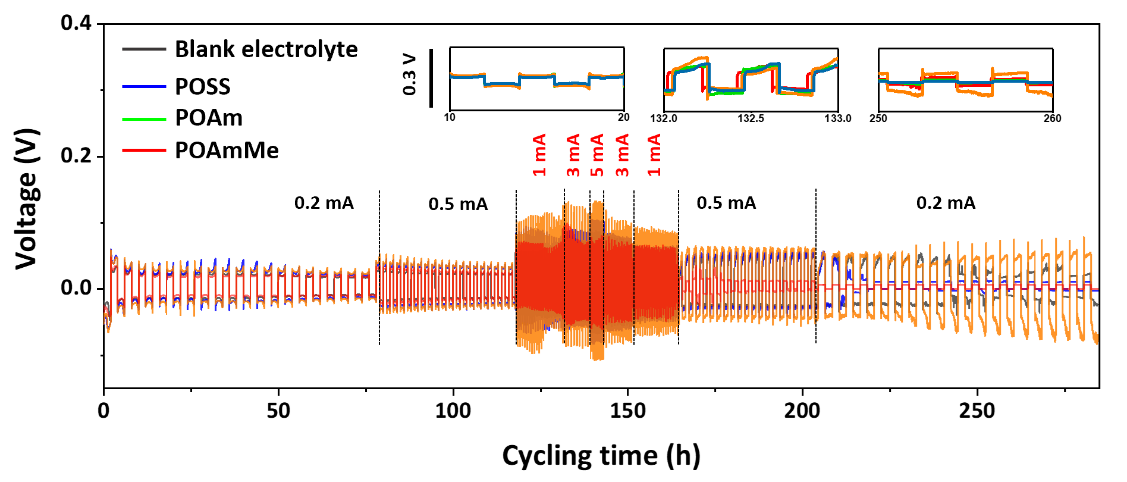


**Figure S43**. Comparison of galvanostatic Li plating/stripping profiles of Li || Li symmetric cells in blank electrolyte and electrolytes containing POSS, POAm, and POAmMe additives at different current densities.


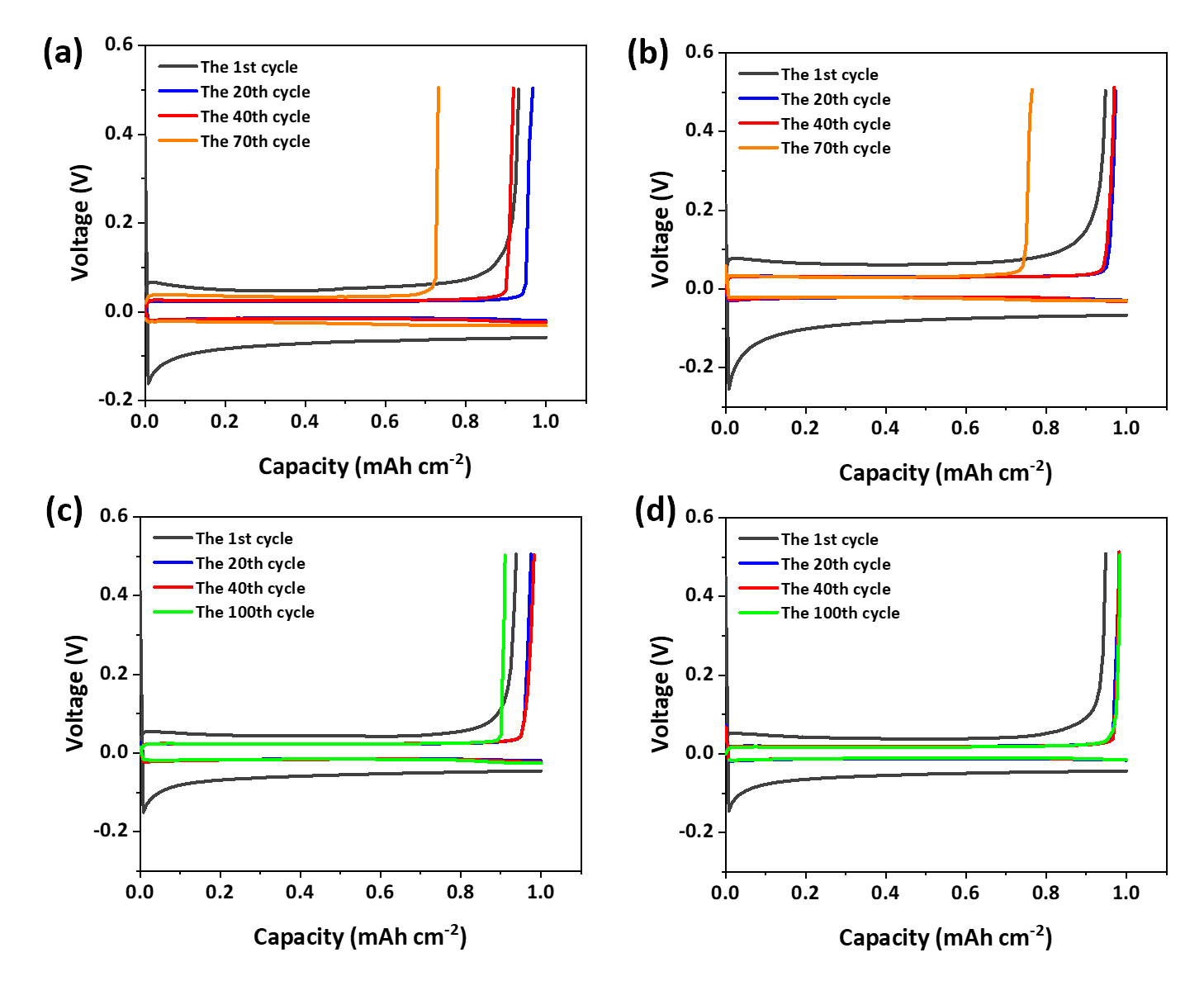


**Figure S44**. Discharge-charge curves of Li || Cu half cells using (a) blank electrolyte and that with (b) POSS, (c) POAm, (d) POAmMe additives after different cycles.


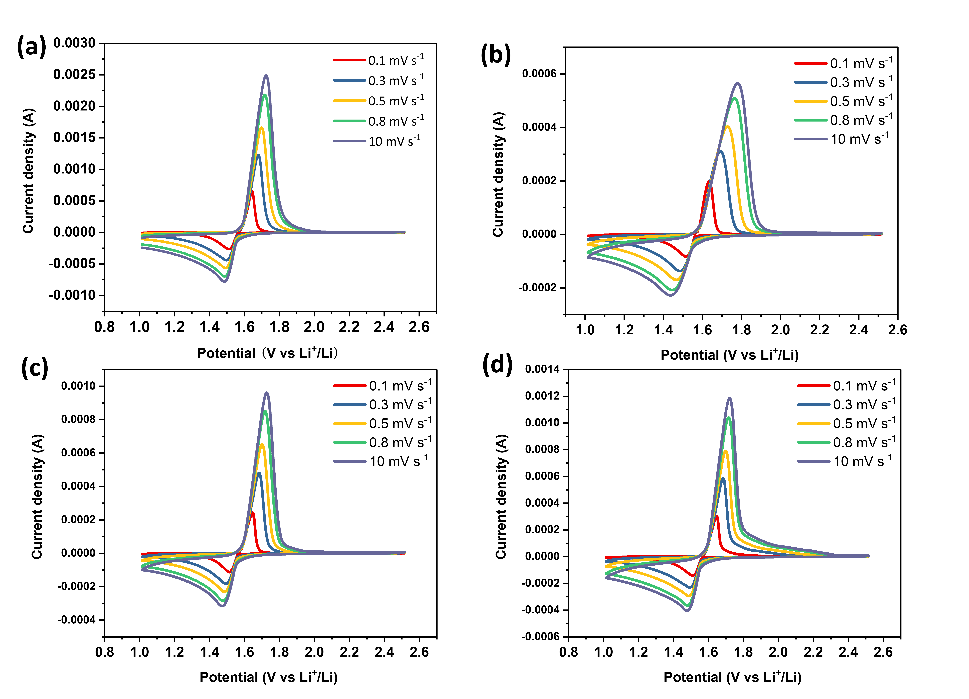


**Figure S45**. Cyclic voltammetry curves of Li || LTO cells using (a) blank electrolyte and that with (b) POSS, (c) POAm, (d) POAmMe additives at different scan rates.


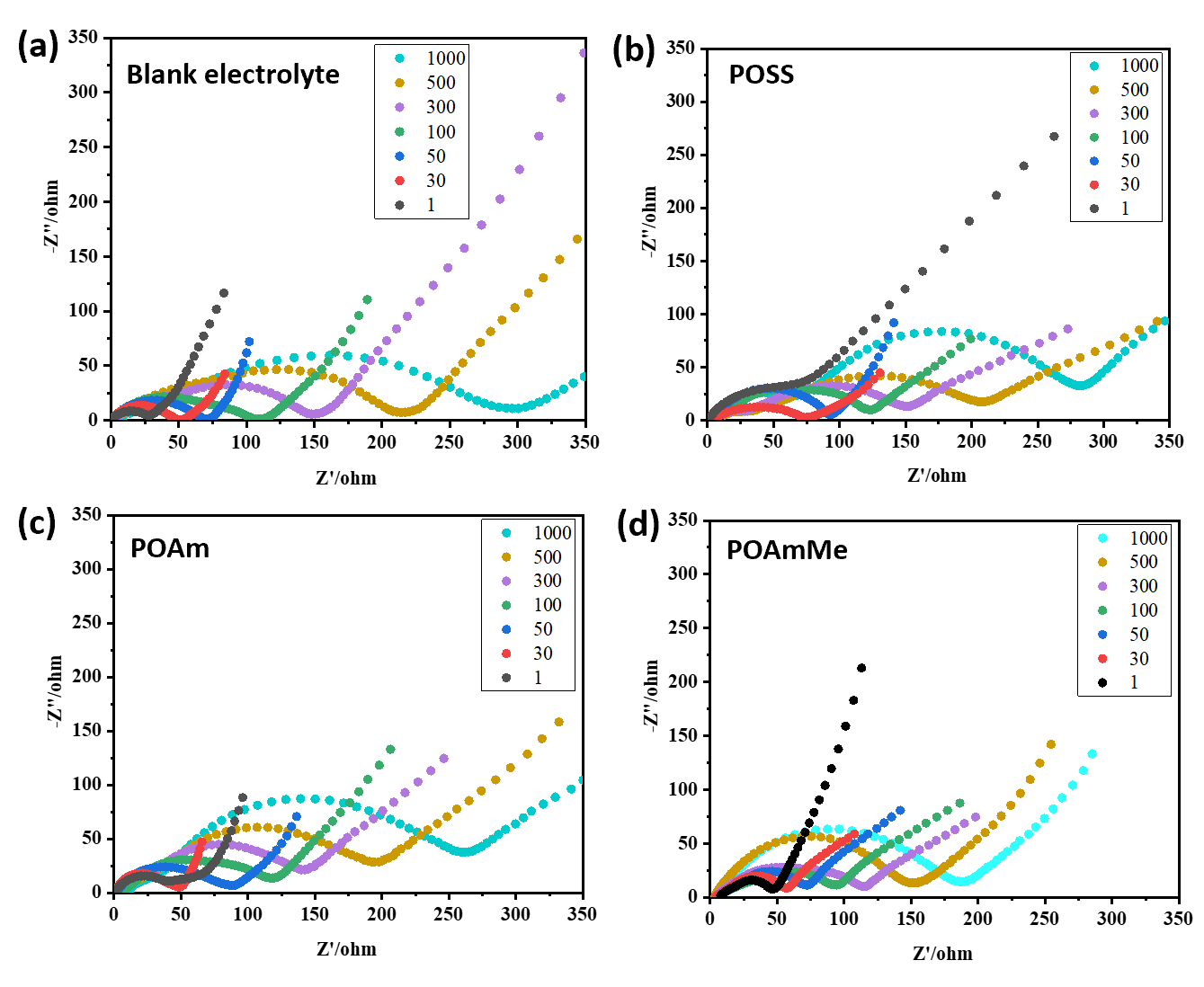


**Figure S46**. Electrochemical impedance spectroscopy curves of LTO || Li full cells using (a) blank electrolyte and that with (b) POSS, (c) POAm, (d) POAmMe additives after different cycles.


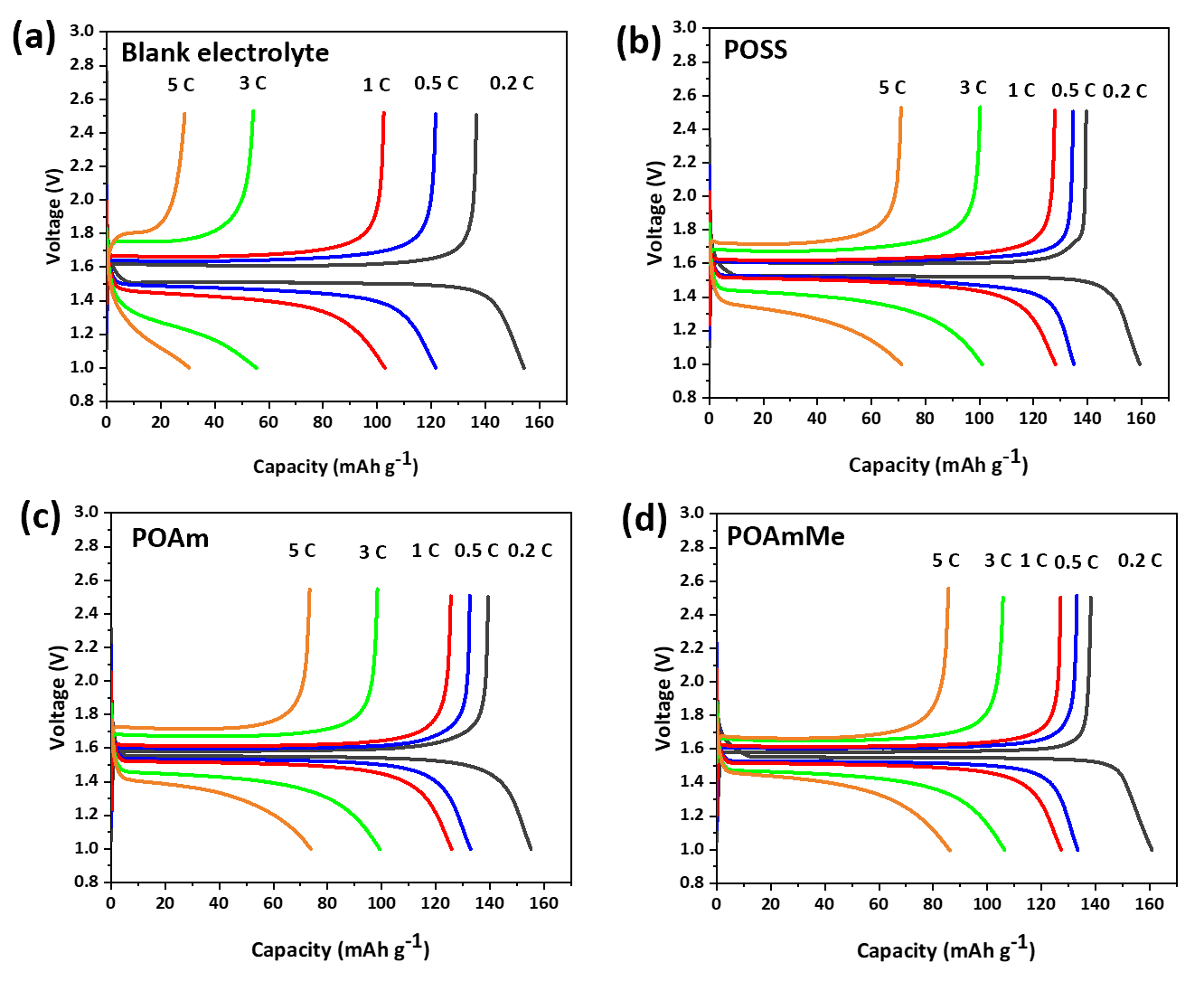


**Figure S47**. Discharge-charge curves of rate capabilities from 0.2 C to 5 C of the Li || LTO full cells using (a) blank electrolyte and that with (b) POSS, (c) POAm, (d) POAmMe additives.


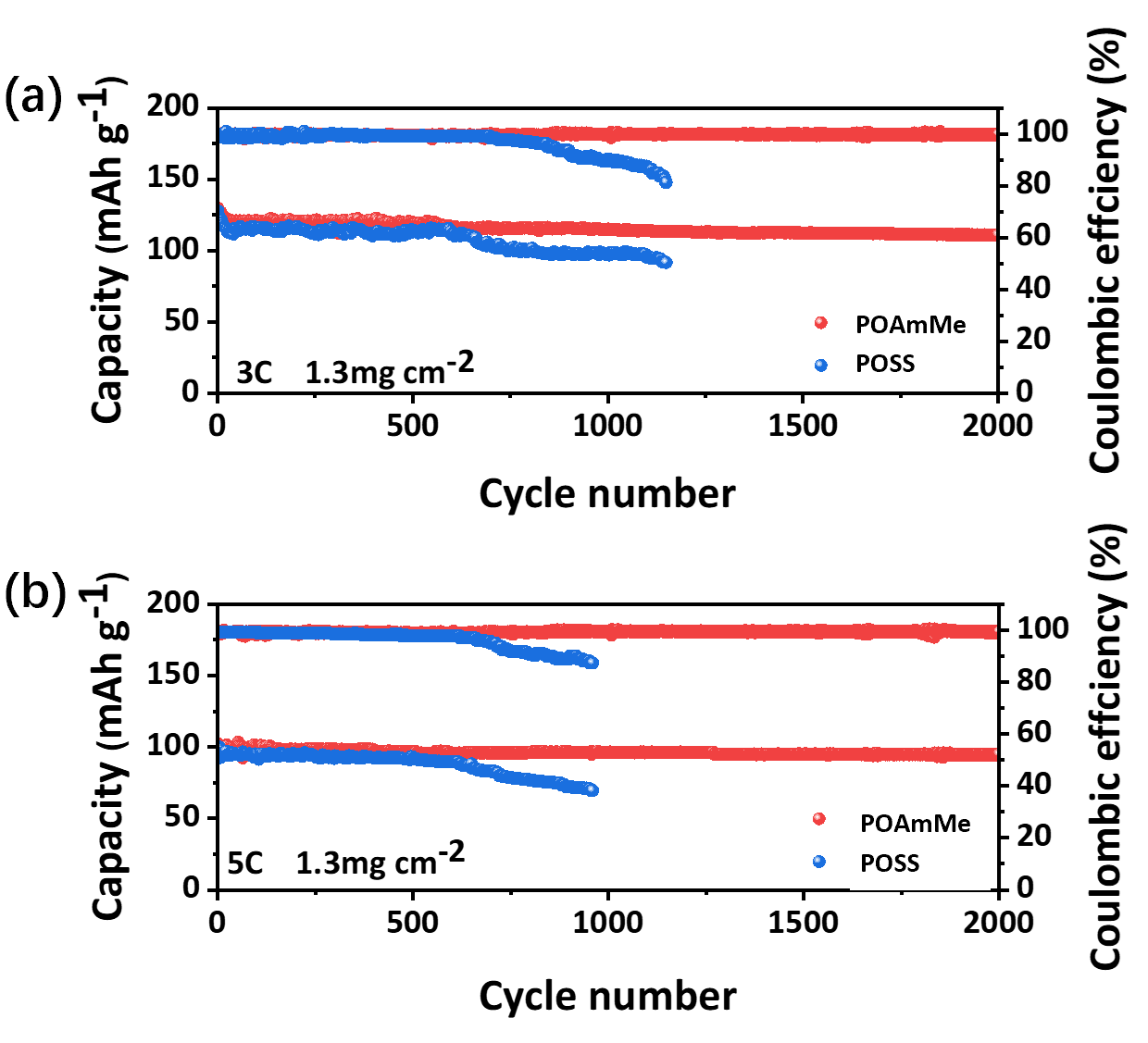


**Figure S48**. Cycling capacities of Li || LTO full cells using blank electrolyte and that with POAmMe additives at (a) 3 C and (b) 5 C.


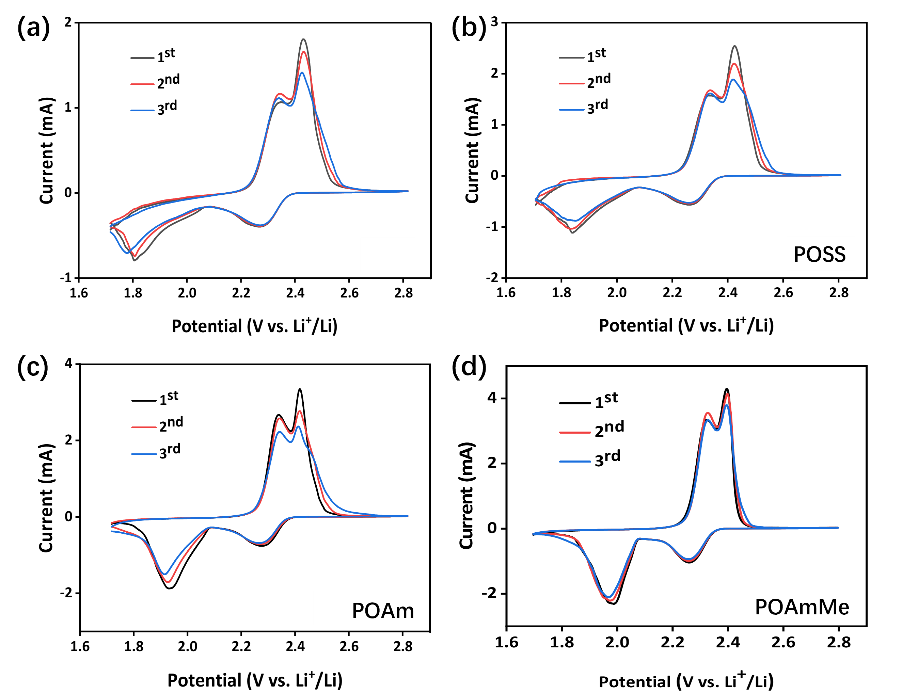


**Figure S49**. Cyclic voltammetry (CV) curves of Li || S full cells using blank electrolyte and that with POSS, POAm and POAmMe additives at different scan cycles.

**
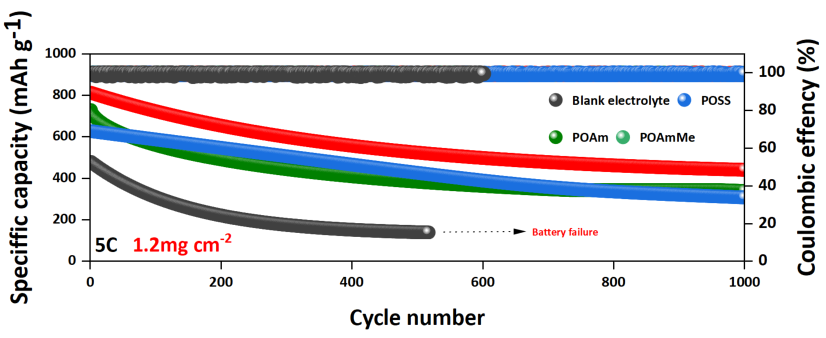
**

**Figure S50.** Comparison of galvanostatic charge–discharge cycling stabilities of Li || S full cells using blank electrolyte and that with POSS, POAm and POAmMe additives at current density of 5 C.

**
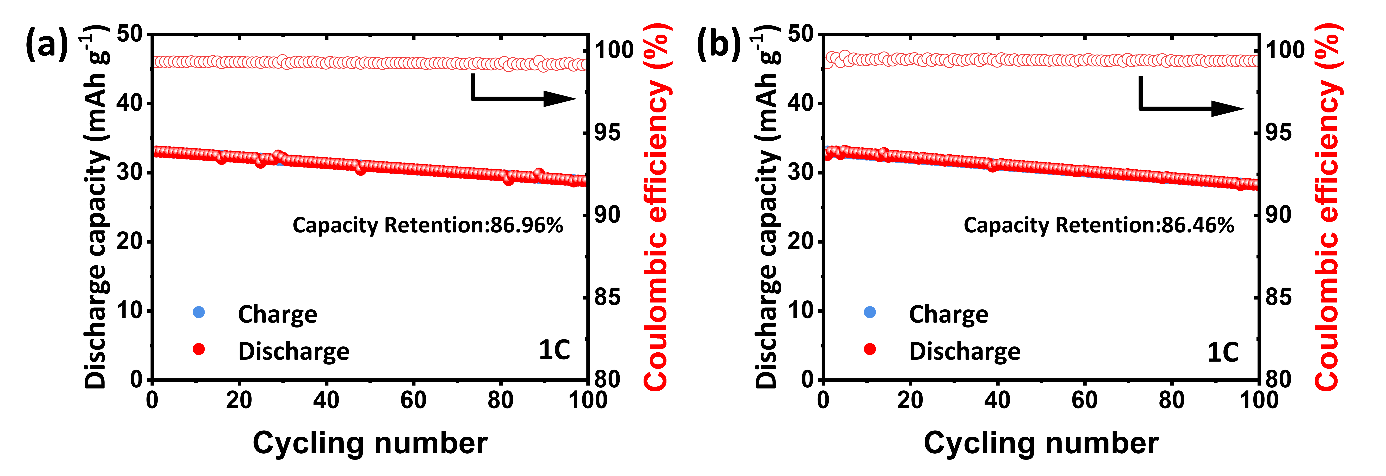
**

**Figure S51. Additional cycling runs of LFP||Li pouch cells with the POAmMe additive.** Cycling performance and Coulombic efficiency of two additional independently assembled LFP||Li pouch cells using ester-based electrolyte containing 0.5 wt% POAmMe at 1 C (178 mA g⁻¹). The LFP cathode loading was 180 mg. The additional cycling runs show consistent capacity retention and stable Coulombic efficiency, confirming the reproducibility and practical effectiveness of POAmMe in pouch-cell configurations.

**
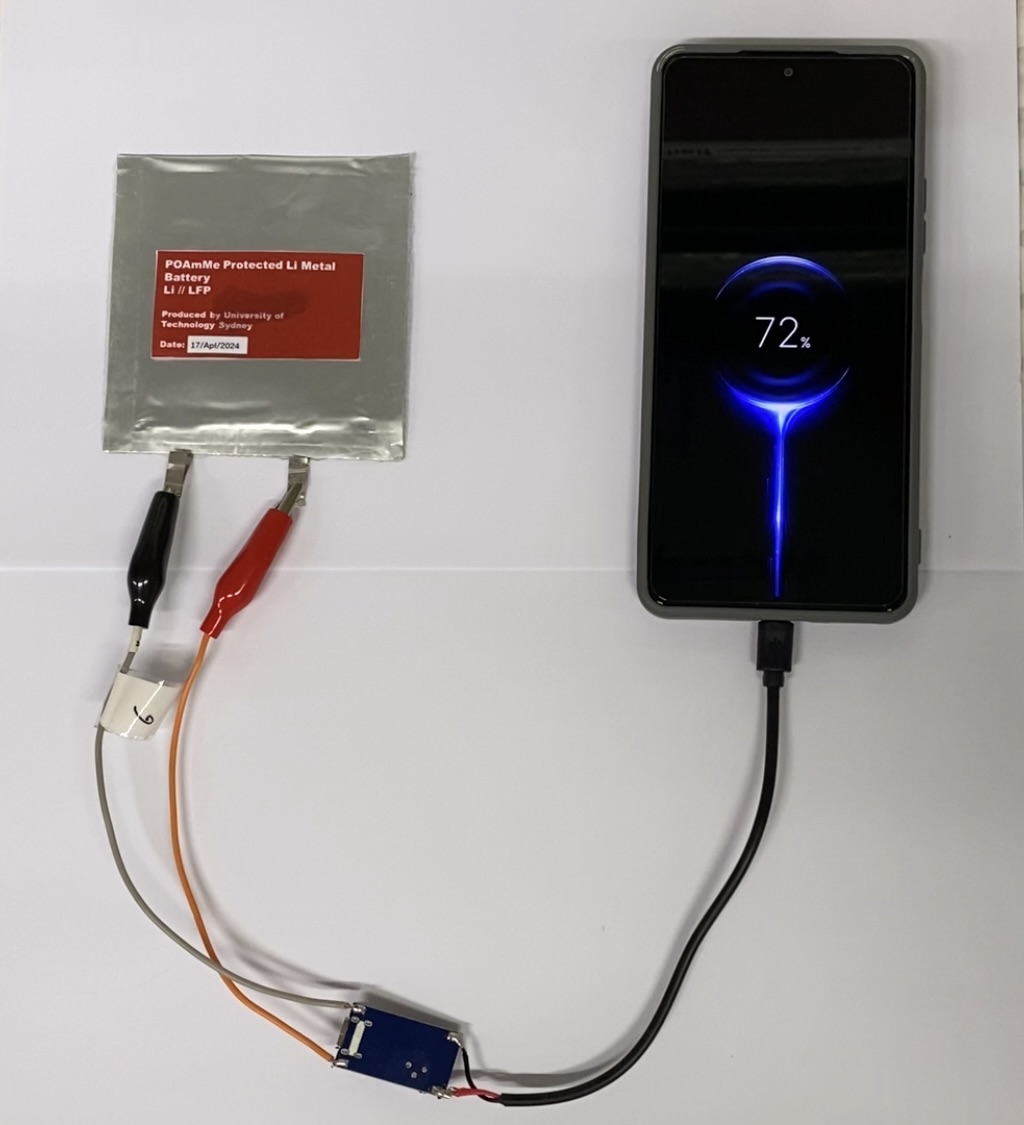
**

**Figure S52.** Demonstration of smartphone charging using an Li || LFP pouch cell with the POAmMe additive.

1. **References in supporting information**
2. P. Hohenberg, and W. Kohn, “Inhomogeneous Electron Gas,” *Physical Review* 136 (1964): B864-B871.
3. W. Kohn, and L. Sham, “Self-Consistent Equations Including Exchange and Correlation Effects,” *Physical Review* 140 (1965): A1133-A1138.
4. G. Kresse, and J. Furthmüller, “Efficient iterative schemes for ab initio total-energy calculations using a plane-wave basis set,” *Physical review B* 54 (1996): 11169.
5. P. Blöchl, “Projector augmented-wave method,” *Physical review B* 50 (1994): 17953-17979.
6. J. Perdew, K. Burke, and M. Ernzerhof, “Generalized gradient approximation made simple,” *Physical Review Letters* 77 (1996): 3865.
7. H. Monkhorst, and J. Pack, “Special points for Brillouin-zone integrations,” *Physical review B* 13 (1976): 5188-5192.
8. V. Wang, N. Xu, J.-C. Liu, G. Tang, and W.-T. Geng, “VASPKIT: A user-friendly interface facilitating high-throughput computing and analysis using VASP code,” *Computer Physics Communications* 267 (2021): 108033.
9. S. Grimme, J. Antony, S. Ehrlich, and H. Krieg, “A consistent and accurate ab initio parametrization of density functional dispersion correction (DFT-D) for the 94 elements H-Pu,” *Journal of Chemical Physics* 132 (2010): 154104.
